# A phosphatase‐centric mechanism drives stress signaling response

**DOI:** 10.15252/embr.202152476

**Published:** 2021-09-24

**Authors:** David Maria Hollenstein, Gabriela Gérecová, Natalie Romanov, Jessica Ferrari, Jiri Veis, Marion Janschitz, Reinhard Beyer, Christoph Schüller, Egon Ogris, Markus Hartl, Gustav Ammerer, Wolfgang Reiter

**Affiliations:** ^1^ Department of Biochemistry and Cell Biology Max Perutz Labs Vienna BioCenter (VBC) University of Vienna Vienna Austria; ^2^ Max Planck Institute of Biophysics Frankfurt am Main Germany; ^3^ Center for Medical Biochemistry Max Perutz Labs, Vienna BioCenter Medical University of Vienna Vienna Austria; ^4^ Department of Applied Genetics and Cell Biology (DAGZ) University of Natural Resources and Life Sciences (BOKU) Vienna Austria; ^5^ Research Platform Bioactive Microbial Metabolites (BiMM) Tulln a.d. Donau Austria; ^6^ Mass Spectrometry Facility Max Perutz Labs, Vienna BioCenter University of Vienna Vienna Austria

**Keywords:** endosulfine, greatwall kinase, hyperosmotic stress signaling, phosphatase, PP2A, Post-translational Modifications & Proteolysis, Proteomics, Signal Transduction

## Abstract

Changing environmental cues lead to the adjustment of cellular physiology by phosphorylation signaling networks that typically center around kinases as active effectors and phosphatases as antagonistic elements. Here, we report a signaling mechanism that reverses this principle. Using the hyperosmotic stress response in *Saccharomyces cerevisiae* as a model system, we find that a phosphatase‐driven mechanism causes induction of phosphorylation. The key activating step that triggers this phospho‐proteomic response is the Endosulfine‐mediated inhibition of protein phosphatase 2A‐Cdc55 (PP2A^Cdc55^), while we do not observe concurrent kinase activation. In fact, many of the stress‐induced phosphorylation sites appear to be direct substrates of the phosphatase, rendering PP2A^Cdc55^ the main downstream effector of a signaling response that operates in parallel and independent of the well‐established kinase‐centric stress signaling pathways. This response affects multiple cellular processes and is required for stress survival. Our results demonstrate how a phosphatase can assume the role of active downstream effectors during signaling and allow re‐evaluating the impact of phosphatases on shaping the phosphorylome.

## Graphical abstract

The phosphatase PP2A^Cdc55^ assumes the role of an active downstream effector during hyperosmotic stress signaling in *Saccharomyces cerevisiae*. Inhibition of the phosphatase is the key event resulting in increased phosphorylation, while induction of kinases affecting the same substrates appears not to be required.

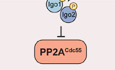

## Introduction

Cells respond to external and internal stimuli by activation of complex signaling networks that are typically characterized by highly conserved, intertwined signaling pathways. Both signal propagation and the eventual integration mechanisms need to be fine‐tuned for the cell to adequately respond to specific situations. The most common mechanism by which a signal is transmitted within the cell is reversible protein phosphorylation. The addition and removal of the phosphate moiety can modulate protein functionality, as well as its activity, localization, and protein interactions. Dysregulation of signaling networks, i.e., perturbations of the phosphorylome, can have disastrous consequences and represent hallmarks of many diseases (Grundke‐Iqbal *et al*, 1986; Meyerovitch *et al*, 1989; Blume‐Jensen & Hunter, 2001; Eidenmüller *et al*, 2001; Ozcan *et al*, 2004; Rikova *et al*, 2007; Zanivan *et al*, 2013). It is therefore pivotal to investigate phosphorylation and its effects on regulatory networks to fully understand cell behavior, as well as health and disease.

In human cells, up to 65% of expressed proteins get phosphorylated (Keshava Prasad *et al*, 2009; Vlastaridis *et al*, 2017; Yu *et al*, 2019), with more than 100,000 distinct phosphorylation events recorded (Vlastaridis *et al*, 2017; Needham *et al*, 2019). However, the regulatory underpinning of 95% of the phosphorylome is still unknown (Needham *et al*, 2019). Almost all efforts to explain the regulation of phosphorylation are centered around protein kinases, directing our understanding of signaling pathways toward a kinase‐centric view.

The contribution of protein phosphatases in defining the state of the phosphorylome, on the other hand, has largely not been investigated in‐depth. This is due to the fact that phosphatases have been less studied than kinases overall and are typically not considered to be effector proteins of signaling networks (Brautigan, 2013; Smoly *et al*, 2017). The majority of studies highlight a rather passive role of phosphatases in terminating signaling responses or in counteracting a kinase signaling cascade. However, increasing evidence suggests an intricate regulation of activity and composition of protein phosphatase complexes and a highly specific substrate recognition (Virshup & Shenolikar, 2009; Mishra *et al*, 2017; Courtney & Deiters, 2019). It is therefore plausible to assume that phosphatases could be more actively and specifically involved in determining the state of the phosphorylome.

Here, we report such an active role for the protein phosphatase 2A (PP2A) in the hyperosmotic stress response of *Saccharomyces cerevisiae,* a paradigm signaling response (Hohmann, 2002; Saito & Posas, 2012; Brewster & Gustin, 2014). PP2A, one of the most prominent protein phosphatases, is highly conserved among eukaryotes and has been associated with key cellular processes, such as cell cycle progression, apoptosis, cellular metabolism, and migration (Wlodarchak & Xing, 2016; Ferrari *et al*, 2017; Fabbrizi *et al*, 2018). The PP2A holo‐enzyme is a multi‐protein complex, consisting of a catalytic, a scaffolding, and a regulatory subunit. Substrate specificity is defined by the so‐called regulatory B subunits, in yeast named Cdc55 and Rts1 (Shu *et al*, 1997; Zhao *et al*, 1997; Wu *et al*, 2000; Wei *et al*, 2001; Yabe *et al*, 2018; Játiva *et al*, 2019).

In this study, we reveal a phosphatase‐centric signaling response based on the inhibition of PP2A^Cdc55^, triggering a wide range of stress‐induced phosphorylations that in unstressed conditions are kept at a low level by PP2A^Cdc55^. The inhibition of PP2A^Cdc55^ works through a conserved module composed of the Greatwall Kinase Rim15 and the Endosulfines Igo1 and Igo2, which represent specific inhibitors of PP2A (Gharbi‐Ayachi *et al*, 2010; Mochida *et al*, 2010). Phosphorylation of Igo1 and Igo2 by Rim15 promotes their binding to PP2A^Cdc55^, which results in inhibition of the phosphatase by a mechanism termed unfair competition (Williams *et al*, 2014; Thai *et al*, 2017). Our results suggest that a substantial set of stress‐induced phosphorylation sites is affected by the phosphatase in a direct manner, rendering the phosphatase a main downstream signaling effector of the stress response. This phosphatase‐driven pathway operates in parallel to and independent of the well‐established MAP kinase signaling pathway that is generally considered to be the main regulator of hyperosmotic stress signaling in yeast (Hohmann, 2002; Saito & Posas, 2012; Brewster & Gustin, 2014). The PP2A^Cdc55^ signaling response is essential for stress survival and required for efficient transcription of stress‐associated genes. Our findings therefore point toward an unexpected signaling mechanism, based on the inhibition of a phosphatase instead of activation of a kinase as the central effector component of a stress‐induced signal transduction pathway.

## Results

### Identification of the PP2A‐affected phosphorylome

**Figure 1:**
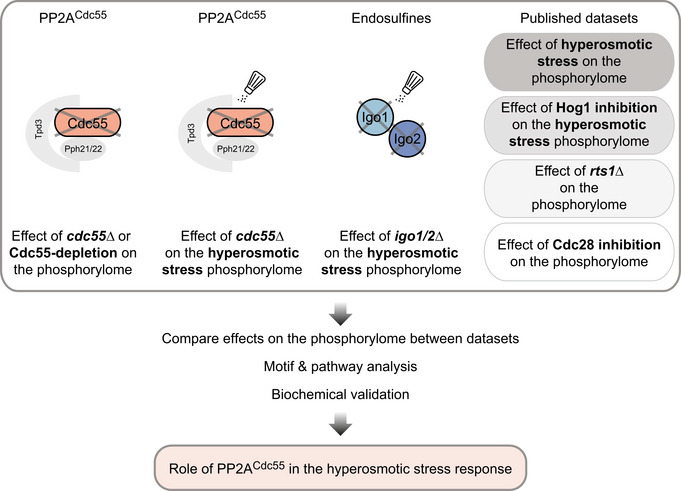
Experimental design to identify PP2A^Cdc55^ effects on the yeast phosphoproteome upon hyperosmotic stress Quantitative MS‐based phospho‐proteomic experiments used in this study. Experiments performed in this study compared the phosphoproteome of a wild‐type and a *CDC55* deletion (*n* = 8) or depletion strain (*n* = 4), and the effect of hyperosmotic stress (0.5 M NaCl, 10 min) on global phosphorylation patterns between a wild‐type strain and a *CDC55* (*n* = 2) or an *IGO1/IGO1* deletion (*n* = 3) strain. Published MS datasets that were integrated (indicated in the upper right) capture the early response to hyperosmotic stress (0.5 M NaCl, 5 min), and the effect of Hog1 inhibition on the early response to hyperosmotic stress (0.5 M NaCl, 5 min) (Romanov *et al*, 2017; Data ref: Romanov *et al*, 2017), the effect of a deletion of *RTS1* (preprint: Hollenstein *et al*, 2020) and the effect of Cdc28 inhibition (Kanshin *et al*, 2017) on the global phosphoproteome. All SILAC ratios represent knockout versus wild‐type, stressed versus unstressed, or inhibited versus not inhibited. After integration of the datasets, further analyses were performed using motif & GO enrichment, as well as *in vivo* biochemical validation.

Cdc55 and Rts1, the two regulatory subunits of the yeast phosphatase PP2A, have been previously linked to the hyperosmotic stress response (Evangelista *et al*, 1996; Zhao *et al*, 1997; Santhanam *et al*, 2004; Reiter *et al*, 2013). However, the extent to which protein phosphorylation patterns are globally affected by both, PP2A activity and hyperosmotic stress signaling, has not been analyzed before. Here, we used an unbiased mass spectrometry (MS) setup to capture a comprehensive set of potential PP2A substrates exhibiting altered phosphorylation patterns upon hyperosmotic stress. These were followed up through further biochemical validation (Fig 1, Table EV1).

**Figure EV1:**
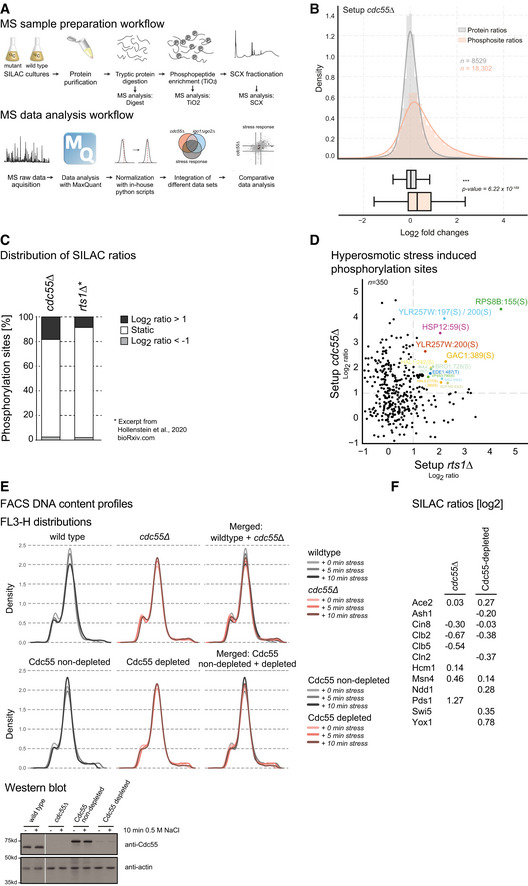
Experimental workflow and quality control (related to Fig 1) ASchematic illustration of the applied experimental workflow. Yeast cells were isotopically labeled using SILAC. Proteins were extracted using the TRIzol (Invitrogen) reagent and digested with trypsin. Phosphopeptides were enriched using TiO_2_ beads and fractionated offline by strong cation exchange chromatography (SCX). Peptide samples were analyzed by LC‐MS/MS, using an ESI‐LTQ‐Orbitrap Velos or ESI‐Q Exactive HF system (Thermo Scientific Fisher). The lower panel of the scheme illustrates the MS data analysis workflow, which was done with MaxQuant. After normalization of SILAC ratios, MS datasets were integrated and compared. SILAC ratios of phosphorylation sites were log2‐transformed with positive values indicating increased phosphorylation in the knockout and *vice versa*.BProtein abundance is not affected by deletion of *CDC55*, with 95% of all SILAC ratios clustering between −1 and 1 (log2). Histograms and boxplots illustrate SILAC ratios of unphosphorylated (gray) and phosphorylated peptides (red) from setup *cdc55*∆. *P*‐values were calculated using a *t*‐test.CBar plot displaying percentage of SILAC ratios stratified according to their value (ratio ≥ 2, ratio ≤ 0.5, and static) in setup *cdc55∆* and *‐ rts1∆*.DScatter plot comparing SILAC ratios of stress‐induced phosphorylation sites (≥ 2‐fold change in *SR*) between setups ‐ *cdc55*∆ (*y*‐axis) and ‐ *rts1*∆ (*x*‐axis). Commonly regulated phosphorylation sites are illustrated in colors and annotated.EWild‐type and *cdc55∆* cells or Cdc55‐depleted and non‐depleted cells were exposed for 0, 5, and 10 min to hyperosmotic stress (0.5 M NaCl). Cell cycle distribution was monitored by fluorescence‐activated cell sorting (FACS) analysis of DNA content. Representative FACS profiles are shown (upper). Western blot analysis was used to validate Cdc55 depletion (lower).FTable presenting log2 SILAC ratios from setup *cdc55∆* or Cdc55 depletion across sites of cell cycle‐related markers defined by Ref. (Kelliher *et al*, 2018).

We first performed large‐scale SILAC‐based quantitative MS experiments (Ficarro *et al*, 2002; Ong *et al*, 2002) assessing the impact of a *cdc55∆* deletion mutant on the phosphoproteome, further referred to as setup *cdc55∆* (Fig EV1A). In a parallel study (preprint: Hollenstein *et al*, 2020), we conducted similar experiments with cells lacking Rts1, designated as setup *rts1∆*. The reproducibility of the SILAC ratios for the phosphorylation sites demonstrates the quality and consistency of the MS datasets (Fig EV1B and Appendix Fig S1, see also Methods—“MS study design”). Setup *cdc55∆* encompassed 51 liquid chromatography (LC)‐MS‐runs, quantifying 10,790 phosphorylation sites (Dataset EV1). To identify phosphorylation sites that were up‐ or downregulated, we applied a twofold cutoff. 22.1% of the phosphorylation sites showed an increase in the deletion mutant, whereas only 4.3% showed a decrease and 73.6% remained static (Fig EV1C). Thus, most of the affected sites were prone to become increasingly phosphorylated, which is consistent with the expected impact of phosphatase inactivation. Our dataset* *also recovered findings from previous *CDC55* deletion studies as we found a multitude of known PP2A^Cdc55^ substrates to be increasingly phosphorylated, such as Sic1 (Moreno‐Torres *et al*, 2015), Gis1 (Bontron *et al*, 2013), Net1 (Queralt *et al*, 2006; Wang & Ng, 2006), Mih1 (Minshull *et al*, 1996; Wang & Burke, 1997), Swe1 (Minshull *et al*, 1996; Wang & Burke, 1997), Sli15 (Godfrey *et al*, 2017), and Ndd1 (Godfrey *et al*, 2017).

### PP2A^Cdc55^ signaling is central in shaping the phosphorylome during hyperosmotic stress

To elucidate the contribution of PP2A to the hyperosmotic stress response, we compared changes in the phosphoproteome observed in the setups *cdc55∆* and *rts1∆* (preprint: Hollenstein *et al*, 2020) with published MS datasets capturing phosphorylation changes during the early response to hyperosmotic conditions (Romanov *et al*, 2017; Data ref: Romanov *et al*, 2017) (Fig 1). The stress dataset, denoted as setup *stress response (SR)*, showed a considerable overlap of quantified phosphorylation sites with the *cdc55∆* (4,962 unique sites) and *rts1∆* (3,770 unique sites) MS experiments, allowing an integrative analysis.

**Figure 2:**
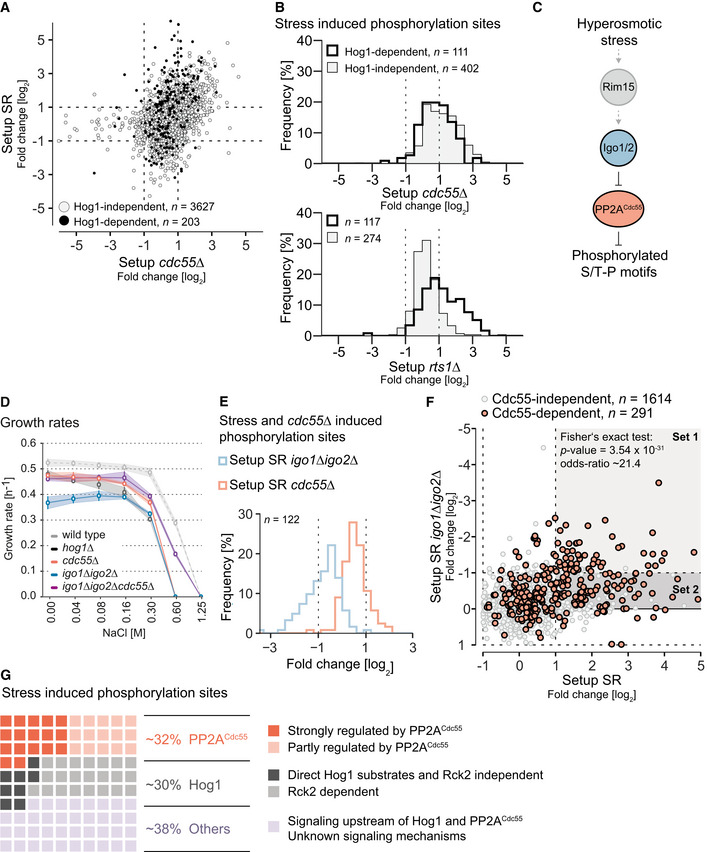
Deletion of Cdc55 affects phosphorylome at the onset of hyperosmotic stress AScatter plot displaying log2 SILAC ratios of individual phosphorylation sites in the experiment setup SR on the *y*‐axis and setup *cdc55Δ* on the *x*‐axis. Black dots represent Hog1‐dependent sites [ratio in setup SR ≥ 2 and affected by Hog1as inhibition (ratio ≤ 0.5)] (Romanov *et al*, 2017).BHistogram displaying the SILAC ratios (log2) of stress‐induced phosphorylation sites in setup *cdc55Δ* (upper panel) and setup *rts1Δ* (lower panel) (preprint: Hollenstein *et al*, 2020), stratified by their Hog1 dependence (gray area versus thick black line).CSchematic illustration of suggested model of Cdc55 regulation in the hyperosmotic stress response, with gray arrows signifying hypothetical relationships.DLine plot monitoring growth rate (*y*‐axis) of indicated strains (colored lines) under different osmolarity conditions (*x*‐axis).EHistogram displaying the SILAC ratios (log2) of stress‐induced phosphorylation sites that are induced in setup *cdc55Δ* in two separate experiments. The blue histogram shows the distribution of fold‐changes when *IGO1* and *IGO2* are deleted and cells are exposed to hyperosmotic stress (setup SR *igo1*∆*igo2*∆). The orange histogram shows the distribution of fold‐changes after deletion of *CDC55* and upon exposure to hyperosmotic stress (setup SR *cdc55*∆). The same set of sites was compared between the two experiments.FScatter plot displaying log2 SILAC ratios of phosphorylation sites in the experiment setup SR on the *x*‐axis and setup SR *igo1∆igo2∆* on the *y*‐axis. Phosphorylation sites that do not exhibit Cdc55 dependence are shown in gray, whereas Cdc55‐dependent phosphorylation sites are highlighted in orange. We found an enrichment for Cdc55‐dependent sites in the SR‐ and Igo1/Igo2‐dependent set (Set1) (Fisher’s exact test, *P*‐value = 3.54 × 10^−31^, odds ratio = 21.40).GWaffle plot showing the impact of different signaling mechanisms on the stress phosphoproteome. Each box corresponds to 1% of the entire set. Orange boxes represent the percentage of phosphosites regulated by PP2A^Cdc55^, whereas gray boxes represent phosphosites regulated by Hog1 and downstream of the MAPK. Light purple boxes indicate the phosphosites that are neither regulated by Hog1 nor PP2A^Cdc55^. Dependency was defined as log2 SILAC ratio below −1 or above 1. Rck2 dependency was extracted from Ref. (preprint: Hollenstein *et al*, 2020).

First, we examined whether hyperosmotic stress and loss of PP2A activity affect overlapping sets of phosphorylation sites. Indeed, 52.8 and 25.2% of all phosphorylation sites with an increased abundance upon hyperosmotic stress treatment also showed an increase in *cdc55∆* and *rts1∆* cells, respectively, indicating that several cellular functions might be commonly affected by hyperosmotic stress and PP2A activity (Fig 2A and 2B). Conversely, we could not detect an apparent connection between loss of PP2A and phosphorylation sites that were negatively affected by stress exposure. These results indicate that PP2A activity might oppose hyperosmotic stress signaling.

Next, we investigated whether PP2A^Cdc55^ and PP2A^Rts1^ affect similar or distinct parts of the stress‐dependent phosphorylome. We compared SILAC ratios of stress‐induced phosphorylation sites between setups *cdc55*∆ and *rts1*∆ (Fig EV1D). Of the 211 stress‐induced sites that showed Cdc55 or Rts1 dependency, only 34 (16%) were similarly affected by deletion of the individual regulatory subunits, suggesting different roles for the two PP2A complexes in response to high osmolarity conditions.

Despite its central role in hyperosmotic stress, the mitogen‐activated protein kinase (MAPK) Hog1 regulates only one third of the stress‐dependent phosphorylome (Romanov *et al*, 2017). Consequently, the remaining stress‐induced phosphorylation events are regulated by an unknown mechanism. We therefore tested whether and to what extent stress‐induced sites that are independent of Hog1 are regulated by either PP2A^Cdc55^ or PP2A^Rts1^. This set of sites was found to be solely affected by the deletion of *CDC55* and not *RTS1* (Fig 2A and B). In contrast, PP2A^Rts1^ targets a specific branch of the Hog1 signaling pathway, as we report in preprint: Hollenstein et al (2020). Thus, depending on the bound B‐subunit, PP2A seems to be involved in different signaling branches during the hyperosmotic stress response. Given that PP2A^Cdc55^ is known to play a role in regulating cell cycle progression (Godfrey *et al*, 2017; Moyano‐Rodriguez & Queralt, 2019; Touati *et al*, 2019), we tested whether deletion of *CDC55* and our experimental stress conditions could lead to a cell cycle arrest or delayed cell cycle progression, which would indirectly result in the observed changes to the phosphorylome. We used FACS to measure DNA content profiles of the wild‐type and knockout strains exposed to no stress, and 5 and 10 min of hyperosmotic stress. No significant differences in the distribution of cells could be identified (Fig EV1E). We also did not observe any particular abundance changes in well‐characterized cell cycle markers covered in our dataset (Fig EV1F) (Kelliher *et al*, 2018). These observations refute the possibility that the increase in phosphorylation in the *cdc55∆* strain can be attributed to cell cycle effects.

**Figure EV2:**
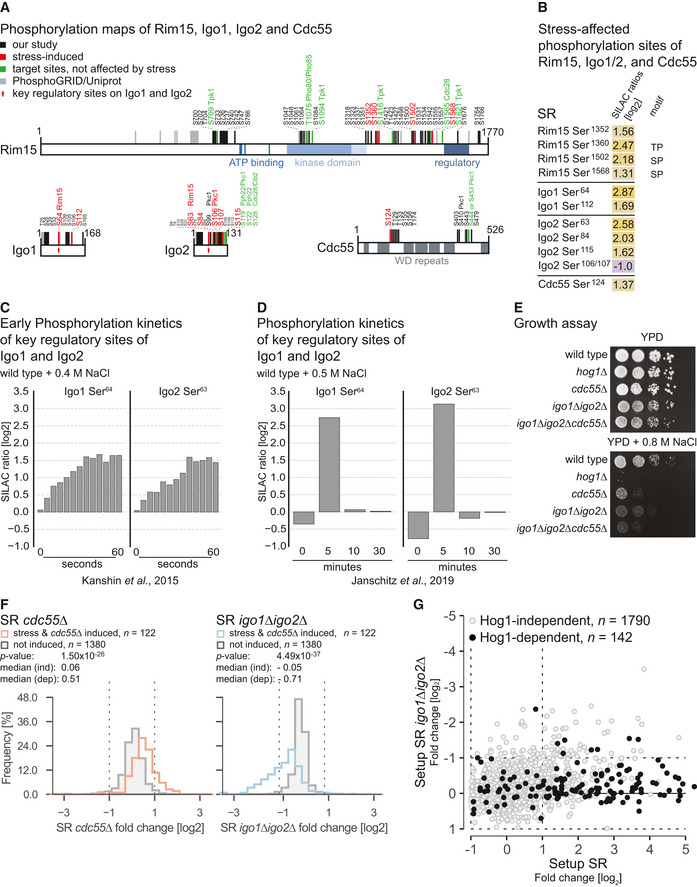
The Greatwall Kinase‐Endosulfin‐PP2A module plays a role in the hyperosmotic stress response (related to Fig 2) APhosphorylation site maps of Rim15, Igo1, Igo2, and Cdc55. Phosphorylation sites covered in our analysis are indicated in black (static in setup SR) or red (affected by osmostress). Phosphorylation sites listed in either PhosphoGRID (Sadowski *et al*, 2013) or Uniprot (UniProt Consortium, 2019) databases are indicated in gray. Green: target sites of indicated kinase or phosphatase activity (not covered in our analysis). Pkc1 sites were taken from (Thai *et al*, 2017).BTable presenting log2 SILAC ratios across sites of Rim15, Igo1 and Igo2, and Cdc55 that were affected by hyperosmotic stress treatment (abs(log2 fold change) ≥ 1).C, DExcerpt of published MS datasets showing SILAC log2 ratios of Igo1 and Igo2 key phosphorylation sites after hyperosmotic stress exposure: (C) within the first 60 s after treatment with 0.4 M NaCl (Kanshin *et al*, 2015); (D) at 0, 5, 15, and 30 min after treatment with 0.5 M NaCl (Janschitz *et al*, 2019).ESerial dilution spot assay monitoring growth of wild‐type, *cdc55∆*, *igo1∆igo2∆*, and *igo1∆igo2∆cdc55∆* cells under non‐stress conditions (upper panel) and in response to high osmolarity conditions (lower panel).FHistograms displaying the SILAC ratios (log2) of stress‐ and *cdc55Δ‐*induced phosphorylation sites in two separate experiments (orange and blue). The gray histograms only include phosphorylation sites that are neither affected by stress nor the absence of *CDC55*. Left histogram shows the distribution of fold‐changes after deletion of *CDC55* and upon exposure to hyperosmotic stress (setup SR *cdc55*∆). Right: Distribution of fold‐changes when *IGO1* and *IGO2* are deleted and cells are exposed to hyperosmotic stress (setup SR *igo1*∆*igo2*∆). The same set of sites was compared between the two experiments. *P*‐values were calculated using the Wilcoxon test. The indicated distributions are significantly different.GScatter plot displaying log2 SILAC ratios of phosphorylation sites in the experiment setup SR on the *x*‐axis and setup SR *igo1∆igo2∆* on the *y*‐axis. Phosphorylation sites that do not exhibit Hog1 dependence are shown in gray, whereas Hog1‐dependent phosphorylation sites are highlighted in black.

If PP2A^Cdc55^ activity counteracts hyperosmotic stress signaling, then stress exposure might cause inhibition of the phosphatase. The yeast Endosulfines Igo1 and Igo2 have been previously described as inhibitors of PP2A^Cdc55^ that become activated upon phosphorylation of a specific residue by the Greatwall kinase Rim15 (Juanes *et al*, 2013). Throughout the Rim15‐Igo1/Igo2‐Cdc55 regulatory module, we observed stress‐induced phosphorylation events (Fig EV2A and B) and substantial phosphorylation at the respective key regulatory residues of Igo1 and Igo2 (Igo1‐Ser^64^ 7.3× increase; Igo2‐Ser^63^ 6× increase, Fig 2C). Phosphorylation of these residues can already be observed within seconds after hyperosmotic stress exposure, indicating a very rapid upstream sensing mechanism (Fig EV2C, Kanshin *et al*, 2015). Moreover, stress‐induced phosphorylation of the Endosulfines appears to be transient and to return to almost basal levels after 15 min (Fig EV2D, Janschitz *et al*, 2019). Together these observations strongly point toward a typical stress signaling response that mediates PP2A^Cdc55^ inhibition in a very rapid and temporally regulated manner.

To test whether the Endosulfines are required for coping with hyperosmotic stress, we monitored the stress sensitivity of wild‐type and *igo1*∆*igo2*∆ cells in response to high osmolarity conditions using growth curves (Fig 2D). Cells lacking *IGO1/2* showed a moderate but clear susceptibility to increasing concentrations of NaCl, confirming that Igo1 and Igo2 are important for cellular survival under hyperosmotic stress conditions. Deleting *CDC55* also elicited a visible growth defect at concentrations above 0.3 M NaCl, but less pronounced than with the *IGO1/2* double deletion. The growth defect of *igo1∆igo2∆* cells could in turn be rescued by an additional deletion of *CDC55* (a triple knockout of *igo1∆igo2∆cdc55∆*) under non‐stressed as well as stress conditions. In fact, the growth rate of the triple knockout was almost equivalent to the growth rate of the *CDC55* single deletion, suggesting a full rescue in the monitored time period. In a similar experimental setup, we also looked at stress sensitivity to 0.8 M NaCl using a serial dilution spot assay (Fig EV2E). For both, *cdc55∆* and *igo1∆igo2∆*, we could confirm stress‐induced growth defects.

To confirm that inhibition of PP2A^Cdc55^ is directly involved in stress signaling, we performed SILAC‐MS experiments with cells exposed to hyperosmotic stress quantifying phosphorylation changes caused by the deletion of *IGO1/2* (setup SR *igo1∆igo2∆*) or deletion of *CDC55* (setup SR *cdc55*∆) (Fig 1). We observed that stress‐ and *cdc55* ‡∆^‡^‐induced phosphorylation sites showed a trend toward higher phosphorylation in setup SR *cdc55∆* (Figs 2E and EV2F). This was not the case in setup SR *igo1∆igo2∆*. In fact, respective phosphorylation sites displayed a significant decrease in abundance. Next, we analyzed the impact of *igo1*∆*igo2*∆ in more detail, focusing on sites that were induced in *cdc55∆* cells (291 sites) (Fig 2F). Nearly all *cdc55*∆‐ and stress‐induced phosphorylation sites (92%, 131 of 143 sites, 91 proteins) showed a negative fold change in setup SR *igo1∆igo2∆* (Fig 2E and Set 1 and Set 2 in Fig 2F). For some of those sites, the impact of *igo1∆igo2∆* was subtle, indicating that they are additionally targeted by stress‐activated kinases. However, we also observed 53 phosphorylation sites (corresponding to 40 proteins) that displayed strong dependency on Igo1 and Igo2 during exposure to hyperosmotic stress (Set 1, light gray area in Fig 2F). The inhibition of PP2A^Cdc55^ is thus the main driving force of increased phosphorylation at these sites upon hyperosmotic stress exposure. In contrast, loss of *IGO1* and *IGO2* had no major effect on the Hog1‐dependent stress phosphorylome (Fig EV2G). Our data thus demonstrate that one third of the stress‐induced phosphorylome is under control of PP2A^Cdc55^, making the phosphatase as impactful as Hog1 (Fig 2G). Taken together, we uncovered a phosphatase‐driven signaling mechanism, acting in parallel to and independent of a well‐established MAPK signaling pathway.

### PP2A^Cdc55^ directly targets a broad range of proteins with stress‐induced serine/threonine‐proline motifs

**Figure 3:**
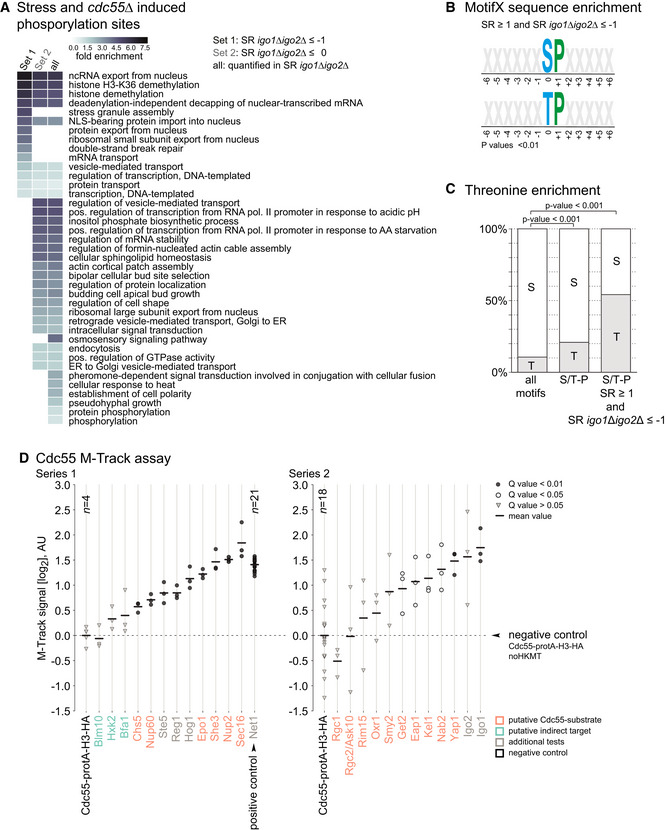
Analysis of proteins affected by PP2A^Cdc55^ and hyperosmotic stress AGene ontology enrichment of stress‐ and Cdc55‐dependent phosphorylation sites using DAVID v6.8 (Huang *et al*, 2009). Sets were defined according to phosphorylation site fold‐changes in setup SR *igo1∆igo2∆* (see Fig 2F). GO levels (of the category “GOTERM_BP_DIRECT”) were required to be between level 3 and level 6, and GO terms should have at least two counts in the given set. The heat map illustrates the respective fold enrichments for the resulting GO terms in each set. The heat map was sorted according to the enrichment values in Set1 and secondly according to the sum of the enrichment values in Set2 and all quantified phosphorylation sites in SR *igo1*∆*igo2*∆.BOverrepresented phosphorylation‐sequence motifs calculated with MotifX. Phosphorylation sites with a fold change ≥ 2 in setup SR and ≤ 0.5 in setup SR *igo1∆igo2∆* were compared to the whole set of phosphorylation sites quantified in setup SR and setup SR *igo1∆igo2∆* as background.CBar plots illustrating the phospho‐threonine enrichment of stress‐ and Igo1/Igo2‐dependent S/T‐P motifs. Phosphorylation sites quantified in both setup SR and setup SR *igo1∆igo2∆* were analyzed. Gray bars show the percentage of phospho‐threonine, white bars show the percentage of phospho‐serine. The enrichment was assessed using Fisher’s exact test.DResults of the M‐Track proximity assay. Cdc55 was fused to the protA‐H3‐HA prey‐tag, and putative substrate proteins were tagged with the HKMT bait‐tag. Background proximity signal was defined using a strain expressing only Cdc55‐protA‐H3‐HA and no tagged bait protein (*negative control*) and is shown as a dashed horizontal line. Western blot signals were quantified as described in the methods section and proximity signals are displayed as log2 fold over background. Dots/triangles show signals of individual replicates (*n* ≥ 3; if more than three replicates available, the sample size is indicated in the figure); horizontal bars indicate average proximity signals. Proximity signals that differ significantly from background are marked as black (*q* < 0.01) or white dots (*q* < 0.05), others as gray triangles (*q* > 0.05). AU, arbitrary units. Note that the differences in the variation and signal intensity between Series 1 and Series 2 are due to batch effects of the methylation‐specific antibody used (see Methods).

To identify cellular processes affected by stress‐induced PP2A^Cdc55^ inactivation, we performed a gene ontology (GO) analysis focusing on stress‐ and Cdc55‐dependent phosphorylation sites that are either strongly (Set 1) or moderately (Set 2) affected by deletion of *IGO1* and *IGO2* (Fig 3A). GO terms enriched in Set 1 were found to be mostly associated with processes regulating gene expression, such as regulation of transcription, mRNA transport, and histone demethylation. GO terms derived from Set 2 were associated with multiple processes that have been previously attributed to stress signaling (Kanshin *et al*, 2015; Romanov *et al*, 2017; Janschitz *et al*, 2019) including processes related to transcription, signal transduction, vesicular transport as part of endocytosis, retrograde transport machineries, and actin cortical patch assembly.

**Figure EV3:**
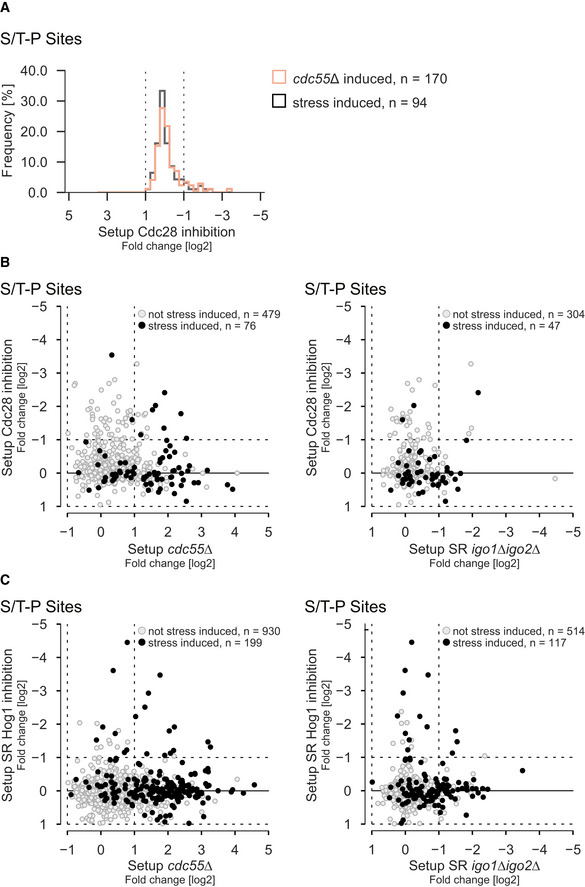
Stress‐ and Cdc55‐affected S/T‐P motifs are not targeted by Cdc28 or Hog1 (related to Fig 3) AHistogram displaying SILAC ratios (log2) from the Cdc28 inhibition dataset (Kanshin *et al*, 2017). The gray histogram includes only stress‐induced phosphorylation sites, and the orange histogram includes phosphorylation sites that are induced in setup *cdc55Δ*.BScatter plots comparing the Cdc28 inhibition dataset (Kanshin *et al*, 2017) with two other experiments. Left scatter plot displays log2 SILAC ratios of phosphorylation sites in the setup *cdc55Δ* on the x‐axis and the Cdc28 inhibition dataset on the y‐axis. Right scatter plot displays log2 SILAC ratios of phosphorylation sites in the setup SR *igo1∆igo2∆* on the x‐axis and the Cdc28 inhibition dataset on the y‐axis. Phosphorylation sites that do not exhibit stress dependence are shown in gray, whereas stress‐induced phosphorylation sites are highlighted in black.CScatter plots comparing the effect of Hog1 inhibition on the stress phosphorylome (setup SR Hog1 inhibition) with two other experiments. Left scatter plot displays log2 SILAC ratios of phosphorylation sites in the setup *cdc55Δ* on the x‐axis and the setup SR Hog1 inhibition on the y‐axis. Right scatter plot displays log2 SILAC ratios of phosphorylation sites in the setup SR *igo1∆igo2∆* on the x‐axis and the setup SR Hog1 inhibition on the y‐axis. Phosphorylation sites that do not exhibit stress dependence are shown in gray, whereas stress‐induced phosphorylation sites are highlighted in black.

PP2A^Cdc55^ preferentially dephosphorylates phospho‐threonines in threonine‐proline (TP) motifs (Agostinis *et al*, 1990; Cundell *et al*, 2016; Godfrey *et al*, 2017; Kruse *et al*, 2020). To examine whether the proteins involved in those biological processes might be directly dephosphorylated by PP2A^Cdc55^, we first performed a motif enrichment analysis using MotifX (Schwartz & Gygi, 2005). We found the serine‐proline (SP), threonine‐proline (TP) to be significantly enriched (FDR < 1%) (Fig 3B), with ˜60% of sites containing the motif. Moreover, the TP motif was significantly enriched within stress‐induced S/T‐P motifs affected by inhibition of PP2A^Cdc55^ (enrichment *P* < 0.001, Fig 3C). S/T‐P is the substrate motif of MAPKs and cyclin‐dependent kinases (CDK). PP2A^Cdc55^ counteracts global CDK phosphorylation in the context of cell cycle regulation and it has been suggested that PP2A^Cdc55^ directly dephosphorylates substrates of Cdc28—the homolog of mammalian Cdk1 and the main CDK driving the cell cycle in yeast (Mendenhall & Hodge, 1998; Godfrey *et al*, 2017). To investigate whether stress‐ and Cdc55‐dependent S/T‐P sites correspond to Cdc28 substrates, we integrated our phospho‐proteomic data with a published dataset analyzing the immediate impact of Cdc28 inhibition on the phosphoproteome (Kanshin *et al*, 2017) (Fig 1). The majority of S/T‐P sites induced by hyperosmotic stress or deletion of *CDC55* did not display any change in abundance upon inhibition of Cdc28 (Fig EV3A and B). In detail, of the 49 stress‐ and Cdc55‐dependent S/T‐P sites, only 7 (˜14%) displayed Cdc28 dependence. Vice versa, the deletion of *CDC55* also had no significant impact on S/T‐P sites affected by inhibition of Cdc28 (Fig EV3B). Similar results were obtained when comparing the setup SR *igo1∆igo2∆* with the Cdc28 inhibition data set (Fig EV3B). We also could confirm that the vast majority (> 90%) of stress‐ and Cdc55‐dependent S/T‐P sites are independent of the MAPK Hog1 (Fig EV3C). These data demonstrate that PP2A^Cdc55^ targets a unique set of mainly Cdk1 independent phosphorylation sites as part of the hyperosmotic stress response. Moreover, inhibition of PP2A^Cdc55^ appears to strongly regulate one third and affect more than half of all stress‐induced S/T‐P motifs, in contrast to Hog1, which targets about 11% of these motifs (Dataset EV1).

We next analyzed the ability of Cdc55 to interact with putative substrates *in vivo* using the M‐Track protein–protein proximity assay (Zuzuarregui *et al*, 2012; Romanov *et al*, 2017). Cdc55 was tagged with the M‐Track prey sequence consisting of the histone H3‐hemagglutinin (HA) tag fused to protein A (protA‐H3). Potential interactor proteins were fused to the enzymatic domain of the murine histone lysine methyltransferase SUV39 (HKMT‐myc) serving as bait. Upon close proximity of expressed bait‐ and prey‐protein, the protA‐H3 tag becomes permanently tri‐methylated on lysine 9 of histone H3 (me3K9H3). Out of 149 potential substrates of the phosphatase harboring *cdc55*∆^‡^‐ and stress‐induced S/T‐P phosphorylation sites, we selected a representative group of 16 proteins that appear in different cellular processes (Dataset EV1). Net1, the core subunit of the RENT complex, represents a well‐established substrate of PP2A^Cdc55^ and therefore served as a positive control (Zuzuarregui *et al*, 2012). We additionally included Igo1, Igo2, Ste5, the MAPK Hog1, and Reg1, a regulatory subunit of the protein phosphatase 1 complex, to probe for potential cross‐talk with different stress signaling networks. We also included three putative indirect targets (Bfa1, Blm10, and Hxk2) that contain phosphorylation sites affected by deletion of *CDC55* but lack stress‐induced S/T‐P motifs. Sixteen out of 25 tested bait proteins showed a significant proximity signal (*q*‐value < 0.05), including 11 out of the 16 putative substrate proteins containing Cdc55‐stress‐induced S/T‐P motifs (Fig 3D, Appendix Fig S2A and B). These results strongly suggest that PP2A^Cdc55^ not only regulates a substantial part of the stress‐induced phosphorylome but also directly targets a diverse set of stress‐associated proteins.

### Inhibition of PP2A^Cdc55^ by Igo1 and Igo2 is required for an efficient transcriptional response to hyperosmotic stress

**Figure 4:**
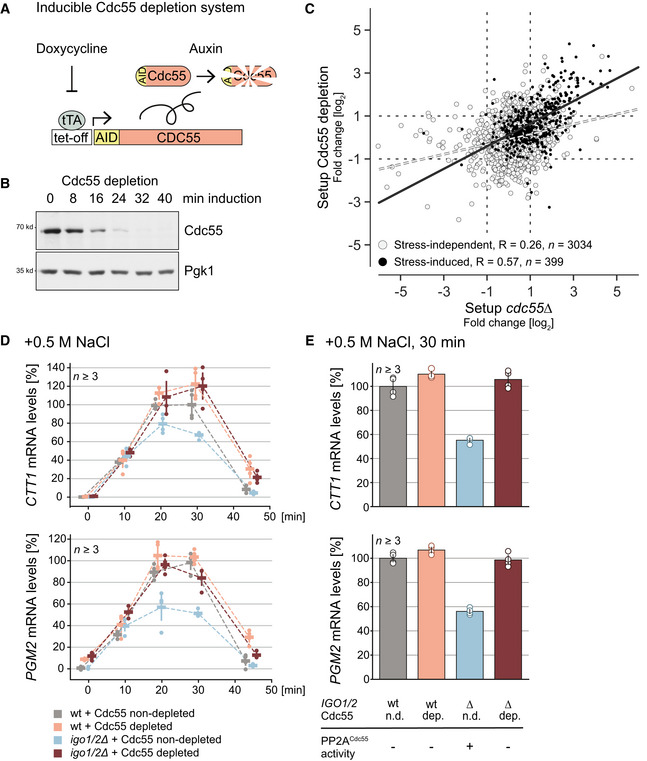
Inhibition of PP2A^Cdc55^ by Igo1/2 is required for an efficient transcriptional response to hyperosmotic stress ASchematic illustration of iAID‐conditioned Cdc55 depletion system: Cdc55 is fused with an AID (Auxin‐inducible degron) tag rendering the protein sensitive to auxin (Nishimura *et al*, 2009; Morawska & Ulrich, 2013). Expression of AID‐Cdc55 is controlled by a Tet^Off^ promoter and is repressed in the presence of doxycycline. Upon addition of auxin Cdc55 is rapidly depleted.BWestern blot illustrating the depletion of AID‐Cdc55 at various time points after addition of auxin (upper panel); Pgk2 serves as a negative control (lower panel).CScatter plot of log2 fold‐changes of overlapping phosphorylation sites from setup *cdc55Δ* (*x*‐axis) and the Cdc55 depletion system (*y*‐axis). Stress‐independent sites are shown in gray; stress‐induced sites are shown in black. The dotted lines mark twofold thresholds.DTranscriptional analysis of stress‐induced expression of ESR genes *CTT1* (upper panel) and *PGM2* (lower panel) under different strain conditions (i.e., presence/absence of *IGO1/2* and induction of iAID‐Cdc55 depletion). Measured time points after stress application are depicted on the *x*‐axis, target gene mRNA levels are illustrated on the *y*‐axis as fold over reference gene *IPP1*, and values are normalized relative to wild type without induction of iAID‐Cdc55 depletion at 30 min post‐stress.ETranscriptional analysis of stress‐induced expression of ESR genes *CTT1* (upper panel) and *PGM2* (lower panel) at 30 min post‐stress. Target gene mRNA levels are illustrated on the *y*‐axis as fold over reference gene *IPP1*; values are normalized relative to wild type without induction of iAID‐Cdc55 depletion. dep.: Cdc55 depleted, n.d.: Cdc55 non‐depleted.

**Figure EV4:**
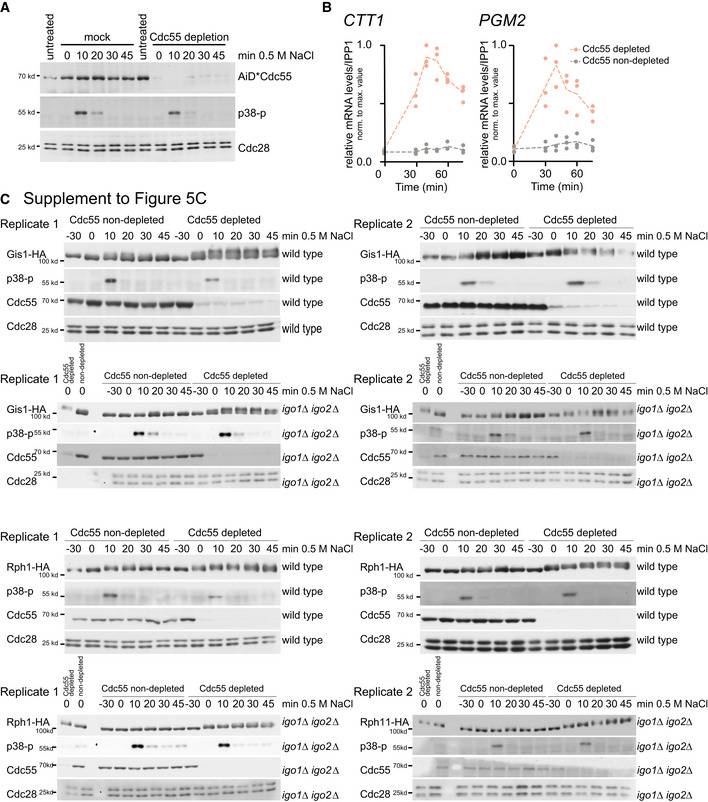
Depletion of Cdc55 affects Gis1 and Rph1 phosphorylation patterns (related to Figs 4 and 5) AInducible Cdc55 depletion system shows effective degradation of Cdc55, which is not affected by hyperosmotic stress treatment. Cdc55‐iAID‐expressing cells were mock‐treated (left) or treated with auxin and doxycycline (right) for 30 min followed by exposure to 0.5 M NaCl for times indicated.BCdc55 degradation increases *CTT1* and *PGM2* expression in absence of stress. Relative expression levels (fold over *IPP1*) of *CTT1* (left panel) and *PGM2* (right panel) in mock treated (gray dots) and auxin and doxycycline treated (orange dots) in Cdc55‐iAID cells. Values are normalized relative to the highest value.CMobility shift assays (see Fig 5C) monitoring phosphorylation‐induced mobility changes of Gis1 and Rph1 upon hyperosmotic stress in wild‐type, *igo1*∆*igo2*∆‐, and Cdc55‐depleted cells. Two biological replicates are shown. Hyperosmotic stress treatment was controlled using an antibody directed against dual‐phosphorylation of MAPK Hog1 (p38‐p); Cdc55 protein levels were controlled using an anti‐Cdc55 antibody. Cdc28: loading control.

We next focused on the role of PP2A^Cdc55^ in the transcriptional regulation in response to hyperosmotic stress. Previously we observed a reduced transcriptional response to hyperosmotic stress in *cdc55∆* cells (Reiter *et al*, 2013), which is in contrast to the finding that inhibition of PP2A^Cdc55^ is part of the stress response. To avoid possible adaptation effects caused by permanent loss of PP2A^Cdc55^ activity, we utilized the iAID (improved Auxin‐inducible degron) (Tanaka *et al*, 2015) system to set up a conditional Cdc55 depletion system (Fig 4A). Cdc55 was readily and efficiently depleted within 30 min after induction (Fig 4B) without having any visible impact on the activation loop phosphorylation and hence activity of Hog1 upon hyperosmotic stress (Fig EV4A). Using the Cdc55‐iAID strain, we performed SILAC‐MS experiments to capture the immediate effect of Cdc55 depletion on the phosphoproteome. Similar to setup *cdc55∆*, we found increased phosphorylation for several known hallmarks (Gis1, Net1, Ndd1, Sic1, Sli15, and Swe1) (Dataset EV1). In general, transient Cdc55 depletion appears to have a similar impact on the global phosphoproteome as a deletion of *CDC55*, although discrepancies between the two experimental conditions were indeed apparent (Fig 4C). Importantly, when focusing on stress‐induced phosphorylation sites, we observed an agreement of *R* ˜ 0.57 between the two datasets, which is relatively high given that the steady state of a constitutive knockout is compared to a dynamic depletion after 30 min (Fig 4C). Notably, the depletion of Cdc55 did not elicit any significant cell cycle effects under the experimental conditions used (Fig EV1E). These results demonstrate that Cdc55 depletion effectively recapitulates the loss of PP2A^Cdc55^ phosphatase activity while avoiding alterations in cellular physiology that might be caused by persistent loss of *CDC55*.

To test the direct effect of PP2A^Cdc55^ deprivation on transcription, we monitored the expression of two paradigm environmental stress response (ESR) genes, *CTT1* (Marchler *et al*, 1993) and *PGM2* (François & Parrou, 2001) in the Cdc55 depletion system. These ESR genes are typical and representative readouts for the transcriptional response upon stress induction due to their high dynamic range (Belazzi *et al*, 1991; Rep *et al*, 1999; Gasch *et al*, 2000; Klopf *et al*, 2009; Reiter *et al*, 2013). We first recorded stress‐induced transcriptional profiles for effects of Cdc55 depletion and Igo1/Igo2 deletion (Fig 4D) and observed the biggest effect of the knockouts on mRNA levels at 30 min after induction. We therefore re‐measured this particular time point in a separate series of experiments (Fig 4E). Cdc55 depletion elicited no decrease but even a slightly elevated transcriptional response of both genes. In contrast, when *IGO1* and *IGO2* were deleted, we observed a strongly reduced induction of mRNA levels (Fig 4E). Cdc55 depletion rescued stress‐induced transcription of *CTT1* and *PGM2* in *igo1∆igo2∆* cells, resembling wild‐type levels. Interestingly, Cdc55 depletion without stress induces a transient minor boost in transcription (˜10‐ and 5‐fold induction of *CTT1* and *PGM2*, respectively, compared to a 700‐ and 40‐fold induction observed after 30 min 0.5 M NaCl treatment) (Fig EV4B). These results demonstrate that PP2A^Cdc55^ activity negatively affects stress‐induced transcription and that inhibition of the phosphatase is required to elicit a strong transcriptional response.

### PP2A^Cdc55^ signaling directly regulates the transcriptional activity of Gis1 and Rph1

**Figure 5:**
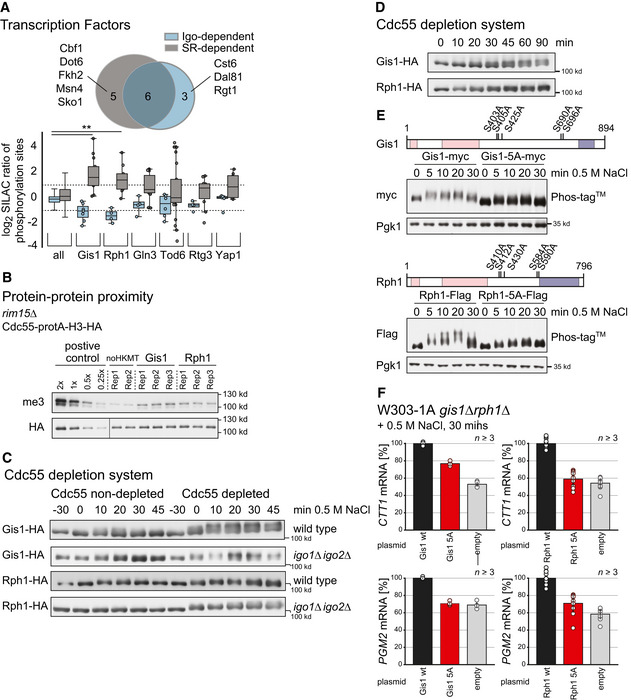
Transcriptional activity of Gis1 and Rph1 is activated by inhibition of PP2A^Cdc55^ AVenn diagram of 14 transcription factors [as recovered from the YEASTRACT database (Teixeira *et al*, 2006, 2018)] with at least one phosphorylation site changing twofold in setup SR (gray) or SR *igo1∆igo2∆* (blue). For the six overlapping transcription factors, the log2 SILAC ratios of phosphorylation sites are shown (*y*‐axis) in the corresponding setups (color code as above). The first two box plots illustrate the distribution of log2 SILAC ratios of all phosphorylation sites quantified in each setup (‘all’). Gis1 and Rph1 were found to be significantly different in their distributions (*P* < 0.01, *t*‐test relative to “all”).BM‐Track proximity assay of Cdc55 fused to the protA‐H3‐HA prey‐tag and Gis1, respectively, Rph1, tagged with the HKMT bait‐tag expressed in a *rim15∆* strain background. Background signal intensity was defined using a strain expressing only Cdc55‐protA‐H3‐HA and no tagged bait protein (*noHKMT*). Positive control: Nup2‐HKMT, CDC55‐protA‐H3HA. me3: antibody recognizing me3K9H3; HA: 12CA5 antibody.CMobility shift assays monitoring phosphorylation‐induced mobility changes of Gis1 (two upper rows) and Rph1 (two lower rows) upon hyperosmotic stress in wild‐type, *igo1∆igo2∆‐*, and Cdc55‐depleted cells (right‐hand side).DMobility shift assay of Gis1 and Rph1 across several time points after Cdc55 depletion (without exposure to hyperosmotic stress). Cdc28: loading control (shown in Fig EV5C).EWestern blot phosphorylation shift assays of wild‐type Gis1‐myc (upper panel) and Rph1‐Flag (lower panel), and mutated forms Gis1‐5A‐myc (upper panel) and Rph1‐5A‐Flag (lower panel). The measurement was done using Phos‐tag (Kinoshita *et al*, 2006) and at the indicated time points after exposure to hyperosmotic stress (0.5 M NaCl) using Pgk1 protein levels as a negative control. The location of the mutated residues is shown in the respective schematics. Red boxes: demethylase associated domains (JmjN and JmjC), blue box: zinc finger domain.FTranscriptional analysis of stress‐induced expression of ESR genes *CTT1* (upper panel) and *PGM2* (lower panel) at 30 min post‐stress across indicated strains expressing either wild‐type or point‐mutated forms of Gis1 and Rph1 in a *gis1*∆*rph1*∆ deletion background, with the empty vector as control.

To understand how PP2A^Cdc55^ regulates transcription in detail, we screened setups SR and SR *igo1∆igo2∆* for transcriptional regulators with altered phosphorylation patterns (Fig 5A). The paralogous demethylase domain‐containing transcriptional regulators, Gis1 and Rph1, stood out as 52–60% of their covered phosphorylation sites were found to be affected under both experimental conditions (*P* < 0.01, *t*‐test relative to background). Phosphorylation of Gis1 and Rph1 has been reported to play an important role in the regulation of their transcriptional activity (Liang *et al*, 2013). In addition, Gis1 has been biochemically shown to interact with Cdc55 (Bontron *et al*, 2013).

To test for *in vivo* interaction of PP2A^Cdc55^ with Gis1 and Rph1, we performed M‐track assays. We used *rim15*Δ cells to prevent activation of Igo1 and Igo2 and thereby increase PP2A^Cdc55^ activity, and expressed Cdc55 as prey and the transcription factors as bait proteins. Gis1 and Rph1 showed proximity signals above background control levels (Fig 5B), suggesting that the active phosphatase directly interacts with Gis1 and Rph1.

**Figure EV5:**
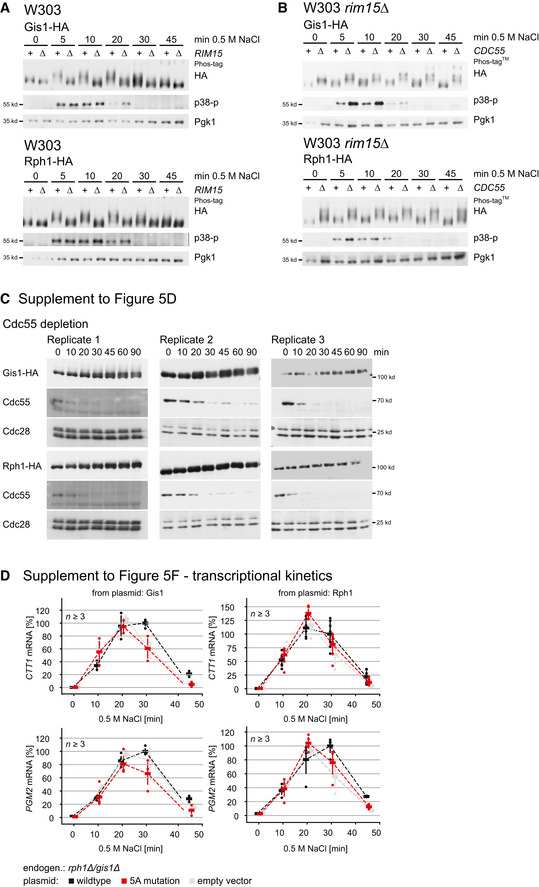
Reduced transcriptional activity of Gis1‐5A and Rph1‐5A mutants in response to stress (related to Fig 5C and D) Mobility shift assay of Gis1 and Rph1 (see Fig 5D) across several time points after Cdc55 depletion without exposure to hyperosmotic stress. Three biological replicates are shown. Cdc55 protein levels were controlled using an anti‐Cdc55 antibody. Cdc28: loading control.Rim15 affects Gis1 and Rph1 phosphorylation patterns during hyperosmotic stress. Wild‐type (+) or *rim15*Δ (Δ) cells expressing *GIS1*‐HA (upper panel) or *RPH1*‐HA (lower panel) were treated with 0.5 M NaCl for times indicated. Gel mobility shifts were visualized via Phos‐tag gels (Phos‐tagTM) using a 12CA5 antibody (HA). Activation of the high osmolarity glycerol (HOG) pathway (sign of effective salt stress treatment) was controlled with an anti‐phospho‐p38 antibody recognizing dual‐phosphorylated Hog1. Pkg1: loading control.Rim15 indirectly affects phosphorylation of Gis1 and Rph1 via a Cdc55‐dependent mechanism. *rim15*Δ (+) and *rim15*Δ*cdc55*Δ (Δ) cells expressing *GIS1*‐HA (upper panel) or *RPH1*‐HA (lower panel) were treated with 0.5 M NaCl for times indicated. Western blot experiments were similar as described in (B).Transcriptional kinetics of stress‐induced expression of ESR genes *CTT1* (upper panel) and *PGM2* (lower panel) across indicated strains expressing point‐mutated forms of Gis1 and Rph1 (related to 5F).

To address whether PP2A regulates stress‐induced phosphorylation of Gis1 and Rph1, we examined phosphorylation‐induced mobility shifts upon hyperosmotic stress in a time‐resolved manner in wild‐type, *igo1*∆*igo2*∆‐, and Cdc55‐depleted cells (Figs 5C and EV4C). In the wild type, both proteins showed a transient phosphorylation‐induced upshift, peaking at around 20 min after stress induction and returning to the basal state within 45 min. When Cdc55 was depleted prior to stress induction, Gis1‐HA as well as Rph1‐HA exhibited increased upshifts and remained so throughout the monitored time period, indicating that PP2A^Cdc55^ activity is dispensable for induction of phosphorylation but required for dephosphorylation of the proteins after termination of the stress response. Deletion of the PP2A^Cdc55^ inhibitors *IGO1* and *IGO2* almost completely abolished stress‐induced upshifts of Gis1 and Rph1; however, additional depletion of Cdc55 was able to fully restore the phosphorylation shifts (Fig 5C). We made similar observations for *rim15*∆ cells, which is consistent with Rim15 being the upstream activator of Igo1 and Igo2 (Fig EV5A and B). These results demonstrate that PP2A^Cdc55^ needs to be inactivated to allow stress‐induced phosphorylation of Gis1 and Rph1.

We next tested whether Cdc55 depletion was able to induce mobility shifts in the absence of hyperosmotic stress. Indeed, depletion of Cdc55 was sufficient to induce a rapid and time‐dependent increase of Gis1 and Rph1 phosphorylation under non‐stress conditions (Figs 5D and EV5C). Together with the observation that Cdc55 directly interacts with Gis1 and Rph1, this strongly suggests that inhibition of PP2A^Cdc55^ in response to hyperosmotic stress shifts the balance of basal kinase and phosphatase activity affecting on Gis1 and Rph1 toward increased phosphorylation, without requiring the concurrent activation of a kinase.

Consequently, we expected alanine substitutions of stress‐ and Cdc55‐dependent phosphorylation sites of Gis1 and Rph1 to prevent their activation and result in a reduced transcriptional response upon hyperosmotic conditions. We therefore aimed for a set of point mutations in the transcription factors to selectively inactivate stress signaling mediated by PP2A^Cdc55^. To deconvolute which phosphorylation sites of the two transcription factors are affected by PP2A^Cdc55^ as well as hyperosmotic stress, we captured changes in the phosphorylation pattern of Gis1 and Rph1 upon 15, 30, and 60 min depletion of Cdc55 using SILAC‐MS and integrated this dataset with our phospho‐proteomics datasets (Dataset EV2). Thereby, we generated detailed phospho‐maps for Gis1 and Rph1, highlighting which sites are affected by PP2A^Cdc55^ inhibition during the stress response. We selected a total of five S/T‐P sites that were conserved between the two paralogues and displayed stoichiometric phosphorylation upon depletion of Cdc55, to be mutated to alanine (Fig 5E). Using Western blot phosphorylation shift assays, we confirmed that both point‐mutated proteins become less phosphorylated upon stress exposure (Fig 5E). The remaining marginal upshift of Rph1 and Gis1 indicates that our mutations did not cover all stress‐induced sites, which was expected, since both proteins contain a multitude of non‐mutated phosphorylation sites.

We next tested the effect of the alanine substitutions on the transcriptional response, using a *gis1∆rph1∆* strain expressing the respective wild‐type or point‐mutated version of one of the proteins from plasmid. We recorded the transcriptional profiles of *CTT1* and *PGM2* during a period of 45 min exposure to hyperosmotic stress (Fig EV5D). While the initial increase of mRNA levels was not affected by the absence of both transcription factors, we observed a premature decline after 30 min post‐stress exposure. These results suggest that Gis1 and Rph1 do not function during the early induction of the transcriptional stress response but rather support a high transcriptional output over an extended period of time. Since we observed the strongest effect after 30 min exposure to hyperosmotic stress, we re‐measured this time point in a separate experiment and observed the mRNA levels of *CTT1* and *PGM2* decrease by 50–100% for the 5A mutant alleles relative to the respective knock out (Fig 5F). We therefore propose that direct dephosphorylation by PP2A^Cdc55^ suppresses the transcriptional activity of Gis1 and Rph1 and that hyperosmotic stress‐mediated inhibition of the phosphatase is necessary to allow phosphorylation‐induced activation of these transcription factors.

In summary, our results portray PP2A^Cdc55^ as a central downstream effector of a phosphatase‐centric signaling pathway, which causes major phospho‐proteomic changes and thereby enables cellular adaptation in response to extracellular stimulation.

## Discussion

Reversible protein phosphorylation is widely studied to understand cellular behavior; however, the regulatory underpinning of phospho‐proteomic adaptations is still largely unknown. Historically, research to understand the phosphorylome has mainly focused on kinase signaling pathways, while the possible active contribution of phosphatases has not received similar attention. Here, we propose a signaling principle, in which the inhibition of a phosphatase is the key event resulting in increased phosphorylation, while induction of kinases affecting the same substrates appears not to be required.

**Figure 6:**
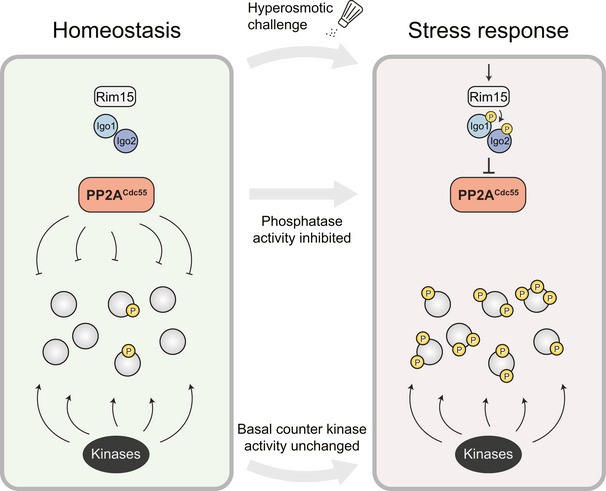
PP2A^Cdc55^ pathway The phosphatase PP2A^Cdc55^ lies at the center of a hyperosmotic stress signaling cascade. Under non‐stress conditions (left), the balanced activities of PP2A^Cdc55^ and counteracting kinase(s) result in a steady state of low phosphorylation of shared substrates. Hyperosmotic stress signaling transmitted through the Greatwall kinase/Endosulfin module activates the PP2A^Cdc55^ inhibitors Igo1 and Igo2 via phosphorylation at key regulatory sites (right). The resulting inhibition of the phosphatase is essential to tip the balance toward increased net phosphorylation.

PP2A^Cdc55^ stands at the core of a signaling pathway that is required and sufficient to induce phosphorylation of a wide range of proteins as part of the hyperosmotic stress response in budding yeast. The majority of the PP2A‐affected phosphorylation sites correspond to S/T‐P motifs. Absence of the phosphatase activity is required for the stress‐induced phosphorylation of these sites. Moreover, depletion of Cdc55 under unstressed conditions is sufficient to induce a similar phosphorylation response. Thus, regulation of PP2A activity constitutes the main driving force behind these particular phosphorylation changes. From a classical kinase‐centric point of view, one expects PP2A^Cdc55^ to control a downstream kinase that mediates those phosphorylations, rather than directly acting on a broad set of substrates (Chan & Amon, 2009; Park *et al*, 2012; Khanna *et al*, 2013; Wandzioch *et al*, 2014; Courtney & Deiters, 2019). S/T‐P motifs, for that matter, are targeted by CDKs and MAPKs. It is also well known that PP2A^Cdc55^ antagonizes Cdk1 phosphorylation during the cell cycle (Mochida *et al*, 2009; Godfrey *et al*, 2017; Touati *et al*, 2019). However, we find that inhibition of Cdc28 does not affect stress‐induced S/T‐P phosphorylation sites and that PP2A^Cdc55^ acts on a complementary set of S/T‐P sites during hyperosmotic stress (Fig EV3A and B). Interestingly, the CDK Pho85 has been described to mediate hyperosmotic stress signaling, but appears to be located upstream of Rim15, and therefore also of PP2A^Cdc55^ (Wanke *et al*, 2005; Jin *et al*, 2017). The other CDKs in yeast have been primarily connected to transcription‐related phosphorylation events (Nishizawa *et al*, 2010) and would therefore not account for the majority of targeted substrates. There is also evidence that none of the five proline‐directed MAP kinases in *S. cerevisiae* could act as the hypothetical stress‐activated counter‐kinase downstream of PP2A^Cdc55^. The MAPK Smk1 is not expressed in haploid cells, and the MAPKs Slt2 and Fus3 do not become activated during the immediate stress response (Pierce *et al*, 1998; García‐Rodríguez *et al*, 2005; Hao *et al*, 2008). We found over 90% of stress‐ and PP2A^Cdc55^‐dependent S/T‐P motifs to be independent of Hog1, while deletion of the MAPK Kss1 has been described to not affect Hog1‐independent, stress‐induced S/T‐P sites (Romanov *et al*, 2017). Thus, none of the proline‐directed kinases appear to act downstream of PP2A^Cdc55^. Moreover, our experiments imply that the phosphatase directly targets these S/T‐P phosphorylation sites, pointing toward a phosphatase‐centric signaling mechanism as depicted in Fig 6. Based on our results, we propose the following model: Under unstressed conditions, the phosphatase and counteracting kinase activities are balanced, resulting in a steady state of low phosphorylation of shared substrates. Upon hyperosmotic stress, the phosphatase becomes inhibited, tipping the balance toward increased net phosphorylation, rendering it a key event in regulating a global signaling response. In fact, our detailed analysis of the phosphorylation behavior of the Cdc55 substrates Gis1 and Rph1 further substantiates the proposed model. In an alternative scenario, PP2A^Cdc55^ could inhibit the activity of one or several unknown kinases under non‐stress conditions that share common S/T‐P substrate sites with the phosphatase. Inhibition of PP2A^Cdc55^ would result in activation of these kinases and therefore an increase in phosphorylation due to the combinatory effects of phosphatase inhibition and simultaneous kinase activation. However, as described in detail before, there is no evidence that the shift in the balance of substrate phosphorylation is associated with the activation of any known proline‐directed kinase. Further studies will undoubtedly be required though to conclusively prove that no concurrent kinase activation takes place upon PP2A^Cdc55^ inhibition. Stress‐induced inhibition of PP2A^Cdc55^ works through the Greatwall Kinase‐Endosulfine module, constituting a conserved three‐tiered signaling pathway with the phosphatase as the core element (Mochida & Hunt, 2012; Castro & Lorca, 2018). This pathway has been described to function in the context of cell cycle regulation and nutrient starvation, where it assists the increased activity of a kinase by inhibition of the counteracting phosphatase PP2A^Cdc55^ (Juanes *et al*, 2013; Sarkar *et al*, 2014), but never as a stand‐alone effector system without simultaneous kinase activation. Inhibition of PP2A^Cdc55^, as the main downstream signaling effector, causes major phosphorylation changes upon hyperosmotic stress to elicit a transcriptional response allowing for cellular adaptation. The signaling is terminated once the phosphatase becomes activated again, restoring the pre‐stressed state of the phosphorylome (Williams *et al*, 2014). The discovery of this phosphatase‐centric signaling mechanism is surprising since it represents the exact opposite to the traditional view of the rather passive role of phosphatases as antagonists of kinase‐mediated signaling.

The mammalian version of PP2A plays a key role in the pathogenesis of various human diseases, ranging from cancer and neurodegenerative diseases to immunoregulatory dysfunctions (Perrotti & Neviani, 2008; Sontag & Sontag, 2014; Apostolidis *et al*, 2016; Meeusen & Janssens, 2018; Westermarck, 2018; Bao *et al*, 2020). It is due to PP2A’s systemic impact on cellular processes that it is highly relevant to precisely understand its action and regulation (Westermarck, 2018). Given that all elements of the Greatwall Kinase‐Endosulfine‐PP2A‐B55 pathway are highly conserved, it is not unlikely that a similar PP2A‐B55‐driven signaling response exists in mammalian cells. Looking at PP2A as an active and broadly acting downstream signaling effector in addition to its role as an antagonist of kinases could therefore potentially assist approaches in tackling human diseases (Álvarez‐Fernández *et al*, 2018; Kauko *et al*, 2018; McClinch *et al*, 2018; O’Connor *et al*, 2018; Mazhar *et al*, 2019).

Our description of such a phosphatase‐driven signaling response leads to the question as to whether this mode of action applies to other phosphatases. There are two other prominent phosphatase‐centric pathways where the phosphatase represents a regulated effector element that directly targets a broad set of substrates, PP2B and PP1. PP2B has been mainly described to become activated by increased intracellular Ca^2+^ levels, which results in dephosphorylation of PP2B substrates (Park *et al*, 2019; Creamer, 2020). This is distinctly different from our proposed model, where the basal activity of the phosphatase becomes inhibited causing the exact opposite impact on the phosphorylome. PKA signaling, on the other hand, seems to follow a similar architecture as we describe here with the inhibition of PP1 leading to increased phosphorylation for example in neurons (Leslie & Nairn, 2019). This seems to primarily affect PKA substrates, which appear to require both activation of PKA and inhibition of PP1. It is likely that inhibition of PP1 could be sufficient to induce phosphorylation of non‐PKA substrates as well and thus could represent a similar modus operandi as we describe for PP2A^Cdc55^ in the context of stress signaling. Such mechanistic details and their systematic dissection still remain to be explored for many phosphatases. One of the reasons that could complicate such studies might lie in the difficulty of tracking phosphatase activity and understanding the underlying complex regulatory mechanisms (Shi, 2009; Fahs *et al*, 2016). In recent years, different approaches have emerged for selective phosphatase inhibition, ranging from targeted protein degradation, inducible CRISPR tools to chemical inhibitor compounds (Köhn, 2020). These technological developments, along with the increasing throughput and accessibility to MS‐based proteomics technologies, are the perfect platform to investigate the existence of other potential phosphatase‐driven signaling mechanisms.

Another important factor that might have hampered the discovery of such signaling mechanisms is the natural assumption that an induction of phosphorylation upon a signal is ultimately caused by the activation of a kinase rather than the inhibition of a phosphatase. However, the regulation of positive phosphorylation in signaling responses is only partly explained by kinase signaling so far. As such, the concept of phosphatase‐driven signaling mechanisms could provide an alternative explanation of how these phospho‐proteomic‐adaptations are achieved. Considering that in our study as much as 30% of stress‐induced phosphorylation is regulated by PP2A^Cdc55^, the impact of such phosphatase signaling could be quite significant. The extent to which phosphorylation can be fine‐tuned would also be severely increased due to potential synergistic interaction between phosphatase and kinase pathways on shared substrates (García‐Blanco *et al*, 2019). We therefore argue that our unexpected findings of phosphatase inhibition as a mechanism to induce a positive phosphorylation response could be pivotal for a holistic understanding of cellular signaling.

## Materials and Methods

### Yeast strains and plasmids

*Saccharomyces cerevisiae* strains used in this study are listed in Table EV2 (Thomas & Rothstein, 1989). Strains WR365 (W303‐1A SILAC, *cdc55*Δ, Mat a) and WR366 (W303‐1A SILAC, *cdc55*Δ, Mat α) were obtained by transforming WR209 (W303‐1A SILAC, Mat a) and WR210 (W303‐1A SILAC, Mat α) (Reiter *et al*, 2012) with an *CDC55* deletion cassette, amplified by PCR using corresponding S3 and S4 primers and plasmid pGA2260/pFA6a‐*HIS3MX6* as template (Wach *et al*, 1994). Strain WR1739 (W303‐1A SILAC, *igo1*Δ*igo1*Δ, Mat a) was obtained by transforming WR209 with respective deletion cassettes amplified from plasmids pFA6a‐*hphMX6* and pFA6a‐*natMX6* (Hentges *et al*, 2005). Other deletion mutant strains were generated as follows: W303‐1A was transformed with PCR amplifications of the corresponding deletion cassettes, using plasmids pGA2260/pFA6a‐*HIS3MX6* (Wach *et al*, 1994), pGA2264/pFA6a‐*TRP1MX6* (Wach *et al*, 1994), pGA2259/pFA6a‐*kanMX6* (Wach *et al*, 1994), pFA6a‐*hphMX6* (Hentges *et al*, 2005), or pFA6a‐*natMX6* (Hentges *et al*, 2005) as templates and primers designed as described above, giving rise to yeast strains GG589, GG593, GG594, JF83, JV1028. Construction of iAID strains involved multiple cloning steps: First, a n‐terminal AID*‐tagging cassette for *CDC55* was constructed by subcloning the AID* DNA sequence (PCR‐amplified from p2352 (Morawska & Ulrich, 2013) into YIPlac211 and subsequent insertion of the tetO7‐CYCmin promoter sequence and a 654bp n‐terminal fragment of *CDC55* (in frame), resulting in plasmid pGG95 (YIPlac211‐tetO7‐CYCmin‐AID*‐N‐*CDC55*). Yeast strains W303‐1A and WR209 were transformed with restriction cut pL235 (PTA4998^TM^; ATCC) for integration of *Oryza sativa* TIR1 (OsTIR1) gene into the *HIS3* locus. Positive transformants were transformed with PCR products encoding the tetO7‐CYCmin‐AID*‐N‐*CDC55* cassette amplified from pGG95. tTA (tetR‐VP16) was subcloned into pL253 (replacing OsTIR1), resulting in a *GPD1‐*promoter‐tTA fusion that was introduced into YIPlac128, giving rise to pGG92 (YIPlac128‐pGPD1‐tTA). The tTA‐containing fragment was amplified by PCR and integrated into the *LEU2* ORF, resulting in yeast strains GG163 and GG292. Deletion mutant derivatives of GG163 (Table EV2) were constructed as described above. Endogenous tagging of *GIS1* and *RPH1* with hemagglutinin (HA)‐tags (yeast strains GG267, GG274, GG590, GG592, GG597, and GG600) was obtained by transformation of W303‐1A and GG163 with HA‐tagging cassettes PCR‐amplified from plasmid pYM3/pGA2255/pFA6a‐6HA‐*kanMX6* (Knop *et al*, 1999). Endogenous tagging of *GIS1* and *RPH1* with HTBeaq (Reiter *et al*, 2012) was achieved by transformation of GG292 with HB‐tagging cassettes amplified from plasmids pWR268 and pWR160 (Reiter *et al*, 2012) resulting in yeast strain GG573. Yeast strains used in M‐track assays were generated as follows: WR974 (S288c *CDC55*‐TEV‐Protein A‐Histone3‐HA, Mat a) was obtained by transforming a S288c *CDC55*‐GFP strain (WR968) of the yeast strain‐library available from Life Technologies (http://clones.lifetechnologies.com; Huh *et al*, 2003) with PacI/SacI cut plasmid pCK902, encoding the TEV‐ProteinA‐Histone3‐HA cassette (Brezovich *et al*, 2015). WR1030 (S288c *CDC55*‐TEV‐ProteinA‐Histone3‐HA, Mat α) was obtained from backcrossing WR1242 with a S288c wild‐type, Mat α strain. M‐track strains WR1111–WR1771 were obtained by transformation of corresponding GFP library strains (Huh *et al*, 2003) with PacI/PmeI restriction digests of plasmid pCK900, encoding the myc‐HKMT tagging cassette. Positively tested transformants were crossed with WR1030 resulting in the final M‐track strains. M‐track strains MJ334 to MJ336 were obtained by transformation of WR974 with PCR amplifications of the myc‐HKMT tagging cassette (Brezovich *et al*, 2015). For PCRs, plasmid pJA31 (Janschitz *et al*, 2019) and corresponding primers designed according to (Wach *et al*, 1994; Knop *et al*, 1999) were used. Yeast strains GG628 and GG630 were obtained by transformation of WR1211 and WR1485 with a *RIM15*‐deletion cassette PCR‐amplified from plasmid pFA6ahphMX. PCRs were performed using Q5 polymerase (NEB) according to the manufacturer protocol. All plasmids used in this study are listed in Table EV3. Manipulations of plasmid DNA were carried out using the In‐Fusion® cloning kit (Takara) according to the manufacturer's guidelines. To create plasmids pJF83 (YIPlac211‐9xmyc‐*GIS1*) and pJF97 (YIPlac128‐6xFLAG‐*RPH1*), ORF sequences of *GIS1* and *RPH1* were amplified via PCR from W303‐1A genomic DNA and subcloned into variants of plasmids YIPlac211 and YIPlac128 for n‐terminal tagging. Round‐the‐horn mutagenesis (Moore & Prevelige, 2002) was used to generate 5A mutations of *GIS1* and *RPH1* giving rise to plasmids pJF91 (YIPlac211‐9xmyc‐*GIS1*‐5ALA) and pJF98 (YIPlac128‐6xFLAG‐*RPH1*‐5ALA). Plasmids YIPlac211‐9xmyc‐*GIS1* and YIPlac128‐6xFLAG‐*RPH1* were used as template in PCRs. PCRs were run using Phusion HF DNA polymerase (NEB) according to the manufacturer protocol. Methylated template DNA was digested by DpnI treatment at 37°C for 60 min.

### Growth conditions, SILAC labeling, and harvesting

Yeast cells were grown shaking (200 rpm) at 30°C for at least ten generations until mid‐log phase (OD_600 nm_ ˜1). For MS experiments, SILAC labeling (Ong *et al*, 2002; Gruhler *et al*, 2005) was performed as follows: Yeast cultures were grown in 50 ml synthetic medium (0.17% yeast nitrogen base, 0.5% ammonium sulfate, 2% glucose, amino acids as required) supplemented either with heavy‐ or light‐labeled arginine and lysine (0.05 mg/ml of l‐arginine‐HCl and 0.05 mg/ml of l‐lysine‐2HCl, Euriso‐top), harvested by filtration (Protran 0.45‐μm nitrocellulose membrane, Amersham), and frozen in liquid N_2_. For each experiment, heavy‐ and light‐labeled cultures were grown in parallel and frozen pellets were united after harvesting. Details on the setup of MS experiments are provided in Table EV1. Hyperosmotic stress was induced by addition of 0.5 M NaCl (final concentration) for times indicated. Cdc55 depletion samples were treated with 0.02 mg/ml doxycycline (Sigma‐Aldrich) and 1 mM Auxin (3‐indoleacetic acid, Sigma‐Aldrich) for 30 min prior to hyperosmotic stress application. For mock treatment, H_2_O and ethanol were used, respectively. For M‐Track experiments and gene expression analysis, yeast cultures were grown in 50 ml rich medium (YPD; 1% yeast extract, 2% peptone, 2% glucose), harvested by centrifugation (2 × 1.5 min, 3,000 × *g*), and frozen in liquid N_2_. For growth rate experiments cells were grown in YPD at 30°C overnight. Cultures were adjusted to OD_600 nm_ of 0.1 and grown to OD_600 nm_ 0.8 in fresh YPD at 30°C. 20 µl of the cultures were added to each well of a 96‐well flat‐bottom plate (Corning Inc., Corning, United States) containing 180 µl of fresh YPD media containing different concentrations of NaCl. The final concentration of NaCl ranged from 0 M to 1.25 M as indicated. Plates were incubated at 30°C for 24 h (Cytomat 2, Thermo Fisher, Waltham, United States). Each experiment was run in triplicate on separate microplates. OD_600 nm_ was measured hourly in an automated setup (Synergy H1 reader, Biotek, Winooski, United States; Rack Runner 720, Hamilton Robotics, Martinsried, Germany).

### MS study design

In the study presented here, we compare data from a number of large‐scale MS‐based phospho‐proteomic experiments. In addition, we integrate previously published MS datasets to analyze the behavior of groups of phosphorylation sites in a variety of experimental conditions. The integration of multiple datasets leads to a high number of missing values, which is a well‐known problem of data‐dependent MS studies. To address this issue, we chose the following strategy to analyze the phospho‐proteomic data. We first checked whether changes in protein abundance could potentially confound the quantification of the phosphoproteome. (i) In general, we observed relatively stable levels of protein abundance, suggesting no or only a minor effect on the phosphorylation site ratios (see Fig EV1B). Hence, we did not adjust phosphorylation ratios accordingly because non‐phosphorylated peptide data were not available for all phosphorylation sites. (ii) Instead of filtering for quantification in multiple replicates and statistically testing individual phosphorylation sites, we statistically compared the behavior of defined groups of phosphorylation sites (e.g., stress‐induced phosphorylation sites).

### Protein extraction and enzymatic digestion for MS experiments

Proteins were extracted using TRIzol (Invitrogen) (Reiter *et al*, 2012), or (for setup SR *igo1*Δ*igo2*Δ) using a TCA‐based protocol. TCA extraction was performed as follows: Cells were resuspended in 8 M urea, 50 mM Tris pH 8.0 and disrupted by beat beating using a Fast Prep (three cycles: 45 s, power level 5.5). Insoluble material was removed by centrifugation. Proteins were extracted by addition of ice‐cold TCA (15% final concentration), followed by an incubation for 60 min on ice. Proteins were centrifuged (12,000 × *g*, 5 min, 4°C), washed in 15% TCA and acetone, and shortly dried. Protein pellets were resuspended in 50 mM ammonium bicarbonate (ABC) buffer containing 8 M urea. Protein concentration (2–3 mg/ml) was determined by Bradford protein assay (Bio‐Rad), using bovine serum albumin to create a standard curve. Protein samples were diluted to 50 mM ABC, 6 M urea by using 50 mM ABC. Disulfide bridges were reduced by adding dithiothreitol (DTT), using a DTT to protein ratio of 1:50 (w/w), and samples were incubated for 30 min at 56°C. Cysteines were alkylated by adding iodoacetamide (IAA), using an IAA to protein ratio of 1:10 (w/w), and samples were incubated for 30 min in the dark at room temperature. Remaining IAA was quenched by adding DTT, using a DTT to protein ratio of 1:100 (w/w). Proteins were digested with LysC (Roche) for 2 h at 30°C, using a LysC to protein ratio of 1:100 (w/w). Protein samples were diluted to 50 mM ABC 0.6 M urea by using 50 mM ABC. Proteins were digested with trypsin (Trypsin Gold, Promega) overnight at 37°C, using a trypsin to protein ratio of 1:60. The overnight digest was stopped by adding 100% trifluoroacetic acid (TFA) to a final concentration of 1%. Resulting peptide samples were desalted using Strata‐X reversed‐phase polymeric solid‐phase extraction cartridges Phenomenex, 200 mg) and eluted by addition of 70% acetonitrile (ACN) 0.1% formic acid (FA). An aliquot of ˜1 µg protein extract was taken, diluted with 0.1% TFA to an ACN concentration below 2%, and subjected to MS analysis. Peptide samples were snap‐frozen in liquid nitrogen, lyophilized, and stored at −80°C.

### Phosphopeptide isolation and fractionation

Phosphopeptides were enriched using TiO_2_ (Titansphere bulk media, 5 µm, GL Science). The amount of TiO_2_ resin was adjusted to the peptide concentration (1.25 mg of TiO_2_ / 3.5 mg yeast protein extract). TiO_2_ resin was washed with 50% methanol, H_2_O and equilibrated with TiO_2_ loading buffer (0.8 M phthalic acid, 80% ACN, 0.1% TFA). Dried peptide samples were dissolved in 100 µl TiO_2_ loading buffer and incubated for 1 h with 1 mg TiO_2_ resin per 2.8 mg protein extract. The TiO_2_ resin was transferred to a Mobicol spin column and washed with 2 × 250 µl TiO_2_ loading buffer, 2 × 250 µl 80% ACN 0.1% TFA, 2 × 250 µl 1% ACN 0.1% TFA. Bound phosphopeptides were eluted by addition of 2 × 150 μl 0.3 M ammonium hydroxide and acidified to pH 2.5 by addition of 10% TFA. Phosphopeptide samples were desalted using C18 Sep‐Pak cartridges (Waters), vacuum‐dried, and stored at −80°C. Phosphopeptides were fractionated offline by strong cation exchange chromatography (SCX), using 1 ml Resource S column (GE healthcare) installed in a nano‐HPLC machine (Ultimate 3000, Thermo Fisher Scientific). Briefly, samples were injected using SCX Buffer A (5 mM NaH_2_PO_4_, 30% acetonitrile (ACN), pH 2.7). Peptides bound to the column were separated by a linear gradient of sodium chloride in SCX buffer A. Based upon UV measurements, some fraction containing low amounts of peptide were pooled which resulted in a total of 12 fractions (fractions were collected every minute and then pooled together). Each elution sample was adjusted by TFA to pH 2–3 for subsequent desalting (Rappsilber *et al*, 2007) and mass spectrometry measurement.

### Poly‐histidine, biotinylation signal tandem affinity (HB) purifications

For HB pull‐downs, one liter of yeast cell culture was harvested by filtration, deep‐frozen, and ground using a SPEX Freezer Mill 6870 (SPEX SamplePrep) applying standard settings (Reiter *et al*, 2012). Lysed cells were resolved in buffer 1 (6 M guanidine HCl, 50 mM Tris pH 8.0, 5 mM NaF, 1 mM PMSF, 2 mM sodium orthovanadate 0.1% Tween, protease inhibitor cocktail (Roche), pH 8), cleared by centrifugation (13,500 × *g*, 15 min, 4 °C), incubated with Ni^2+^−Sepharose beads (GE Healthcare) for 4 h at room temperature, and washed with urea buffer (8 M urea, 50 mM sodium phosphate buffer pH 8.0 (and pH 6.3), 300 mM NaCl, 0.01% Tween 20). Proteins were eluted in urea buffer pH 4.3 containing 10 mM EDTA, incubated with streptavidin–agarose beads (Thermo Fisher Scientific), washed with urea wash buffer containing 1% SDS and without SDS. For on bead digests, samples were re‐buffered to 50 mM ammonium bicarbonate (ABC), reduced using DTT, treated with 20 mM iodoacetamide (IAA) to alkylate cysteine residues, incubated with 300 ng trypsin (Trypsin Gold, Mass Spectrometry Grade, Promega) at 37 °C overnight, quenched with trifluoroacetic acid (0.5% final concentration), and desalted using C18 StageTips (Rappsilber *et al*, 2007).

### Mass spectrometry measurements

LC‐MS/MS analysis was performed on an UltiMate 3000 Dual LC nano‐HPLC System (Dionex, Thermo Fisher Scientific), containing both a trapping column for peptide concentration (PepMap C18, 5 × 0.3 mm, 5 µm particle size) and an analytical column (PepMap C18, 500 × 0.075 µm, 2 µm particle size, Thermo Fisher Scientific), coupled to a Linear Trap Quadrupole Orbitrap Velos (with CID, collision‐induced dissociation mode; or ETD, electron‐transfer dissociation) mass spectrometer (Thermo Fisher), a Q Exactive HF Orbitrap (with HCD, higher‐energy collisional dissociation mode) mass spectrometer (Thermo Fisher), or a Orbitrap Lumos Tribrid (with HCD) mass spectrometer (Thermo Fisher) via a Proxeon nanospray flex ion source (Thermo Fisher). For peptide separation on the HPLC, the concentration of organic solvent (acetonitrile) was increased from 2.5 to 40% in 0.1% formic acid at a flow rate of 275 nl/min, using different gradient times. For acquisition of MS2 spectra, the instruments were operated in a data‐dependent mode with dynamic exclusion enabled. A detailed description of the acquisition settings for individual raw files is listed in Dataset EV3.

HB purifications were analyzed on a Q Exactive HF Orbitrap (Thermo Fisher Scientific) mass spectrometer applying the following settings: Peptides were separated applying an increasing organic solvent (acetonitrile) gradient from 2.5 to 40% in 0.1% formic acid over 120 min at a flow rate of 275 nl/min. MS1 resolution was set to 120 k, AGC 3E6. MS2 resolution was set to 30 k, AGC 1E5, 250 ms max. IIT. The mass spectrometer was configured to pick the eight most intense precursor ions for data‐dependent MS2 scans, applying HCD for fragmentation with a normalized collision energy (NCE) of 27. Dynamic exclusion time was set to 30 s.

### Mass spectrometry raw data files used in this study

MS raw files and MS data analysis results from this study have been deposited to the ProteomeXchange Consortium (Vizcaíno *et al*, 2016) via the PRIDE partner repository with the dataset identifier PXD019646. Previously published MS raw files from Romanov *et al* (2017) that were reanalyzed in this study are available in the PRIDE repository with the dataset identifiers: PXD004294 to PXD004300 (Data ref: Romanov *et al*, 2017).

### Mass spectrometry data analysis with MaxQuant

Raw MS data were analyzed using MaxQuant (Cox & Mann, 2008) software version 1.5.2.8 (global proteome experiments) or with version 1.6.0.16 (Gis1 and Rph1 HB purification experiments), using default parameters with the following modifications. MS2 spectra were searched against a protein database from the SGD (Saccharomyces Genome Database, www.yeastgenome.org, version 3^rd^ February 2011) containing 6,717 entries, concatenated with a database of common laboratory contaminants (provided with MaxQuant). Hence, the option to include contaminants was deactivated. Enzyme specificity was set to “Trypsin/P” (allowing cleavage after proline), the minimal peptide length was set to 6 and the maximum number of missed cleavages was set to 2. The option “I = L” was activated to treat the amino acids leucine and isoleucine as indistinguishable. The minimum peptide length was set to 6. Carbamidomethylation of cysteine was defined as fixed modification. “Acetyl (Protein N‐term)”, “Deamidation (NQ)”, “Oxidation (M)” and “Phospho (STY)” were set as variable modifications. A maximum of 6 variable modifications per peptide was allowed. For MS measurements of samples prior to phosphopeptide enrichment “Phospho (STY)” was not used as a variable modification. For SILAC quantification, “multiplicity” was set to 2, “Arg6” and “Lys6” were specified as heavy labels, “Requantify” and “Match between runs” were enabled.

### Calculation of phosphorylation site SILAC ratios

For calculation and normalization of phosphorylation site, SILAC ratios in‐house Python scripts were used (see Data and Code Availability section). All data were extracted from MaxQuant evidence tables. SILAC ratios (heavy to light) were extracted from the column “Ratio H/L”, log2‐transformed and, if necessary, inverted (see Table EV1). SILAC ratios were corrected for differences in the amount of heavy‐labeled and light‐labeled cells. In addition, proline containing peptides were corrected for signal loss caused by the conversion of heavy‐labeled arginine to heavy‐labeled proline (Ong *et al*, 2003). Normalization factors were calculated independently for each replicate and experiment. For calculation of normalization factors, only unphosphorylated peptides were considered. First, the average log2 ratio of peptides, not containing proline, was calculated and subtracted from the log2 ratios of individual phosphorylated and unphosphorylated peptides. Second, a proline‐conversion factor was calculated as the average log2 ratio of unphosphorylated peptides containing exactly one proline and the log2 ratio (divided by two) of peptides containing two prolines. For each phosphorylated and unphosphorylated peptide, the proline‐conversion factor was multiplied by the number of prolines present in the peptide sequence and subtracted from the log2 ratio. To correct for differences in protein abundances of Gis1 and Rph1 in the HB purification experiments, all peptides of the respective protein were normalized by subtracting the median log2 ratio of the unphosphorylated peptides. An isoform phosphorylation site probability was calculated by multiplying the highest individual phosphorylation site probabilities. Peptides with an isoform probability below 70% were discarded. To facilitate interpretation of protein phosphorylation sites, phosphopeptides were grouped into “phosphorylation sites” containing the same set of phosphorylated protein residues, regardless of potential missed cleavages or additional modifications such as oxidation. The SILAC log2 ratio of individual “phosphorylation sites” was calculated independently for each replicate and experiment as the average log2 ratio of all corresponding evidence table entries. The replicate ratios were then averaged for the final ratio of the “phosphorylation site”.

For analysis of the HB purification experiments, peptides were sorted into “phosphoislands” (Langella *et al*, 2017; Repetto *et al*, 2018) that contain adjacent phosphorylation sites covered by overlapping peptides (Dataset EV2). Phosphoislands were generated by grouping phosphopeptides that overlap by at least one of the phosphorylated amino acids. The amino acid sequence and number of phosphosites may vary between the overlapping peptides. For each phosphoisland, one or multiple phosphorylation sites and one unphosphorylated counter group (UCG) were defined, which contains all unphosphorylated peptides that overlap with at least one of the phosphoisland’s phosphorylation sites. The SILAC log2 ratios of individual UCGs were calculated as described for phosphorylation sites.

### Integration of published mass spectrometry datasets without raw data re‐analysis

For integration of the dataset characterizing the effect of Cdc28 inhibition on the phosphorylome, we used in‐house Python scripts (see Data and Code Availability section). SILAC ratios were extracted from Kanshin *et al* (2017) “Supplemental Table S1”, using the column “*t*‐test Difference_CDK”, and integrated into Dataset EV1 as “Setup Cdc28 inhibition”.

### Selection of sites for mutational analysis

Phosphorylation sites for mutational analysis were selected according to whether they become increasingly induced by Cdc55 depletion in a time‐dependent manner and are also found to be induced in setup SR. Only two phosphorylation sites on each protein fulfilled these strict criteria, Ser^425^ and Ser^690^ of Gis1 and Ser^412^ and Ser^430^ of Rph1. Given that both proteins are paralogues, regulated phosphorylation sites that are conserved between these proteins could also be potentially interesting (Strumillo *et al*, 2019), i.e., Ser^425^ of Gis1 being homologous to Ser^430^ of Rph1. Ser^405^ of Gis1 (homologue to Ser^412^ of Rph1) and Ser^584^ of Rph1 (homologue to Gis1 Ser^690^) were therefore also included in the mutational analysis. The MS data suggest Cdc55 dependency for both of these phosphorylation sites. Moreover, Ser^405^ of Gis1 and Ser^412^ of Rph1 lie in a conserved cluster of three juxtaposed phospho‐accepting residues with occasional ambiguous phosphorylation site allocation in some peptide hits. An additional serine‐to‐alanine mutation was therefore introduced in this cluster, namely at Ser^403^ of Gis1 and at Ser^410^ of Rph1. A second conserved cluster affected by both stress and Cdc55 depletion was observed, however, with decreasing phosphorylation levels (at Ser^696^ of Gis1 and Ser^590^ of Rph1) in response to the experimental conditions.

### Fluorescence‐activated cell sorting (FACS) analysis of DNA content

For FACS analysis, 1 ml of early log‐phase cells was harvested by centrifugation and fixed with 1 ml 70% ethanol for 3 h at room temperature. Fixed cells were pelleted by centrifugation (1 min, 12,000 × *g*), resuspended in 0.5 ml RNase buffer (50 mM Tris/HCL (pH 7.5), 200 µg/ml RNase A) and incubated overnight at 30°C. Cells were pelleted by centrifugation (1 min, 12,000 × *g*), resuspended in 0.5 ml FACS buffer (50 mM Tris/HCL (pH 7.5), 55 µg/ml propidium iodide) and incubated for 1 h at room temperature. After sonication with 5 short pulses (20% of max output), 20 µl of the cell suspension was diluted in 1 ml 50 mM Tris/HCL (pH 7.5). For each sample, 10,000 cells were analyzed using a FACSCalibur cell analyzer (BD Biosciences). Data were analyzed using FlowCytometryTools (version 0.4.6), a python package for visualization and analysis of high‐throughput flow cytometry data (Data ref: Yurtsev & Friedman, 2015). We specifically looked at the FL3‐H channel values and normalized the data to the respective 2C peak. For visualization purposes, a Gaussian density curve was laid over the histogram values.

### Protein–protein proximity assay (M‐Track)

For M‐track assays (Zuzuarregui *et al*, 2012; Brezovich *et al*, 2015), protein extraction was carried out in RLB+ buffer (1× PBS, 10% glycerol, 0.5% Tween 20, 1 mM NaF, 20 mM ß‐glycerophosphate, 1 mM PMSF, 1 mM Na‐vanadate, protease inhibitor cocktail (Roche)) at 4°C. Cell extracts were prepared by glass bead lysis using a Fast Prep 24 instrument (MP Biomedicals) with the following settings: 3× 30 s, power level 5.5. Prey proteins were enriched by immunoprecipitation using Dynabeads Pan Mouse IgG (Thermo Fisher Scientific). After washing with RLB+ and RLB buffer (1× PBS, 10% glycerol, 0.5% Tween 20, 1 mM NaF, 20 mM ß‐glycerophosphate, 1 mM PMSF, 1 mM Na‐vanadate), prey proteins were eluted by boiling for 1 min in 2× urea sample buffer (8 M urea, 80 mM Tris/HCl pH 6.8, 2 mM EDTA pH 8, 2% β‐mercaptoethanol, 4% SDS, 4% glycerol). Eluates were analyzed by Western blotting. Histone H3 lysine 9 trimethylation (me3K9H3) of protA‐H3‐HA tags was visualized using an antibody recognizing me3K9H3 (1:2,000 dilution in 1% yeast extract (YE) in PBS‐T, Novus #NBP1–30141—note: we observed strong differences in the quality and specificity of the antibody between different batches; different batches were used in series 1 and series 2). Membranes were incubated with primary antibody for 1 h at 4 °C, followed by 1 h incubation at 4 °C with HRP‐conjugated goat anti‐mouse (1:5,000 dilution in 1% YE in PBS‐T, Bio‐Rad #170–6516) secondary antibody. No washing steps were performed between primary and secondary antibody incubation. Loading was controlled using an antibody recognizing HA (1:5,000 in PBS‐T, 12CA5). PicoECL (Thermo Scientific) was used for enhanced chemiluminescent (ECL) detection.

Peak areas of me3K9H3 and HA signals were determined by densitometric analysis of scanned Western blot films using ImageJ and log2‐transformed. Each Western blot experiment contained a four‐point dilution series of a control sample. A second‐degree (series 1) or first‐degree (series 2) polynomial curve was fitted independently to the me3K9H3 and HA signals of to the dilution series, which was used to correct me3K9H3 and HA signals for unequal loading amounts between samples and to normalize between different Western blot experiments. Proximity signals were calculated as the difference of the normalized log2‐transformed me3K9H3 and HA signal intensity and rescaled by subtracting the mean proximity signal of the negative control (Cdc55‐protA‐H3‐HA, no HKMT). A one‐tailed Welch’s *t*‐test was used to identify the statistically significant candidates. For each candidate, the proximity signal of all replicates was compared against all proximity signals of the negative control (Cdc55‐protA‐H3‐HA, no HKMT). P‐values were corrected for multiple testing by using the Benjamini–Hochberg procedure to generate q‐values.

M‐track protein–protein proximity analysis of yeast strains GG628 (*rim15*Δ, *GIS1*‐HKMT, *CDC55*‐protA‐H3HA) and GG630 (*rim15*Δ, *RPH1*‐HKMT, *CDC55*‐protA‐H3HA, Fig 5B) was carried out using MES lysis buffer (50 mM MES/NaOH pH 6.5, 150 mM NaOH, 1% Triton, 1 mM EDTA, complete EDTA free (Roche)). Cell extracts were prepared by glass bead lysis using a Fast Prep 24 instrument (MP Biomedicals) with the following settings: 1 × 45 s, power level 6.5. Immunoprecipitation of prey proteins was achieved using anti‐HA‐magnetic beads (Pierce). Washing steps were carried out using a MES‐wash buffer (50 mM MES/NaOH pH 6.5, 150 mM NaOH, 1 mM EDTA). Prey proteins were eluted by heat incubation in a 2× urea sample buffer. Each sample was mixed with 4 µl of a HeLa lysate (protein concentration 1 mg/ml in urea sample buffer) before loading in order to improve Western blot transfer efficiency rates.

### Phospho‐shift assay

Cell lysis was carried out in MES lysis buffer (50 mM MES/NaOH pH 6.5, 150 mM NaOH, 1% Triton, 1 mM EDTA, Complete EDTA free (Roche)) by beating using a Fast Prep 24 instrument (MP Biomedicals) with the following settings: 3 × 45 s, power level 6.5. Cleared protein extracts were resolved in SDS–PAGE loading buffer (62.5 mM Tris–HCl (pH 6.8), 8 M urea, 2% (w/v) SDS, 0.05% (w/v) bromophenol blue, 10% (v/v) glycerol, 5% (v/v) β‐mercaptoethanol) and incubated at 95°C for 1 min. For Mn^2+^‐Phos‐tag SDS–PAGE, 25 µM of Phos‐tag‐AAL (Wako) and 50 µM of MnCl_2_ were added to the separating gel before polymerization. The pH of the separation gel was adjusted to 8 for Mn^2+^‐Phos‐tag SDS–PAGE and 8.8 for standard SDS–PAGE. Gelshifts were visualized by Western blot using an antibody recognizing HA (12CA5). Hyperosmotic stress was controlled by monitoring phosphorylation of MAPK Hog1 using an antibody against phospho‐p38 (Cell Signaling, Phospho‐p38 MAPK (Thr180/Tyr182) Antibody #9211, 1:5,000), protein levels of Cdc55 by using an antibody against Cdc55 (Max Perutz Labs Vienna, Monoclonal Antibody Facility, 1:200 (Zuzuarregui *et al*, 2012)). Loading was controlled using an antibody recognizing Cdc28 (HPA030762, Sigma‐Aldrich, 1:10,000).

### Gene expression analysis

Total RNA isolation was adapted from Cross and Tinkelenberg (Cross & Tinkelenberg, 1991). 20 ml of early log‐phase cells was harvested by centrifugation (2 min, 4 × *g*, 4°C) and resuspended in 200 µl TE buffer. Cell breakage was performed using approximately 200 µl glass beads and 200 µl phenol–chloroform–isopropanol (25:24:1 in 10 mM Tris/HCl, pH 8.0) in a Fast Prep 24 instrument (MP Biomedicals) for 2 × 10 s at power level 6. After centrifugation (15 min, 20,000 × *g*, 4°C), the upper aqueous phase was washed twice (mixed and centrifuged 10 min, 20,000 × *g*, 4°C) with an equal volume of chloroform–isopropanol (24:1). 100 µl of the aqueous phase was transferred to a new tube; precipitation was performed in 0.04 M sodium acetate, 64% ethanol. Samples were incubated for 30 min or overnight at −20°C. RNA was pelleted (10 min, 20,000 × *g*, 4°C), supernatant was removed completely, and the RNA pellet was resuspended in 50 µl ddH_2_O to yield an RNA concentration of 4–8 µg/µl. RNA samples were treated with RNase‐free DNase (Thermo Fisher, 1 U/µg RNA) at 37°C for 30 min, the reaction was stopped by adding 1 µl of 50 mM EDTA at 65°C for 10 min, and cDNA was synthesized using GoScript Reverse Transcriptase (Promega) with oligo(dT) primers (Microsynth). RT‐qPCR analyses were performed on cDNA templates using RT‐qPCR master mix containing SYBR Green I nucleic acid gel stain (Sigma) for the time‐course experiments and LUNA master mix (NEB) for the 30 min time point experiments on 96‐well plates in a Mastercycler Realplex 2 (Eppendorf). The following gene‐specific primers were used. *CTT1* Fw: AAT CAG TTT CAG GAC ACT ACC, *CTT1* Rv: GAA TGA CCA GAG TAC GCG TTC, *PGM2* Fw: CTC TGG TTT GCG TAA GAA GAC, *PGM2* Rv: TTG TAG TAA CGC CCA TCA CC, *IPP1* Fw: GAT GGT AAG CCA GTT TCT GC, *IPP1* Rv: GGT TCA AAG TTT CTT CCT TGG. Specificity of RT‐qPCRs was verified by agarose gel electrophoresis and melting curve analysis. A single band of the expected size and a single peak, respectively, were required. Quality of mRNA was analyzed on Agilent 2100 Bioanalyzer Instrument (Assay Class: Eukaryote Total RNA Nano, version 2.6) and confirmed to constitute RIN values of > 7 for all 30 min time point samples (Appendix Fig S3A and B). Additionally, amplification efficiencies for each target gene (*CTT1*, *PGM2*) and the reference gene (*IPP1*) in LUNA master mix (NEB) were determined by standard curves obtained from serial dilution reactions and reached > 90%. Gene expression data were collected from at least 3 biological replicates and technical duplicates (time‐course data) or triplicates (30 min time point data) per sample. LG *Cq* values (averaged over technical replicates) of target genes were corrected for efficiency of the respective assay, and gene expression levels were determined by relative quantification based on the *Cq* values of the reference gene in the same sample (Δ*Cq* method). All datasets (one set = all samples per biological replicate) were evaluated individually for technical integrity by checking for extreme outliers, failed individual reactions, and missing data, and were considered valid if more than 75% of the reaction plate were uncompromised. Each valid biological replicate was then normalized over the sum of all values that were uncompromised across the entire experiment and integrated to compare expression levels between samples.

### Determination of growth rates

Growth curves were fitted using the GROFIT package in R (Kahm *et al*, 2010). GROFIT was used to calculate the growth rate µ [h^−1^] from each growth curve. Growth rates were then given as mean ± standard deviation of triplicates. Underlying data are shown in Dataset EV4.

### Serial dilution droplet test

W303‐1A wild‐type and deletion mutant strains were grown to mid‐log phase. OD_600 nm_ values of cultures were equalized to 0.1, and serial dilution steps of 1:7 dilutions were prepared. Droplets of 2.5 μl were transferred onto a hyperosmotic stress (YPD +0.8 M NaCl) and onto a YPD plate with no additives as a control. Plates were incubated at 30°C, and growth was monitored for 4 days.

### GO‐term analysis

GO enrichment analysis was performed with DAVID (version 6.8) (https://david.ncifcrf.gov/) (Huang *et al*, 2009), based on GO terms “Biological Process” with GO hierarchy levels ranging from level 3–6. Only gene ontology terms with corrected *P* < 0.05 are considered.

### MotifX and threonine enrichment

We used the MotifX algorithm implemented in R (Schwartz & Gygi, 2005; Chou & Schwartz, 2011; Wagih *et al*, 2016) to identify overrepresented linear motifs within specific sets of phosphorylation sites. A sequence window of 13 residues (± 6 residues around the phosphorylated amino acid) was selected to extract phosphorylation motifs. Serine and threonine containing phosphorylation sites were analyzed independently of each other. In case of multiple phosphorylated sites per peptide, each phosphorylated residue was considered individually. The following settings were used to run the R script: minimal sequences 20, *P*‐value cutoff 0.01. For the threonine enrichment analysis, the MotifX input sequences were used and Fisher’s exact test was applied to assess statistical significance.

## Author contributions

DMH and WR conceptualized the study. DMH, CS, EO, GA, and WR designed experiments. DMH, GG, NR, JF, JV, RB, CS, and WR performed experiments. DMH, NR, MJ, CS, and MH analyzed the data. DMH, NR, and WR wrote the paper. All authors edited the text. All authors read and approved the final manuscript.

## Conflict of interest

The authors declare that they have no conflict of interest.

## Supporting information



Appendix

Expanded View Figures PDF

Table EV1

Table EV2

Table EV3

Dataset EV1

Dataset EV2

Dataset EV3

Dataset EV4

Source Data for Appendix

## Data Availability

The datasets produced in this study and the scripts used for data analysis are available in the following databases:
Phospho‐proteomic MS data: PRIDE PXD019646 (http://www.ebi.ac.uk/pride/archive/projects/PXD019646). Python scripts used for data analysis: GitHub (https://github.com/hollenstein/sourcecode_cdc55).

## Footnotes

^‡^
Correction added on 4 November 2021, after first online publication: A typo in *cdc55∆* has been corrected.
