# Supplementary material for: A phosphatase‐centric mechanism drives stress signaling response
Source: EMBO Rep. 2021 Sep 24;22(11):e52476. doi: 10.15252/embr.202152476 (PMC8567219; doi:10.15252/embr.202152476)
Supplement: Supplementary file 1 — Appendix [file EMBR-22-e52476-s003.pdf]

APPENDIX

A phosphatase-centric mechanism drives stress signaling response

Hollenstein D., Gérecová G., Romanov N., Ferrari J., Veis J., Janschitz M., Beyer R., Schüller C., Ogris E., Hartl M., Ammerer G. and Reiter W.

Appendix Figure S1 .... page 2

Appendix Figure S2 .... page 3

Appendix Figure S3 .... page 4

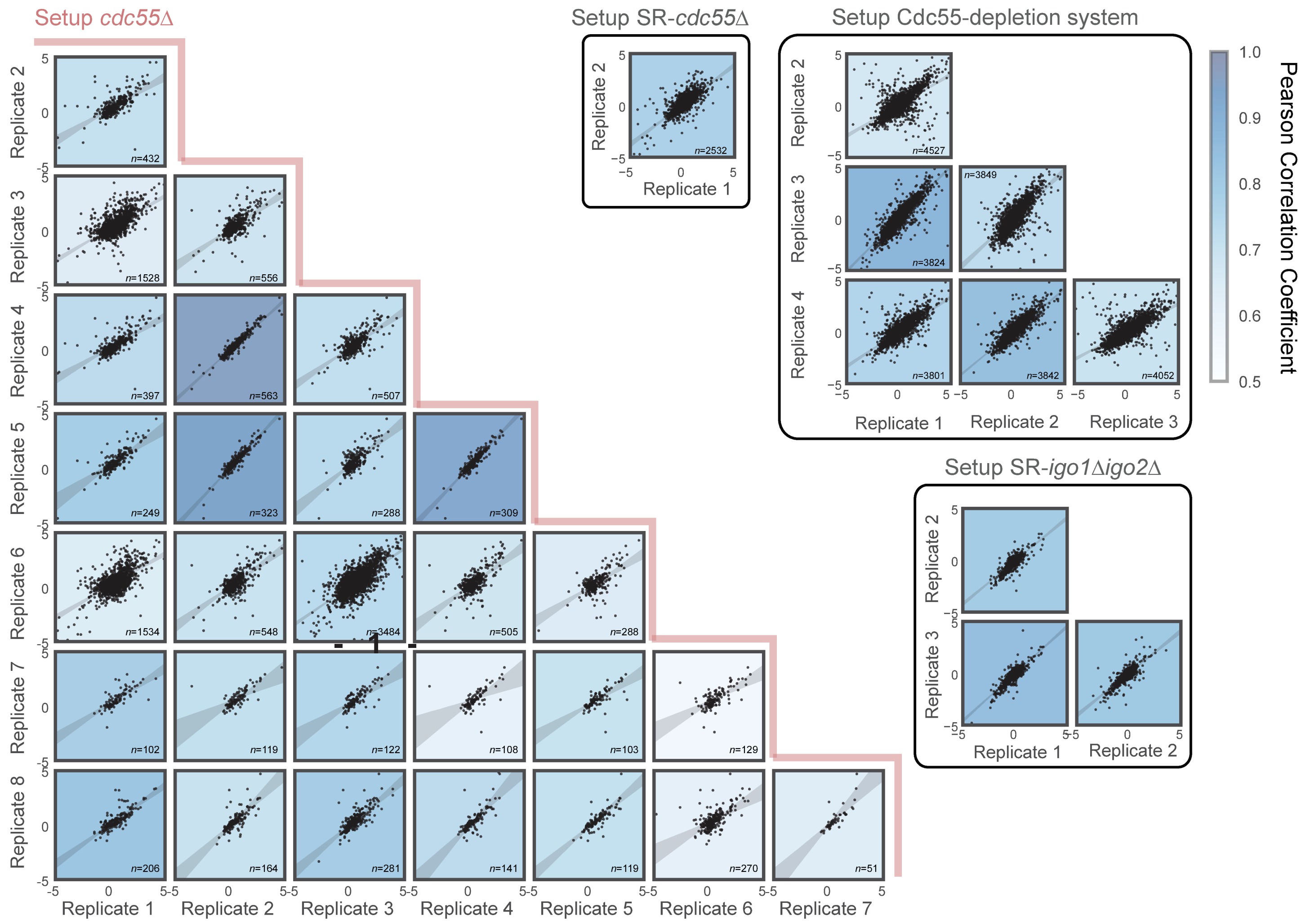

Appendix Figure S1 (related to Figure 1). SILAC ratios are reproducible across biological replicates (illustrated for setup *cdc55*Δ, and other indicated MS setups). For each scatter plot the background color reflects the underlying Pearson correlation coefficient. The number of data points is shown in each scatter (n), and the shaded grey bands indicate the regression estimate at the confidence interval of 0.9.

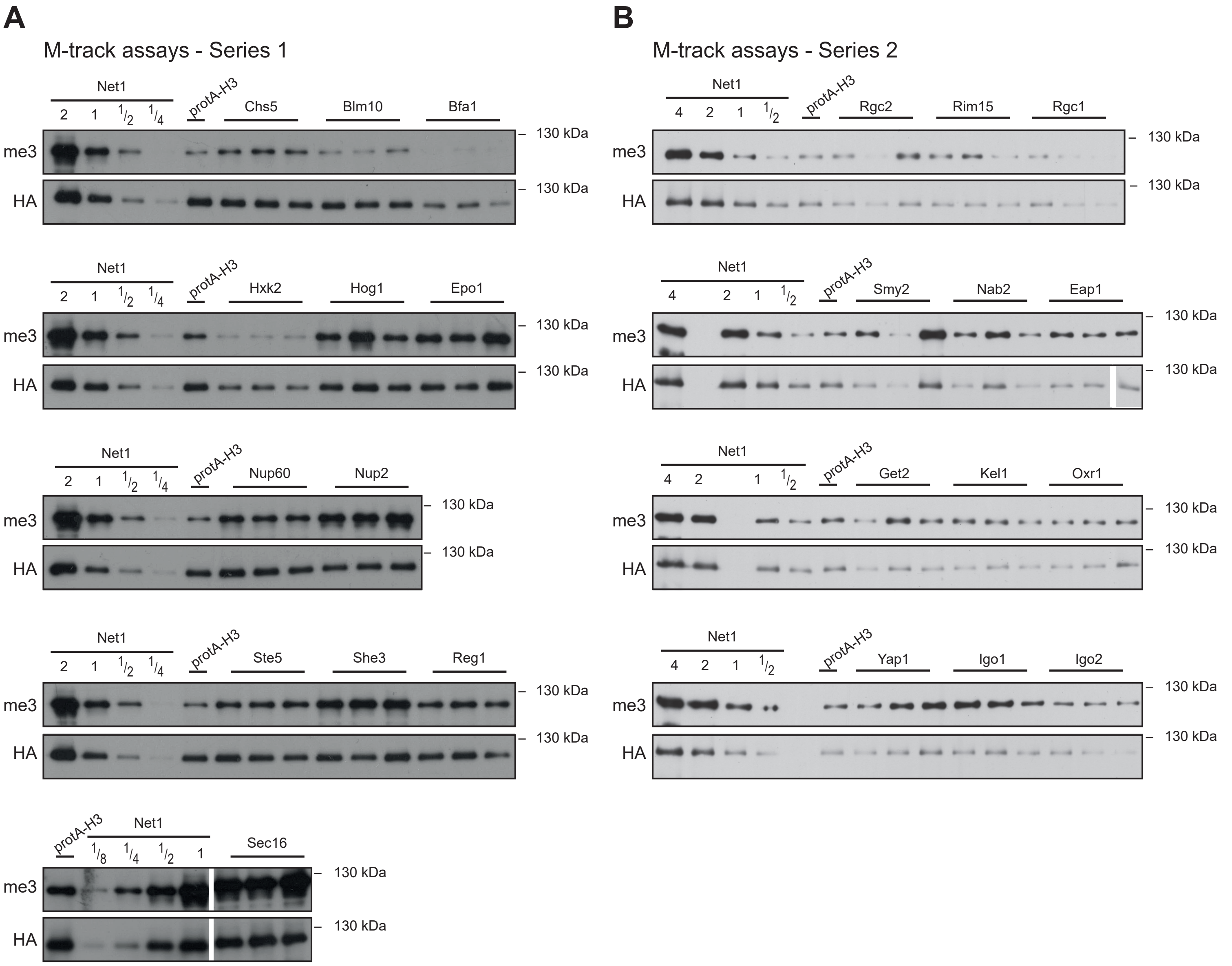

Appendix Figure S2 (related to Figure 3D). Scanned western blot films showing M-track protein-protein proximity results of Cdc55 and the individual candidates (in 3 replicates, respectively). (A) Experimental series 1. me3: antibody recognizing me3K9H3; HA: 12CA5 antibody. (B) Same as (A) except that results obtained from experimental series 2 are shown. Note: Samples in (A) and (B) that were run in a different order (on the same membrane) than presented in the figure are separated with white demarcating lines. For inspection of the original western blot data please refer to the source data figure.

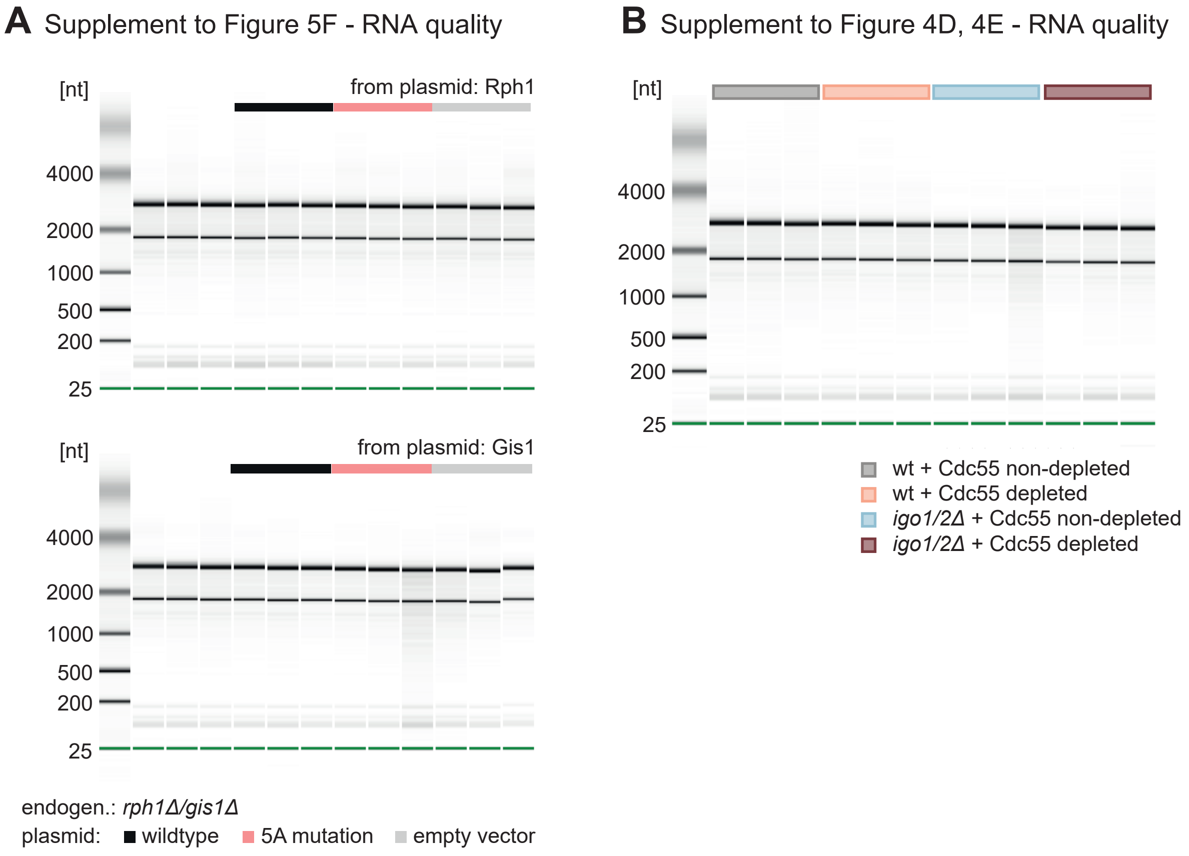

Appendix Figure S3 (related to Materials and Methods, Figure 4E and 4D). (A) Agilent 2100 Bioanalyzer chip data of one replicate within the 30'minute time point analysis shown in Fig 5F, RNA quality was high (RIN > 7) across all technical and biological replicates. Unmarked samples are not part of this manuscript. (B) Agilent 2100 Bioanalyzer chip data of one replicate within the 30'minute time point analysis shown in Figure 4D and 4E (iAID-Cdc55 depletion), RNA quality was high (RIN > 9.0) across all technical and biological replicates.
