# Supplementary material for: A phosphatase‐centric mechanism drives stress signaling response
Source: EMBO Rep. 2021 Sep 24;22(11):e52476. doi: 10.15252/embr.202152476 (PMC8567219; doi:10.15252/embr.202152476)
Supplement: Supplementary file 2 — Expanded View Figures PDF [file EMBR-22-e52476-s006.pdf]

Expanded View Figures

Figure EV1. Experimental workflow and quality control (related to Fig 1).

A Schematic illustration of the applied experimental workflow. Yeast cells were isotopically labeled using SILAC. Proteins were extracted using the TRIzol (Invitrogen) reagent and digested with trypsin. Phosphopeptides were enriched using TiO_2 beads and fractionated offline by strong cation exchange chromatography (SCX). Peptide samples were analyzed by LC-MS/MS, using an ESI-LTQ-Orbitrap Velos or ESI-Q Exactive HF system (Thermo Scientific Fisher). The lower panel of the scheme illustrates the MS data analysis workflow, which was done with MaxQuant. After normalization of SILAC ratios, MS datasets were integrated and compared. SILAC ratios of phosphorylation sites were log2-transformed with positive values indicating increased phosphorylation in the knockout and *vice versa*.

B Protein abundance is not affected by deletion of *CDC55*, with 95% of all SILAC ratios clustering between −1 and 1 (log2). Histograms and boxplots illustrate SILAC ratios of unphosphorylated (gray) and phosphorylated peptides (red) from setup *cdc55*Δ. *P*-values were calculated using a *t*-test.

C Bar plot displaying percentage of SILAC ratios stratified according to their value (ratio ≥ 2, ratio ≤ 0.5, and static) in setup *cdc55*Δ and - *rts1*Δ.

D Scatter plot comparing SILAC ratios of stress-induced phosphorylation sites (≥ 2-fold change in *SR*) between setups - *cdc55*Δ (*y*-axis) and - *rts1*Δ (*x*-axis). Commonly regulated phosphorylation sites are illustrated in colors and annotated.

E Wild-type and *cdc55*Δ cells or Cdc55-depleted and non-depleted cells were exposed for 0, 5, and 10 min to hyperosmotic stress (0.5 M NaCl). Cell cycle distribution was monitored by fluorescence-activated cell sorting (FACS) analysis of DNA content. Representative FACS profiles are shown (upper). Western blot analysis was used to validate Cdc55 depletion (lower).

F Table presenting log2 SILAC ratios from setup *cdc55*Δ or Cdc55 depletion across sites of cell cycle-related markers defined by Ref. (Kelliher *et al*, 2018).

© 2021 The Authors

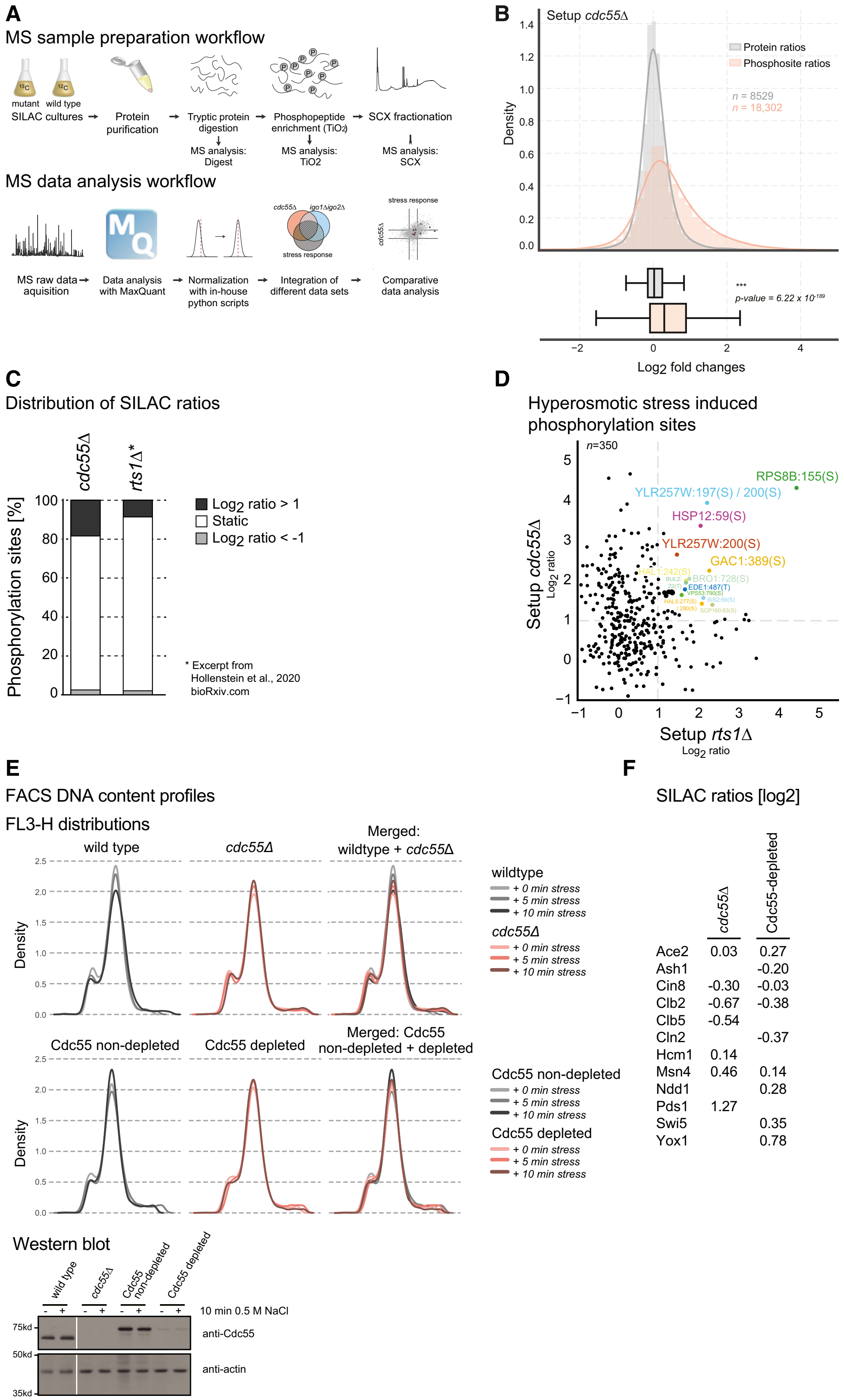

Figure EV1.

 © 2021 The Authors

Figure EV2. The Greatwall Kinase-Endosulfin-PP2A module plays a role in the hyperosmotic stress response (related to Fig 2).

A Phosphorylation site maps of Rim15, Igo1, Igo2, and Cdc55. Phosphorylation sites covered in our analysis are indicated in black (static in setup SR) or red (affected by osmostress). Phosphorylation sites listed in either PhosphoGRID (Sadowski *et al*, 2013) or Uniprot (UniProt Consortium, 2019) databases are indicated in gray. Green: target sites of indicated kinase or phosphatase activity (not covered in our analysis). Pkc1 sites were taken from (Thai *et al*, 2017).

B Table presenting log2 SILAC ratios across sites of Rim15, Igo1 and Igo2, and Cdc55 that were affected by hyperosmotic stress treatment (abs(log2 fold change) ≥ 1).

C, D Excerpt of published MS datasets showing SILAC log2 ratios of Igo1 and Igo2 key phosphorylation sites after hyperosmotic stress exposure: (C) within the first 60 s after treatment with 0.4 M NaCl (Kanshin *et al*, 2015); (D) at 0, 5, 15, and 30 min after treatment with 0.5 M NaCl (Janschitz *et al*, 2019).

E Serial dilution spot assay monitoring growth of wild-type, *cdc55*Δ, *igo1*Δ*igo2*Δ, and *igo1*Δ*igo2*Δ*cdc55*Δ cells under non-stress conditions (upper panel) and in response to high osmolarity conditions (lower panel).

F Histograms displaying the SILAC ratios (log2) of stress- and *cdc55Δ*-induced phosphorylation sites in two separate experiments (orange and blue). The gray histograms only include phosphorylation sites that are neither affected by stress nor the absence of *CDC55*. Left histogram shows the distribution of fold-changes after deletion of *CDC55* and upon exposure to hyperosmotic stress (setup SR *cdc55*Δ). Right: Distribution of fold-changes when *IGO1* and *IGO2* are deleted and cells are exposed to hyperosmotic stress (setup SR *igo1*Δ*igo2*Δ). The same set of sites was compared between the two experiments. *P*-values were calculated using the Wilcoxon test. The indicated distributions are significantly different.

G Scatter plot displaying log2 SILAC ratios of phosphorylation sites in the experiment setup SR on the *x*-axis and setup SR *igo1*Δ*igo2*Δ on the *y*-axis. Phosphorylation sites that do not exhibit Hog1 dependence are shown in gray, whereas Hog1-dependent phosphorylation sites are highlighted in black.

© 2021 The Authors

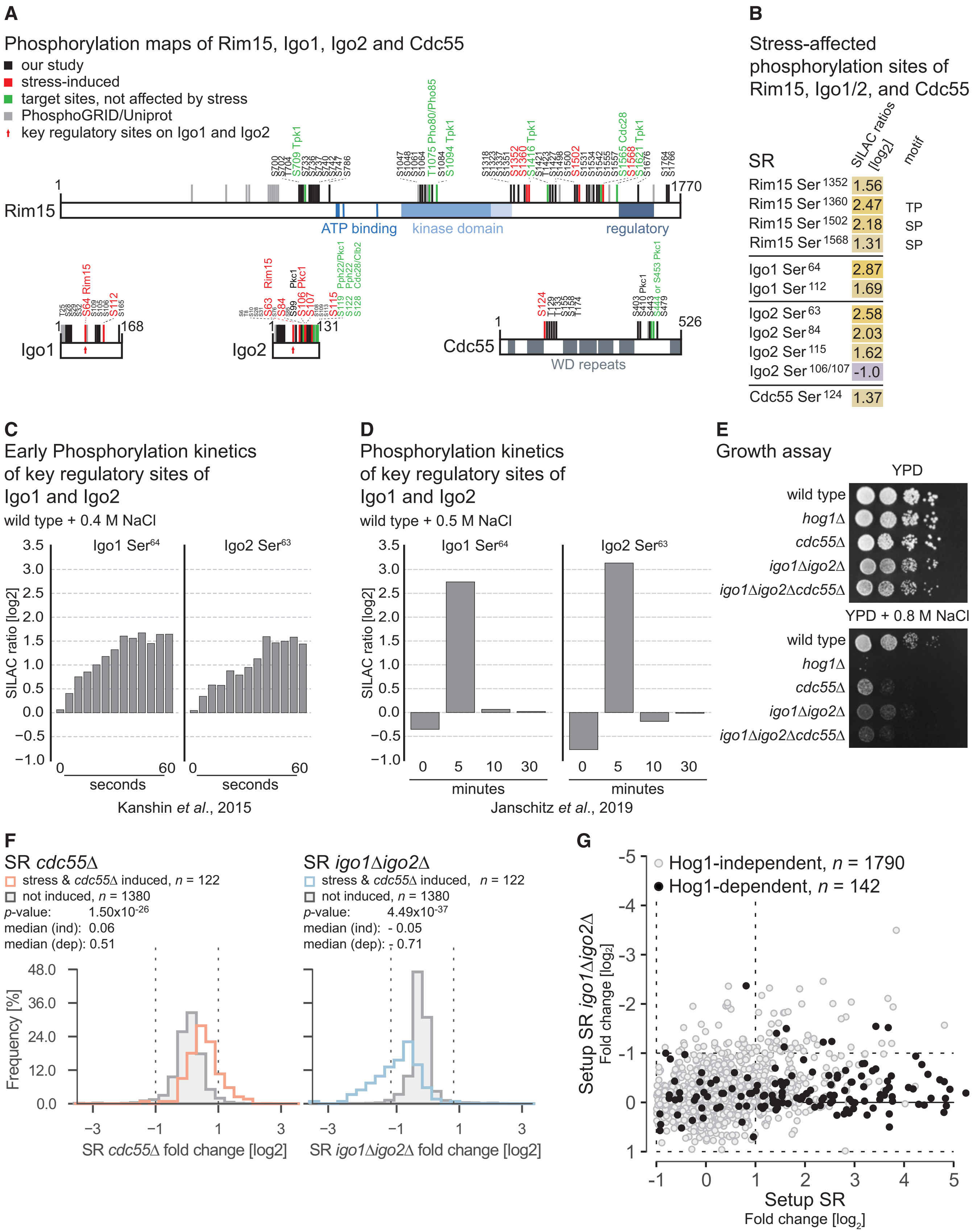

Figure EV2.

 © 2021 The Authors

Figure EV3. Stress- and Cdc55-affected S/T-P motifs are not targeted by Cdc28 or Hog1 (related to Fig 3).

A Histogram displaying SILAC ratios (log2) from the Cdc28 inhibition dataset (Kanshin *et al*, 2017). The gray histogram includes only stress-induced phosphorylation sites, and the orange histogram includes phosphorylation sites that are induced in setup *cdc55Δ*.

B Scatter plots comparing the Cdc28 inhibition dataset (Kanshin *et al*, 2017) with two other experiments. Left scatter plot displays log2 SILAC ratios of phosphorylation sites in the setup *cdc55Δ* on the x-axis and the Cdc28 inhibition dataset on the y-axis. Right scatter plot displays log2 SILAC ratios of phosphorylation sites in the setup SR *igo1*Δ*igo2*Δ on the x-axis and the Cdc28 inhibition dataset on the y-axis. Phosphorylation sites that do not exhibit stress dependence are shown in gray, whereas stress-induced phosphorylation sites are highlighted in black.

C Scatter plots comparing the effect of Hog1 inhibition on the stress phosphorylome (setup SR Hog1 inhibition) with two other experiments. Left scatter plot displays log2 SILAC ratios of phosphorylation sites in the setup *cdc55Δ* on the x-axis and the setup SR Hog1 inhibition on the y-axis. Right scatter plot displays log2 SILAC ratios of phosphorylation sites in the setup SR *igo1*Δ*igo2*Δ on the x-axis and the setup SR Hog1 inhibition on the y-axis. Phosphorylation sites that do not exhibit stress dependence are shown in gray, whereas stress-induced phosphorylation sites are highlighted in black.

© 2021 The Authors

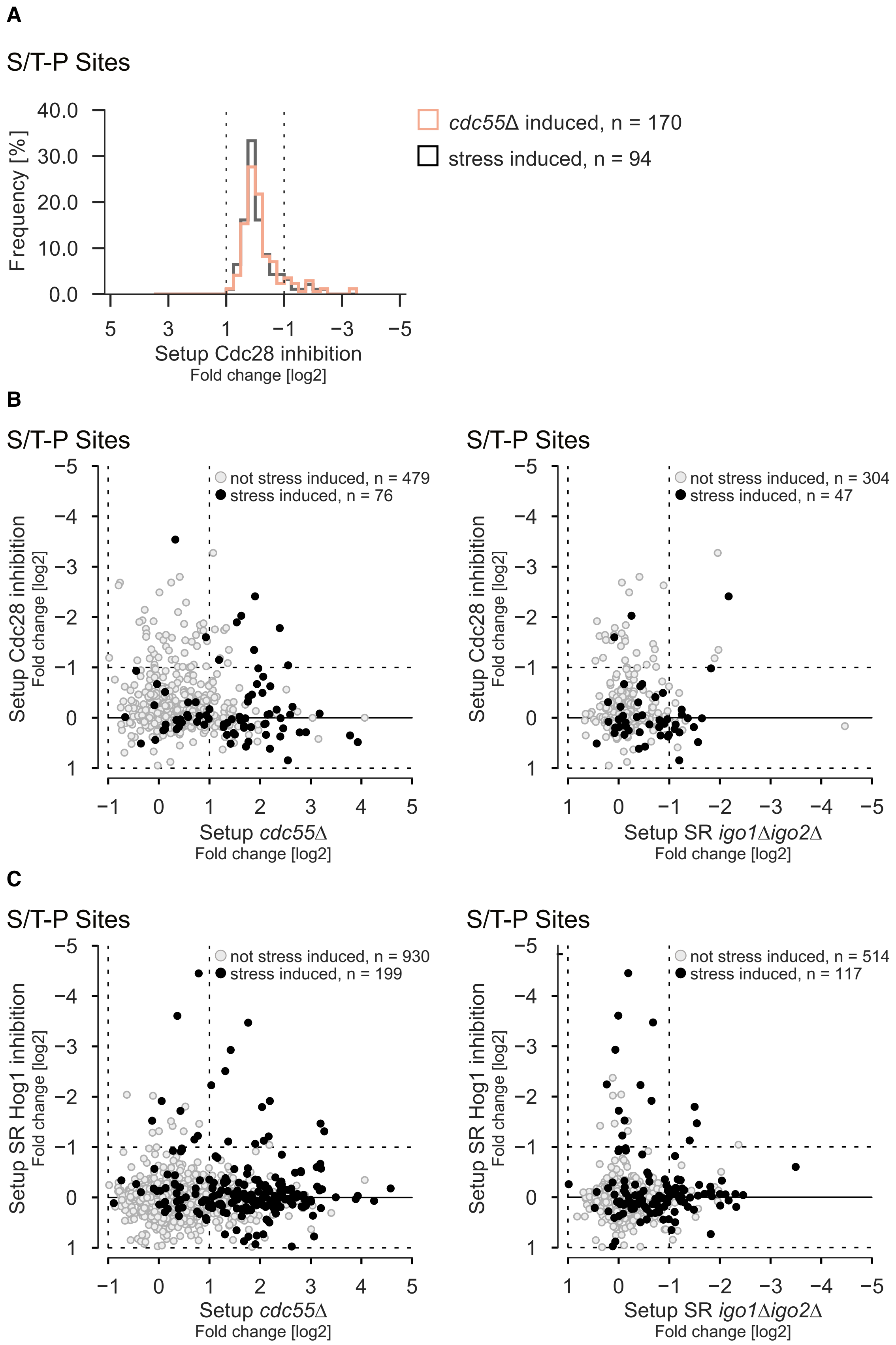

Figure EV3.

© 2021 The Authors

Figure EV4. Depletion of Cdc55 affects Gis1 and Rph1 phosphorylation patterns (related to Figs 4 and 5).

A Inducible Cdc55 depletion system shows effective degradation of Cdc55, which is not affected by hyperosmotic stress treatment. Cdc55-iAID-expressing cells were mock-treated (left) or treated with auxin and doxycycline (right) for 30 min followed by exposure to 0.5 M NaCl for times indicated.

B Cdc55 degradation increases *CTT1* and *PGM2* expression in absence of stress. Relative expression levels (fold over *IPP1*) of *CTT1* (left panel) and *PGM2* (right panel) in mock treated (gray dots) and auxin and doxycycline treated (orange dots) in Cdc55-iAID cells. Values are normalized relative to the highest value.

C Mobility shift assays (see Fig 5C) monitoring phosphorylation-induced mobility changes of Gis1 and Rph1 upon hyperosmotic stress in wild-type, *igo1*Δ*igo2*Δ-, and Cdc55-depleted cells. Two biological replicates are shown. Hyperosmotic stress treatment was controlled using an antibody directed against dual-phosphorylation of MAPK Hog1 (p38-p); Cdc55 protein levels were controlled using an anti-Cdc55 antibody. Cdc28: loading control.

© 2021 The Authors

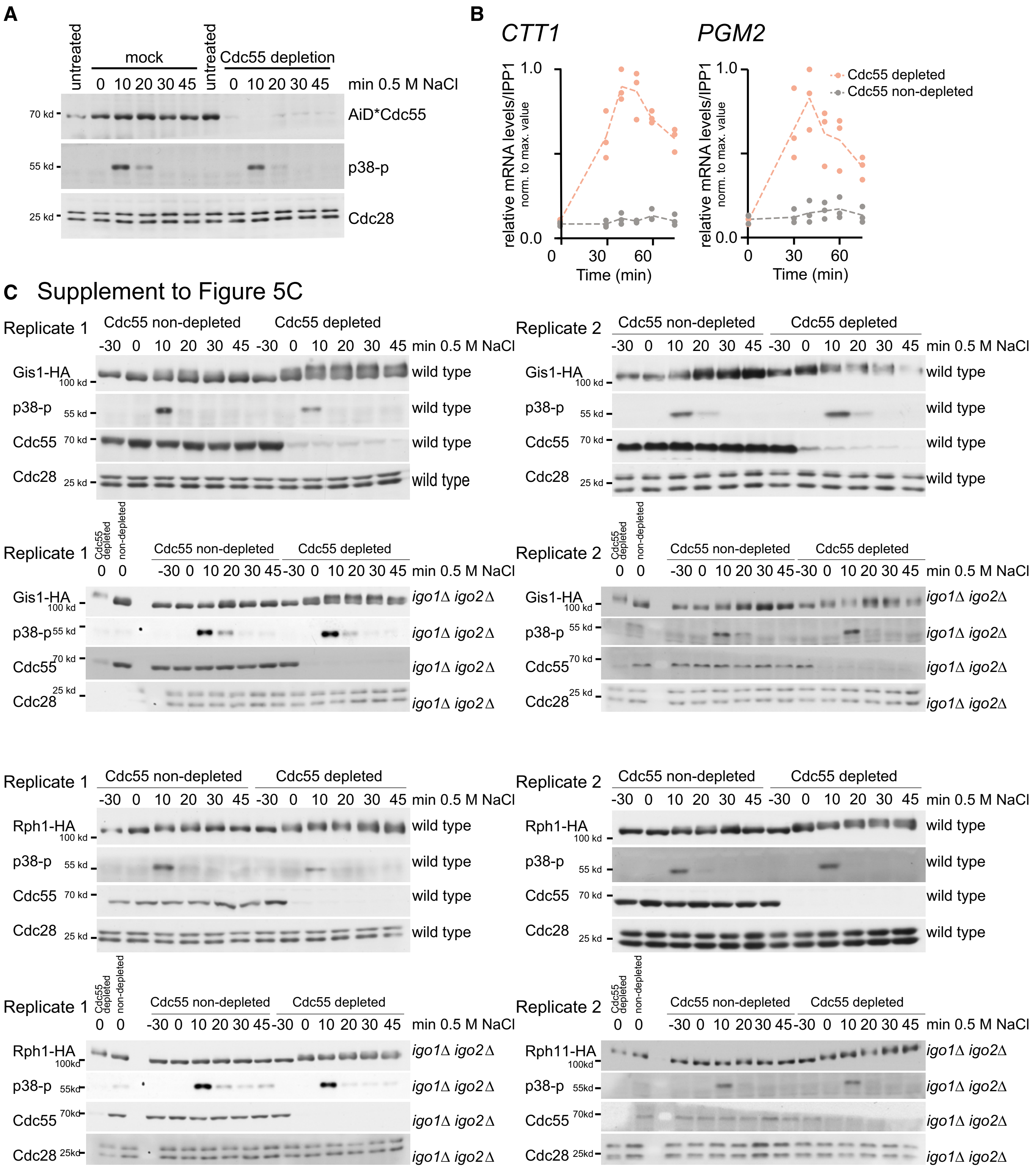

Figure EV4.

 © 2021 The Authors

Figure EV5. Reduced transcriptional activity of Gis1-5A and Rph1-5A mutants in response to stress (related to Fig 5C and D).

A Mobility shift assay of Gis1 and Rph1 (see Fig 5D) across several time points after Cdc55 depletion without exposure to hyperosmotic stress. Three biological replicates are shown. Cdc55 protein levels were controlled using an anti-Cdc55 antibody. Cdc28: loading control.

B Rim15 affects Gis1 and Rph1 phosphorylation patterns during hyperosmotic stress. Wild-type (+) or *rim15*Δ (Δ) cells expressing *GIS1*-HA (upper panel) or *RPH1*-HA (lower panel) were treated with 0.5 M NaCl for times indicated. Gel mobility shifts were visualized via Phos-tag gels (Phos-tagTM) using a 12CA5 antibody (HA). Activation of the high osmolarity glycerol (HOG) pathway (sign of effective salt stress treatment) was controlled with an anti-phospho-p38 antibody recognizing dual-phosphorylated Hog1. Pkg1: loading control.

C Rim15 indirectly affects phosphorylation of Gis1 and Rph1 via a Cdc55-dependent mechanism. *rim15*Δ (+) and *rim15*Δ*cdc55*Δ (Δ) cells expressing *GIS1*-HA (upper panel) or *RPH1*-HA (lower panel) were treated with 0.5 M NaCl for times indicated. Western blot experiments were similar as described in (B).

D Transcriptional kinetics of stress-induced expression of ESR genes *CTT1* (upper panel) and *PGM2* (lower panel) across indicated strains expressing point-mutated forms of Gis1 and Rph1 (related to 5F).

© 2021 The Authors

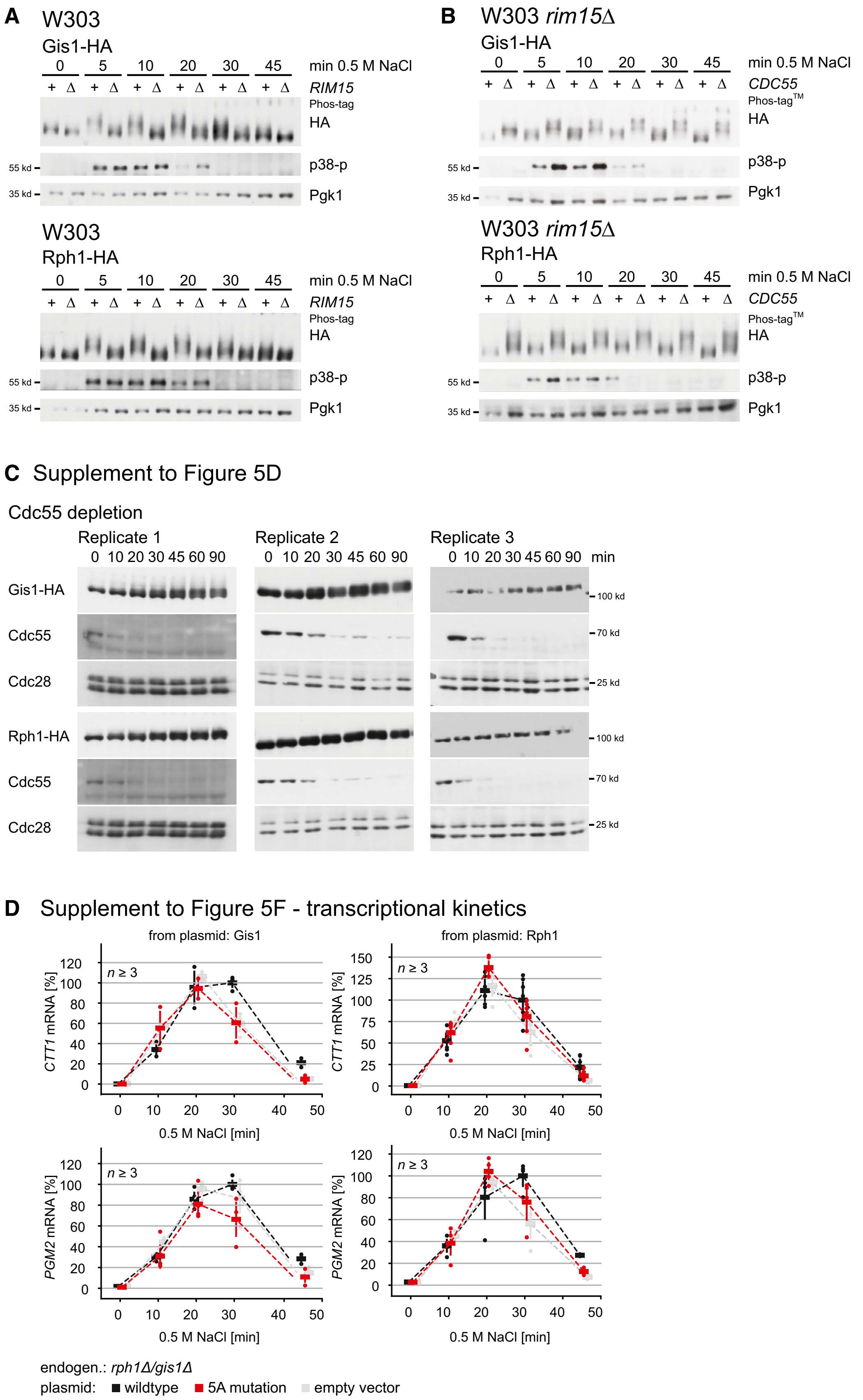

Figure EV5.

© 2021 The Authors
