# Supplementary material for: A phosphatase‐centric mechanism drives stress signaling response
Source: EMBO Rep. 2021 Sep 24;22(11):e52476. doi: 10.15252/embr.202152476 (PMC8567219; doi:10.15252/embr.202152476)
Supplement: Supplementary file 10 — Source Data for Appendix [file EMBR-22-e52476-s004.zip › embr202152476-sup-0010-SDataFigs.pdf]

Source data for Appendix Figure S2B

M-track assays - Series 1

antibody recognizing me3K9H3:

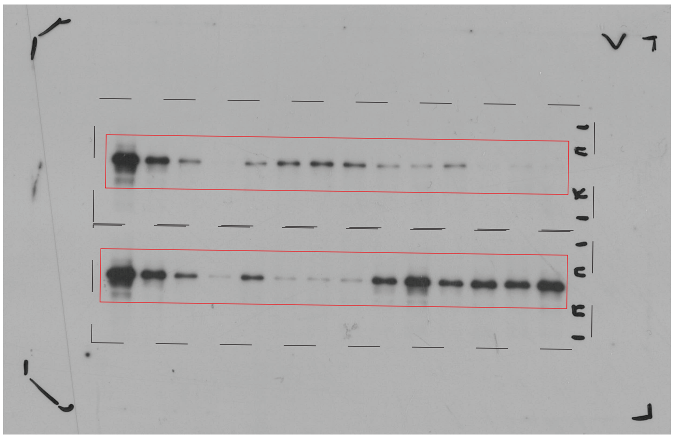

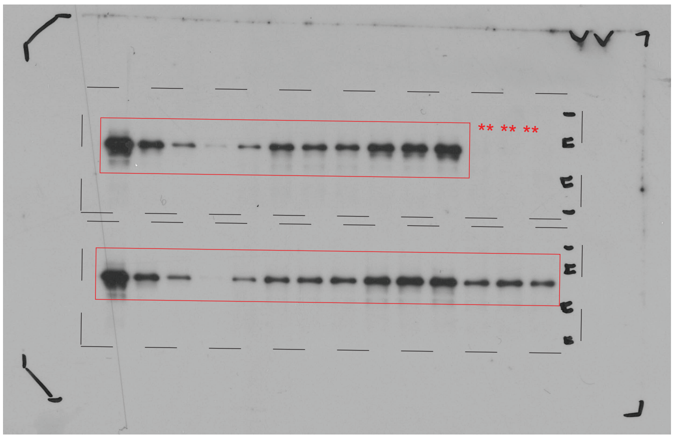

antibody recognizing HA: 12CA5

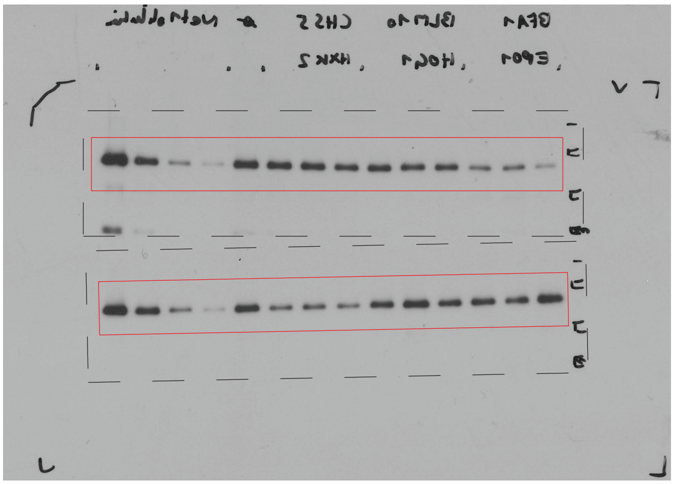

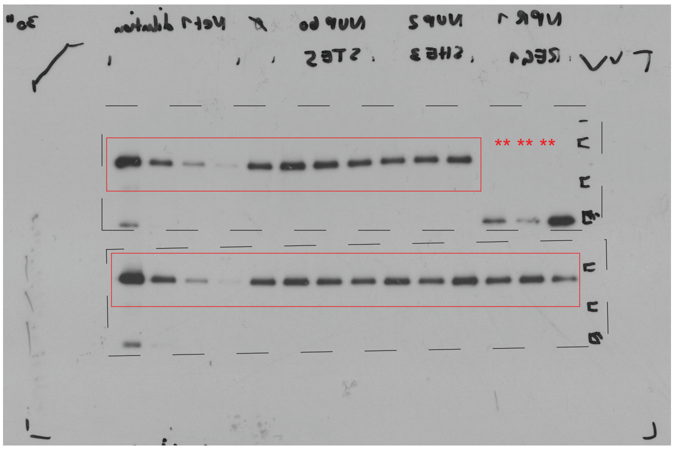

area shown in Appendix Figure S2

approximate edge of gel

* unused control

** unused strain

Source data for Appendix Figure S2B

M-track assays - Series 1

antibody recognizing me3K9H3:

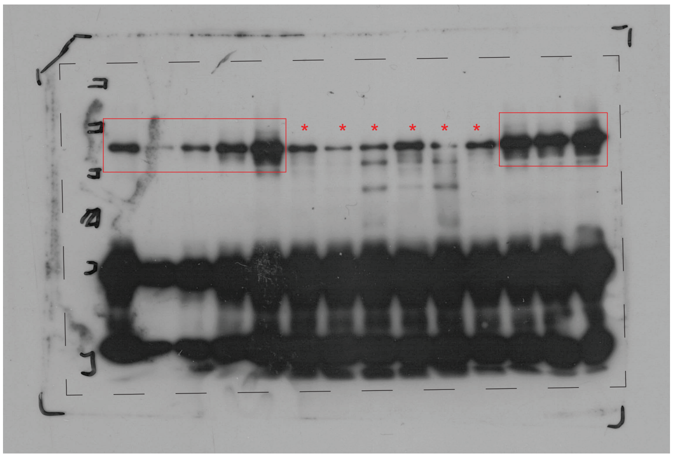

antibody recognizing HA: 12CA5

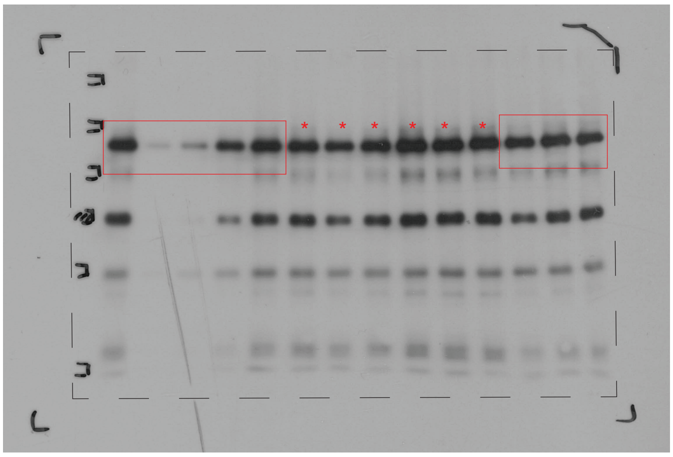

area shown in Appendix Figure S2

approximate edge of gel

* unused control

** unused strain

Source data for Appendix Figure S2B
M-track assays - Series 2

antibody recognizing me3K9H3:

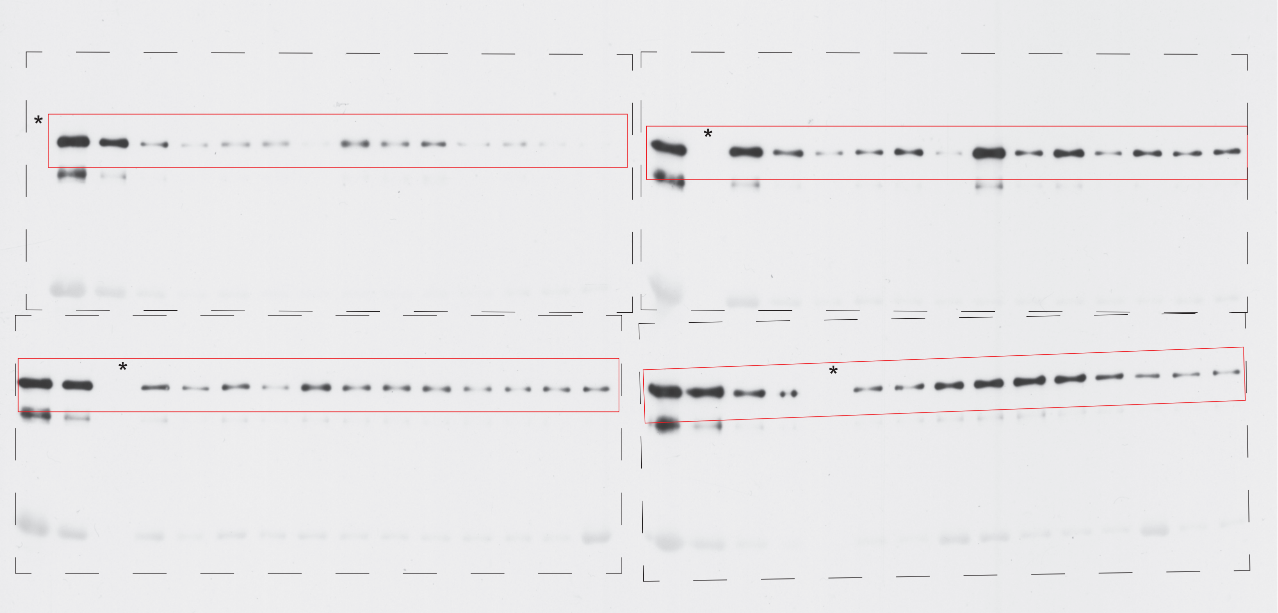

antibody recognizing HA: 12CA5

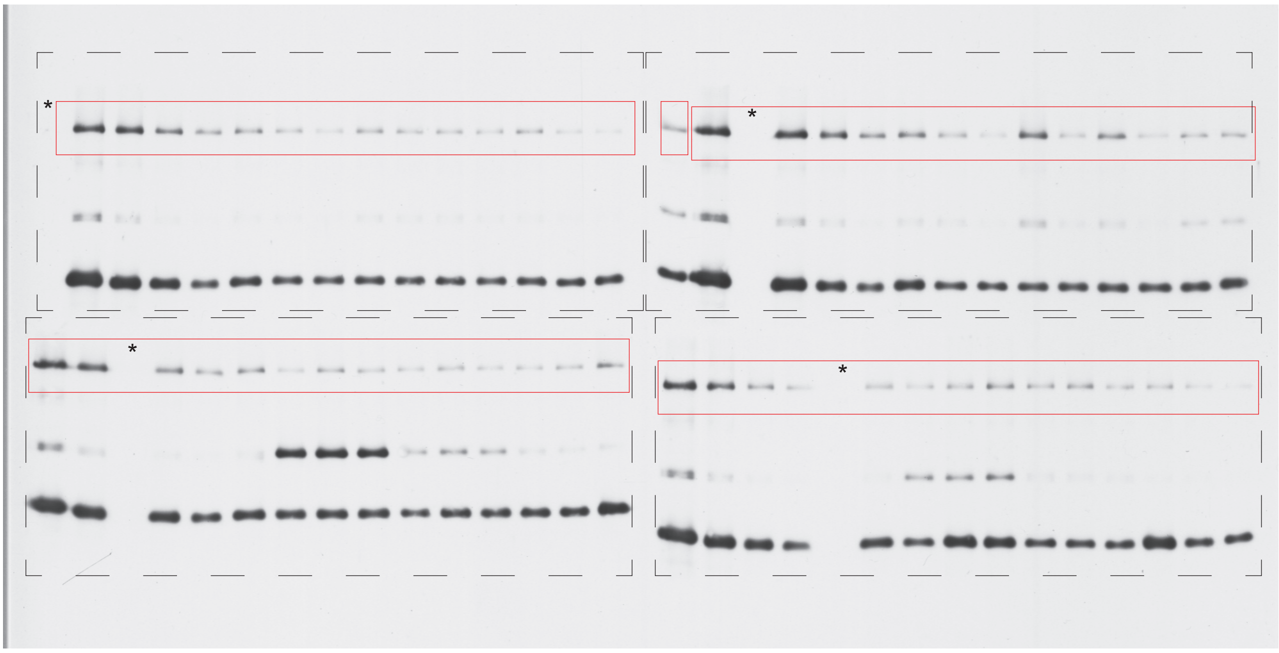

area shown in Appendix Figure S2
approximate edge of gel
* position of 130 kDa marker
