# Supplementary material for: A phosphatase‐centric mechanism drives stress signaling response
Source: EMBO Rep. 2021 Sep 24;22(11):e52476. doi: 10.15252/embr.202152476 (PMC8567219; doi:10.15252/embr.202152476)
Supplement: Supplementary file 11 [file EMBR-22-e52476-s007.pdf]

EMBO reports

A phosphatase-centric mechanism drives stress signaling response

David Hollenstein, Gabriela Gérecová, Natalie Romanov, Jessica Ferrari, Jiri Veis, Marion Janschitz, Reinhard Beyer, Christoph Schüller, Egon Ogris, Markus Hartl, Gustav Ammerer and Wolfgang Reiter,

DOI: 10.15252/embr.202152476

Corresponding author(s): Wolfgang Reiter (reiterw6@univie.ac.at)

Review Timeline:

Submission Date:	17th Jan 21
Editorial Decision:	9th Mar 21
Revision Received:	7th Jul 21
Editorial Decision:	19th Aug 21
Revision Received:	27th Aug 21
Accepted:	3rd Sep 21

Editor: Martina Rembold

Transaction Report:

(Note: With the exception of the correction of typographical or spelling errors that could be a source of ambiguity, letters and reports are not edited. Depending on transfer agreements, referee reports obtained elsewhere may or may not be included in this compilation. Referee reports are anonymous unless the Referee chooses to sign their reports.)

1st Editorial Decision **9th Mar 2021**

Dear Dr. Reiter

Thank you for the submission of your research manuscript to our journal. We have now received the full set of referee reports that is copied below.

As you will see, the referees acknowledge that the findings are potentially interesting. However, the referees also point out several technical concerns and have a number of suggestions how the study should be strengthened. In particular, it will be important to test whether the loss of PP2A-Cdc55 affects phosphorylation indirectly via a cell cycle arrest and to provide stronger links to high osmolarity sensitivity. Regarding the potential indirect effects of a cell cycle arrest, the referees suggested in their further feedback to me to monitor osmo-shock-induced changes in Rph1/Gis1 phosphorylation in freshly released G1 cells as one readout but also to perform phosphoproteomics of the osmotic stress response in fully arrested cells, as also suggested by referee 2. The latter experiment would certainly strengthen the conclusions significantly, since it allows a generalization of the results to all phosphorylation-sites.

Given these constructive comments, we would like to invite you to revise your manuscript with the understanding that the referee concerns (as detailed above and in their reports) must be fully addressed and their suggestions taken on board. Please address all referee concerns in a complete point-by-point response. Acceptance of the manuscript will depend on a positive outcome of a second round of review. It is EMBO reports policy to allow a single round of revision only and acceptance or rejection of the manuscript will therefore depend on the completeness of your responses included in the next, final version of the manuscript.

We invite you to submit your manuscript within three months of a request for revision. This would be June 9th in your case. However, we are aware of the fact that many laboratories are not fully functional due to COVID-19 related shutdowns and we have therefore extended the revision time for all research manuscripts under our scooping protection to allow for the extra time required to address essential experimental issues. Please contact us to discuss the time needed and the revisions further.

****
IMPORTANT NOTE: we perform an initial quality control of all revised manuscripts before re-review. Your manuscript will FAIL this control and the handling will be DELAYED if the following APPLIES:

1) A data availability section is missing.
2) Your manuscript contains error bars based on n=2. Please use scatter blots showing the individual datapoints in these cases. The use of statistical tests needs to be justified.

When submitting your revised manuscript, please carefully review the instructions that follow below. Failure to include requested items will delay the evaluation of your revision.
****

When submitting your revised manuscript, we will require:

1) a .docx formatted version of the manuscript text (including legends for main figures, EV figures and tables). Please make sure that the changes are highlighted to be clearly visible.

2) individual production quality figure files as .eps, .tif, .jpg (one file per figure).
Please download our Figure Preparation Guidelines (figure preparation pdf) from our Author Guidelines pages
https://www.embopress.org/page/journal/14693178/authorguide for more info on how to prepare your figures.

3) a .docx formatted letter INCLUDING the reviewers' reports and your detailed point-by-point responses to their comments. As part of the EMBO Press transparent editorial process, the point-by-point response is part of the Review Process File (RPF), which will be published alongside your paper.

4) a complete author checklist, which you can download from our author guidelines (). Please insert information in the checklist that is also reflected in the manuscript. The completed author checklist will also be part of the RPF.

5) Please note that all corresponding authors are required to supply an ORCID ID for their name upon submission of a revised manuscript (). Please find instructions on how to link your ORCID ID to your account in our manuscript tracking system in our Author guidelines
()

6) We replaced Supplementary Information with Expanded View (EV) Figures and Tables that are collapsible/expandable online. A maximum of 5 EV Figures can be typeset. EV Figures should be cited as 'Figure EV1, Figure EV2" etc... in the text and their respective legends should be included in the main text after the legends of regular figures.

- For the figures that you do NOT wish to display as Expanded View figures, they should be bundled together with their legends in a single PDF file called *Appendix*, which should start with a short Table of Content. Appendix figures should be referred to in the main text as: "Appendix Figure S1, Appendix Figure S2" etc. See detailed instructions regarding expanded view here:

- Additional Tables/Datasets should be labeled and referred to as Table EV1, Dataset EV1, etc. Legends have to be provided in a separate tab in case of .xls files. Alternatively, the legend can be supplied as a separate text file (README) and zipped together with the Table/Dataset file.

7) The accession numbers and database for the MS raw data should be listed in a formal "Data Availability " section (placed after Materials & Method) that follows the model below (see also <
https://www.embopress.org/page/journal/14693178/authorguide#dataavailability>). Please note that the Data Availability Section is restricted to new primary data that are part of this study.

Data availability

The datasets (and computer code) produced in this study are available in the following databases:

- [data type]: [name of the resource] [accession number/identifier/doi] ([URL or identifiers.org/DATABASE:ACCESSION])

*** Note - All links should resolve to a page where the data can be accessed. ***

8) We would also encourage you to include the source data for figure panels that show essential

data. Numerical data should be provided as individual .xls or .csv files (including a tab describing the data). For blots or microscopy, uncropped images should be submitted (using a zip archive if multiple images need to be supplied for one panel). Additional information on source data and instruction on how to label the files are available .

9) Our journal encourages inclusion of *data citations in the reference list* to directly cite datasets that were re-used and obtained from public databases. Data citations in the article text are distinct from normal bibliographical citations and should directly link to the database records from which the data can be accessed. In the main text, data citations are formatted as follows: "Data ref: Smith et al, 2001" or "Data ref: NCBI Sequence Read Archive PRJNA342805, 2017". In the Reference list, data citations must be labeled with "[DATASET]". A data reference must provide the database name, accession number/identifiers and a resolvable link to the landing page from which the data can be accessed at the end of the reference. Further instructions are available at .

10) Resource papers should include a structured Material and Methods section that includes a Reagents and Tools Table followed by a Methods and Protocols section. Please see the information on "Structured Methods" in our Guide to Authors
https://www.embopress.org/page/journal/14693178/authorguide#researcharticleguide

11) Regarding data quantification
The following points must be specified in each figure legend:
- the name of the statistical test used to generate error bars and P values,
- the number (n) of independent experiments (please specify technical or biological replicates) underlying each data point,
- the nature of the bars and error bars (s.d., s.e.m.)
Discussion of statistical methodology can be reported in the materials and methods section, but figure legends should contain a basic description of n, P and the test applied.
- Please also include scale bars in all microscopy images.

12) As part of the EMBO publication's Transparent Editorial Process, EMBO reports publishes online a Review Process File to accompany accepted manuscripts. This File will be published in conjunction with your paper and will include the referee reports, your point-by-point response and all pertinent correspondence relating to the manuscript.

You are able to opt out of this by letting the editorial office know (emboreports@embo.org). If you do opt out, the Review Process File link will point to the following statement: "No Review Process File is available with this article, as the authors have chosen not to make the review process public in this case."

We would also welcome the submission of cover suggestions, or motifs to be used by our Graphics Illustrator in designing a cover.

I look forward to seeing a revised version of your manuscript when it is ready. Please let me know if you have questions or comments regarding the revision.

Yours sincerely

Martina Rembold, PhD
Senior Editor
EMBO reports

****

Referee #1:

This manuscript analysis the stress response in yeast focusing on the role of the PP2A protein phosphatase and its regulatory subunits Cdc55 and Rts1. Using a range of genetic manipulations and MS based quantitative mass spectrometry approaches they uncover that PP2A-Cdc55 at steady state dephosphorylates numerous proteins of the stress response. Upon stress the PP2A-Cdc55 phosphatase is inhibited through the Rim15-Igo1/2 pathway allowing robust increase in phosphorylations without having to increase the activity of the major stress kinases. They further investigate the mechanism by showing that PP2A-Cdc55 regulates the transcriptional activity of Gis1 and Rph1 by modulating their phosphorylation pattern.
Overall, this is a thorough study drawing robust and interesting conclusions. The clean genetic approaches and quantitative mass spectrometry is a powerful approach to look at PP2A in stress signaling. The manuscript is well written and easy to follow even for a person not working on yeast and the figures are nice and understandable. I highly recommend publication in EMBO Reports and only have minor suggestions.

1) It is unclear what evidence the authors have for excluding simultaneous activation of stress kinases and inhibition of PP2A-Cdc55? Maybe I missed this point. It would not in my view change the interesting concept of the paper.
2) Is there anything in their data that would show how the Rim15-Igo1/2 pathway gets activated upon stress? Is this through an increase in kinase activity activating Rim15?
3) We recently showed that human PP2A-B55 (corresponding to PP2A-Cdc55) is able to act on proline directed sites while PP2A-B56 (corresponding to PP2A-Rts1) is very inefficient. This fits well with their observations described here so I would suggest to cite this since they already site work on PP2A-B55 from the human system.

Referee #2:

Review for "A phosphatase-centric mechanism drives stress signaling response"
Summary
In this manuscript, Hollenstein et al. investigate the role of protein phosphatase 2A bound to regulatory subunit Cdc55 (PP2ACdc55) as a main downstream effector in the Saccharomyces cerevisiae hyperosmotic stress response signaling pathway. Combining quantitative mass spectrometry (MS) experiments with molecular and biochemical approaches, the authors identify a role for PP2ACdc55 in regulating the hyperosmotic stress response via dephosphorylation of downstream substrates. Using several phosphoproteomic approaches, they demonstrate that nearly 1/3 of the stress-induced phosphorylome is under the control of PP2ACdc55. They also show that PP2ACdc55 can directly target stress-related proteins, many of which are associated with gene expression regulation. By focusing on the transcription factors Gis1 and Rph1, the authors demonstrate that PP2ACdc55 directly regulates the activity of these proteins to control the transcriptional response under hyperosmotic stress. These results guide them to a proposed model in which phosphatase inhibition, rather than increased kinase activity, induces a positive phosphorylation response to osmotic stress.
Overall, the experiments in this manuscript are elegantly executed, and the authors provide

potentially interesting new insights into the roles of PP2A in regulating cellular stress responses. A few important concerns are noted. The authors need to rule out any potential indirect effects of cell cycle arrest in the PP2ACdc55-regulated phosphorylome (see major point #1 below). Also, the manuscript lacks clear experimental evidence that more definitively link their findings of the PP2ACdc55-regulated signaling events to the high osmolarity sensitivity phenotype observed by lack of Cdc55 (major point #2 below). Some experiments are also included with questionable comparability to other provided evidence due to different genotypes and should be addressed.

Major Comments
1. Potential indirect effects of cell cycle arrest in the PP2ACdc55-regulated phosphorylome. PP2A plays key roles in mitotic progression, both during entry and exit of mitosis. It was previously reported that the cdc55Δ cells have a pronounced delay in G2/M as determined by flow cytometry (Yellman and Burke, MBOC, 2006: https://doi.org/10.1091/mbc.e05-04-0336). It was also previously shown that cells lacking PP2ACdc55 show gross morphological defects and poor growth due to Cdk inhibitory tyrosine kinase Swe1 activation (Godfrey et al., 2017, Wang and Burke, 1997). Moreover, cells lacking Cdc55 also show strong defect in mitotic exit as shown for example in Touati et al., 2019 (https://doi.org/10.1016/j.celrep.2019.10.041). Therefore, there is a major concern with the possibility that cell cycle effects are leading to confounding data interpretation. The authors find a range of S/T-P sites going up in cdc55delta. But since Cdc28 (CDK) is known to phosphorylate S/T-P sites, and mediate a wave of CDK phosphorylation in G2/M-M phases, one possibility is that the cdc55 mutant is partially arrested at G2/M or M-phases, and that most sites going up in the mutant are actually Cdc28 substrates regulated during G2/M and/or M phases.
1a. The authors need to monitor cell cycle distribution in wt and in cdc55delta to make sure the changes are not due to changes in cell cycle. Even in the case of a fast 30 min depletion of Cdc55, that could be enough time to trap several cells in G2/M or M phases an up-regulate S/T-P sites. It is essential that the authors rigorously test this possibility by monitoring cell cycle distribution in the cells and conditions used for generating their phosphoproteomic analysis.
2b. The issue of potential cell cycle effects is a serious concern as it could imply that the authors are revealing the mitotic action of Cdc55, rather than its role in the osmotic stress response. Along those lines, it is concerning that the authors did not cite Touati et al, 2019. This work already performed phosphoproteomic analysis of cells lacking Cdc55 in the context of mitosis. How similar are the set of targets identified by that work compared to the present paper? Also, does the condition of osmotic stress used by the authors result in any cell cycle arrest on its own? This could also result in confounding data interpretation and needs to be carefully checked.

2. The manuscript lacks clear experimental evidence that more definitively link their findings of PP2ACdc55-regulated signaling events to the high osmolarity sensitivity phenotype observed by lack of Cdc55:
2a. It is surprising that there is no rescue effect in the igo1Δigo2Δcdc55Δ triple mutant compared with igo1Δigo2Δ, especially considering that Cdc55 depletion can rescue the mRNA level of two ESR genes to WT levels in Figure 4D & E. As this paper suggests that PP2ACdc55 inactivation upon stress is important for proper phosphorylation, it is somewhat confusing that cdc55Δ cells show such a strong stress sensitivity to osmotic treatment, but they grow normally under untreated condition. Does Cdc55-AID also show the same sensitivity to osmotic stress? Can a transient depletion of Cdc55 provide at least partial rescue of osmotic sensitivity in igo1Δigo2Δ cells?
2b. Do Gis1-5A and Rph1-5A cells show sensitivity to osmotic stress? How about gis1Δrph1Δ cells? It may be necessary to show a sensitivity assay when modulating these downstream targets. One may expect Gis1-5D and Rph1-5D to show resistance to osmotic stress. The authors should test this.

Other Comments:

1. The M-track experiment to test for in vivo interaction of PP2ACdc55 with Gis1 and Rph1 involves using a rim15Δ to prevent inhibition by Igo1/2, whereas the phosphoproteome experiment immediately preceding this simply used an igo1/2Δ. It is currently unclear why two different approaches were used to prevent inhibition by Igo1/2, and it raises concerns as to the compatibility of the phosphoproteomic data and the M-track results.
2. Can the authors speculate on why stress- and cdc55Δ-induced phosphorylation sites showed a trend towards higher phosphorylation in setup SR cdc55Δ (Figure 2E) since kinase activity is not changed upon stress suggested by the model?
3. All targets in Sup. Fig 3 have 3 loadings. Do all of them follow a series of dilution or just 3 replicates with same loading? It's better to clarify this in figure legends.
4. The color scheme of Figure 4E makes it hard to discern exactly where the bars end. Solid coloration rather than gradient might serve this plot better.
5. In Figure 5A, is there any reason not to choose setup SR and setup cdc55Δ?
6. It may be advisable to also show the quantification and Q-value for Gis1 and Rph1, like in Figure 3D.
7. There should be a loading control in Figure 5D, as phosphorylation of Rph1-HA is not obvious. Instead, it looks like there is more protein at later time points.
8. The method of phosphorylation site deconvolution used to determine which sites on Gis1 and Rph1 are affected by PP2ACdc55 is not terribly intuitive as described. The authors should clarify how the deconvoluted sites can be definitively attributed to PP2ACdc55 amongst all the regulatory changes occurring during the hyperosmotic stress response.

Referee #3:

To assess the roles that PP2A complexes (i.e. those built with Cdc55 vs. Rts1 B regulatory subunits) play in coordination of the response to hyperosmotic shock, Hollenstein et al. performed a series of SILAC-based quantitative phosphoproteomics MS experiments. Specifically, they assess the effects of different mutant backgrounds on the osmoshock-induced changes in the phosphoproteome. Data for these analyses comes not only from this present work but also previously published works including an online BioRiv preprint from the group.
The group found a high degree of overlap between sites affected by osmotic shock and CDC55 or RTS1 deletion and conclude both that PP2A serves to oppose or contribute to the osmotic shock response, and that Cdc55 and Rts1 complexes have largely distinct effectors/substrates. Interestingly, the authors conclude that Rts1 counteracts a portion of Hog1-dependent signaling while Cdc55 affects largely Hog1-independent osmoshock signaling.
The authors then focus on the endosulfines Igo1/2 which are transiently hyperphosphorylated upon osmoshock suggesting a mechanism of osmo-shock induced inhibition of PP2A. Indeed, they discovered that the majority of Cdc55- and stress-dependent phosphosites are misregulated in igo1/2Δ cells whereas loss of igos has no impact on Hog1-dependent phosphosites. From these observations the authors conclude that inhibition of PP2A-Cdc55 is mechanistically responsible for approximately 1/3 of the phosphoproteomic response to osmotic shock which is comparable in magnitude to the regulation independently played by the classical Hog1 MAPK pathway. Subsequent analyses demonstrate that PP2A-Cdc55 affects about 1/2 of all osmoshock regulated S/T-P motifs, whereas only 12% are regulated by Hog1-dependent signals. An M-Track protein-

protein proximity assay was then used to identify/confirm putative direct PP2A-Cdc55 substrates. Next, the authors engineered a CDC55-depletion system to assess the role of this PP2A-Cdc55 in the transcriptional response to osmoshock. This line of investigation demonstrated that Igo-dependent inhibition of PP2A-Cdc55 is required for full induction of ESR genes. The relevant signaling was then shown to be mediated through Gis1 and Rph1 transcription factors, which, according to M-Track experiments, are likely direct substrates of PP2A-Cdc55.
Globally, this manuscript presents a robust dataset that reveals a complete and interesting narrative. Although the concept that PP2A-Cdc55 represents a key regulatory node in the cellular response to osmotic shock signaling is appreciated, the novelty of a phosphatase (as opposed to a kinase) being regulated as a mediator of signal transduction is rather oversold.
Major comments
1. The authors should dial back their enthusiasm of discovering that PPase regulation is a novel modus operandi in signal transduction. Indeed, analogous PPase regulation is well documented for PP1 (downstream of glucose) as well as PP2B/calcineurin (downstream of Ca2+). The authors should acknowledge this prior literature and put their results into proper context.
Minor comments
1. I do not understand the logic of this sentence:
"We next focused on the role of PP2ACdc55 in the transcriptional regulation in response to hyperosmotic stress. Previously we observed a reduced transcriptional response to hyperosmotic stress in cdc55Δ cells (Reiter et al., 2013), which is in contrast to the finding that inhibition of PP2ACdc55 is part of the stress response."

If a component of the relevant signaling pathway is missing, would one not expect the response to be muted?
2. When introducing Igo1/Igo2 as specific inhibitors of PP2A, I would explicitly mention that they act by binding to PP2A. This is of course logical but may not be obvious to every reader.
3. Fig. 5E: Please indicate what samples were analyzed in the last two lanes of these blots. Also, normal SDS-PAGE gels could be useful here to assess if the 5A-mutant proteins are really more abundant or rather just transfer more efficiently out of the Phos-tag gels.
4. Fig. 5F, Suppl. Fig. 5D: The authors claim that they see decreased levels of CTT1 and PGM2 mRNA expression upon expressing the 5A mutants of Gis1 and Rph1. These results could be better described explaining that this difference is only apparent at later time points and that induction at earlier time points appears to be independent of these transcription factors.

POINT-BY-POINT REPLY: EDITOR

Dear Dr. Reiter

Thank you for the submission of your research manuscript to our journal. We have now received the full set of referee reports that is copied below.

As you will see, the referees acknowledge that the findings are potentially interesting. However, the referees also point out several technical concerns and have a number of suggestions how the study should be strengthened. In particular, it will be important to test whether the loss of PP2A-Cdc55 affects phosphorylation indirectly via a cell cycle arrest and to provide stronger links to high osmolarity sensitivity. Regarding the potential indirect effects of a cell cycle arrest, the referees suggested in their further feedback to me to monitor osmo-shock-induced changes in Rph1/Gis1 phosphorylation in freshly released G1 cells as one readout but also to perform phosphoproteomics of the osmotic stress response in fully arrested cells, as also suggested by referee 2. The latter experiment would certainly strengthen the conclusions significantly, since it allows a generalization of the results to all phosphorylation-sites.

Given these constructive comments, we would like to invite you to revise your manuscript with the understanding that the referee concerns (as detailed above and in their reports) must be fully addressed and their suggestions taken on board. Please address all referee concerns in a complete point-by-point response. Acceptance of the manuscript will depend on a positive outcome of a second round of review. It is EMBO reports policy to allow a single round of revision only and acceptance or rejection of the manuscript will therefore depend on the completeness of your responses included in the next, final version of the manuscript.

We invite you to submit your manuscript within three months of a request for revision. This would be June 9th in your case. However, we are aware of the fact that many laboratories are not fully functional due to COVID-19 related shutdowns and we have therefore extended the revision time for all research manuscripts under our scooping protection to allow for the extra time required to address essential experimental issues. Please contact us to discuss the time needed and the revisions further.

We thank the editor and referees for their insightful comments, which helped us to substantially improve our manuscript. As explained in detail below, we have addressed all points and added new validation data, supporting our findings. All changes to the manuscript have been highlighted in red.
Importantly, we have carefully considered the possibility that deletion of *CDC55* causes cells to accumulate in G2/M phase due to a defect in mitotic exit, which could potentially confound our data interpretation. We have accumulated several lines of evidence that show that no such cell cycle effect occurs in the studied conditions. This is both demonstrated experimentally by FACS (**1**), computationally by overlapping our phospho-proteomic data with cell cycle related datasets in yeast (**2**), and by comparing the effect of Cdk1 inhibition with our datasets (**3**). We hope that the provided experimental and computational analyses convince the editor and the referees that cell cycle effects do not occur

in the studied conditions and therefore do not confound our data analysis or interpretation.

(**1**) As discussed with the editor and following the suggestions of the referees, we have measured FACS profiles of *cdc55*Δ and Cdc55 depletion mutants. The profiles cannot be distinguished from the wildtype FACS profiles, and hyperosmotic stress conditions also do not elicit any significant changes. We can therefore assume that no cell cycle related effects occur as a consequence of any of the analyzed conditions. The FACS profiles have been included in the updated manuscript as **Figure EV1E**, and are described in the Results section in context of a necessary quality control. In addition, we have observed no obvious change in abundance of several cell-cycle marker proteins that would point to an enrichment of one particular cell-cycle phase in *cdc55*Δ and Cdc55 depletion mutants. We now present the protein SILAC ratios in **Figure EV1F**. We have changed the Results accordingly:

"[...] Given that PP2A^{Cdc55} is known to play a role in regulating cell cycle progression (Godfrey et al., 2017; Moyano-Rodriguez and Queralt, 2020; Touati et al., 2019), we tested whether deletion of *CDC55* and our experimental stress conditions could lead to a cell cycle arrest or delayed cell cycle progression, which would indirectly result in the observed changes to the phosphorylome. We used FACS to measure DNA content profiles of the wildtype and knockout strains exposed to no stress, and five and ten minutes of hyperosmotic stress. No significant differences in the distribution of cells could be identified (**Figure EV1E**). We also did not observe any particular abundance changes in well-characterized cell cycle markers covered in our dataset (**Figure EV1F)** (Kelliher et al., 2018). These observations refute the possibility that the increase in phosphorylation in the *cdc55*Δ strain can be attributed to cell cycle effects. [...]"

When introducing the Cdc55 depletion experiment we added the following sentence:

"[...] Notably, the depletion of Cdc55 did not elicit any significant cell cycle effects under the experimental conditions used (Figure EV1E). [...]"

(**2**) According to the suggestion of Referee 2, we have looked into the similarity of the Cdc55 target sets as defined by our study in comparison to Touati *et al*. (Touati et al., 2019). Touati *et al*. defined phosphorylation sites with dynamic behavior during progression through mitosis, along with static sites that do not change during mitosis. In addition, 40 putative Cdc55 target sites - which show decreased phosphorylation during progression through mitosis in wild type - have been defined.

From the 40 putative Cdc55 target sites only 10 were also covered in our *cdc55*Δ, Cdc55 depletion and stress response data sets (see **Figure A** for the Referee's attention below). Three of those phosphorylation sites were increased

in *cdc55*Δ and upon hyperosmotic stress. Only two showed increased phosphorylation upon Cdc55 depletion, one of them being affected by hyperosmotic stress. These results could indicate that the set of phosphorylation sites defined in Touati *et al*. is primarily regulated during progression through mitosis and is, for the most part, not involved in the hyperosmotic stress response. Another possibility is that several of these sites are merely indirectly affected by the deletion of *CDC55*, as they behave differently in the Cdc55 depletion experiment. However, given the very small number of overlapping sites, we do not believe that drawing any conclusions is justifiable and have thus not included this comparison into the manuscript.

We next selected phosphorylation sites with dynamic and static behaviour in the wild type as defined by Touati *et al.*, and looked at the distribution of the respective SILAC fold-changes in our *cdc55*Δ dataset. There was no difference in the Cdc55-dependence between the static and the dynamic group of phosphorylation sites (see **Figure B** for the Referee's attention below). Moreover, the majority of dynamic sites (75%) was not affected by the deletion of *CDC55*, confirming that in the experimental conditions used in our study the *cdc55*Δ deletion does not cause broad cell-cycle effects.

To support this finding, we performed a similar analysis using the phospho-proteomic dataset of Godfrey *et al*. (Godfrey et al., 2017) that defined sites that become increasingly phosphorylated during the transition from G1 to mitosis. Here the results are similar to the above analysis - there is no obvious difference in the distribution of SILAC fold changes between sites that are static or dynamic during the cell cycle (see **Figure C** for the Referee's attention below).

We believe that the other experiments sufficiently exclude the possibility of a potential confounding effect of the cell cycle. These additional analyses (**Figures B and C** below) do not significantly contribute to that argument, and were thus not included in the final manuscript as we feel that this might be out of scope.

Figure for the Referee's attention:

A

Putative Cdc55 target proteins defined in Touati et al., 2019

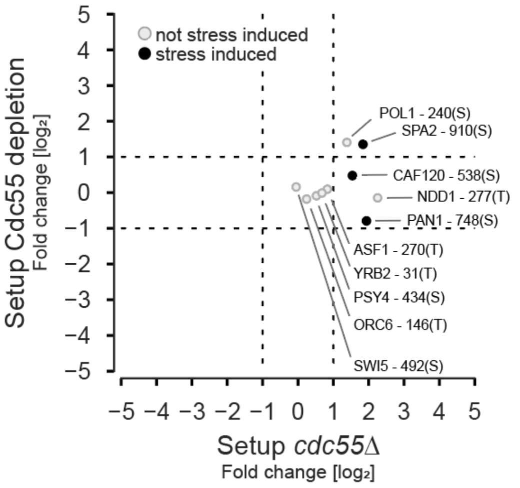

B

Comparison to Touati et al., 2019

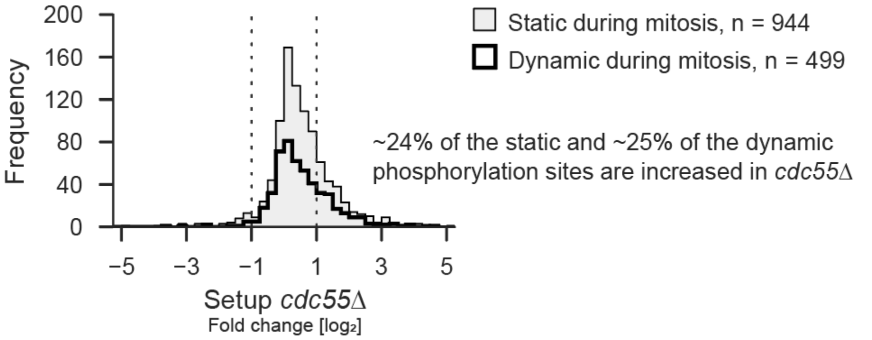

C

Comparison to Godfrey et al., 2017

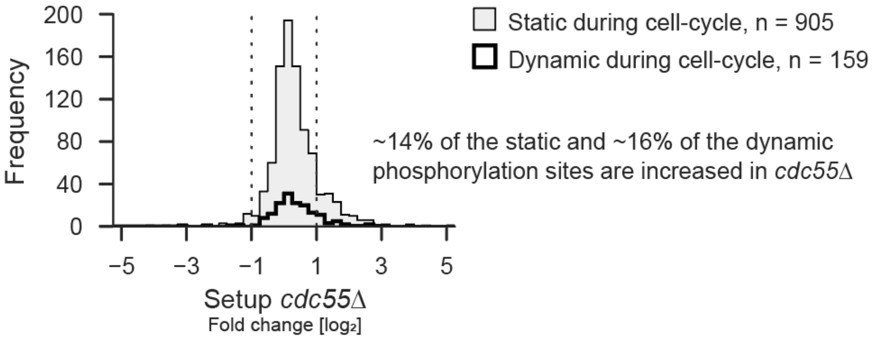

(3) Finally, we also checked whether the stress-dependent phosphorylation sites that become increased upon Cdc55 removal and hyperosmotic stress exposure could potentially be Cdk1 (Cdc28) targets as well. To that end we compared the respective target set from our phospho-proteomic study with the Cdc28 substrates derived from the MS dataset from Kanshin *et al*. (Kanshin et al., 2017). Out of the 49 stress- and Cdc55-dependent S/T-P sites that were covered in the Cdc28 dataset, only 7 appeared to be also affected by inhibition of the CDK. Moreover, we found that the key regulatory site of Cdc28, Tyr19, increased in phosphorylation upon deletion or depletion of Cdc55 (~2.7 fold and ~2.2 fold, respectively). Given that phosphorylation of this site inhibits the activity of the CDK, it becomes even less likely that the observed increase of phosphorylation by removal of Cdc55 is indirectly caused by elevated Cdc28 activity. This analysis does raise an interesting point, however, as it effectively demonstrates that inhibition of PP2A^{Cdc55} affects a distinct set of S/T-P sites during the hyperosmotic stress response. We now point this out in the Results (the analysis has been included into **Figure EV3**):

"[...] PP2A^{Cdc55} preferentially dephosphorylates phospho-threonines in threonine-proline (TP) motifs (Agostinis et al., 1990; Cundell et al., 2016; Godfrey et al., 2017; Kruse et al., 2020). To examine whether the proteins involved in those biological processes might be directly dephosphorylated by PP2A^{Cdc55} we first performed a motif enrichment analysis using MotifX (Schwartz and Gygi, 2005). We found the serine-proline (SP), threonine-proline (TP) to be significantly enriched (FDR < 1%) (Figure 3B), with ~60% of sites containing the motif. Moreover, the TP motif was significantly enriched within stress-induced S/T-P motifs affected by inhibition of PP2A^{Cdc55} (enrichment p-value < 0.001, Figure 3C). S/T-P is the substrate motif of MAPKs and cyclin-dependent kinases (CDK). PP2A^{Cdc55} counteracts global CDK phosphorylation in the context of cell cycle regulation and it has been suggested that PP2A^{Cdc55} directly dephosphorylates substrates of Cdc28 - the homolog of mammalian Cdk1 and the main CDK driving the cell cycle in yeast (Godfrey et al., 2017, Mendenhall and Hodge, 1998). To investigate whether stress- and Cdc55-dependent S/T-P sites correspond to Cdc28 substrates, we integrated our phospho-proteomic data with a published dataset analyzing the immediate impact of Cdc28 inhibition on the phospho-proteome (Kanshin et al., 2017) (Figure 1). The majority of S/T-P sites induced by hyperosmotic stress or deletion of *CDC55* did not display any change in abundance upon inhibition of Cdc28 (Figure EV3A and EV3B). In detail, of the 49 stress- and Cdc55-dependent S/T-P sites only 7 (~14%) displayed Cdc28 dependence. Vice versa, the deletion of *CDC55* also had no significant impact on S/T-P sites affected by inhibition of Cdc28 (Figure EV3B). Similar results were obtained when comparing the setup SR *igo1Δigo2Δ* with the Cdc28 inhibition data set (Figure EV3B). We also could confirm that the vast majority (>90%) of stress- and Cdc55-dependent S/T-P sites are independent of the MAPK Hog1 (Figure EV3C). These data demonstrate that PP2A^{Cdc55} targets a unique set of mainly Cdk1 independent phosphorylation sites as part of the hyperosmotic stress response. Moreover, inhibition of PP2A^{Cdc55} appears to strongly regulate one third, and affect more than half of all stress induced S/T-P motifs, in contrast to Hog1, which targets about 11% of these motifs (Table EV2). ~~PP2A^{Cdc55} strongly regulates one third, and affects more than half of all stress induced S/T-P motifs, in contrast to the MAPK Hog1, which targets about 12% of these motifs (Supplementary Table 2).~~

We next analyzed the ability of Cdc55 to interact with putative substrates *in vivo* using the M-Track protein-protein proximity assay [...]"

In addition, we have updated the corresponding paragraph in the Discussion:

"[...] It is also well-known that PP2A^{Cdc55} antagonizes Cdk1 phosphorylation during the cell cycle (Touati et al., 2019, Godfrey et al., 2017; Mochida et al., 2009). However, we find that inhibition of Cdc28 does not affect stress-induced S/T-P phosphorylation sites and that PP2A^{Cdc55} acts on a complementary set of S/T-P sites during hyperosmotic stress (Figure EV3A and EV3B). ~~It is also well-known that PP2A^{Cdc55} dephosphorylates Cdk1 substrates during the cell cycle (Godfrey et al., 2017; Mochida et al., 2009). However, activity of Cdc28, the main CDK in yeast, becomes repressed in response to stress exposure (Kanshin et al., 2015b); thus PP2A^{Cdc55} affects a complementary set of S/T-P sites during hyperosmotic stress.~~ [...]"

POINT-BY-POINT REPLY: Referee #1

This manuscript analysis the stress response in yeast focusing on the role of the PP2A protein phosphatase and its regulatory subunits Cdc55 and Rts1. Using a range of genetic manipulations and MS based quantitative mass spectrometry approaches they uncover that PP2A-Cdc55 at steady state dephosphorylates numerous proteins of the stress response. Upon stress the PP2A-Cdc55 phosphatase is inhibited through the Rim15-Igo1/2 pathway allowing robust increase in phosphorylations without having to increase the activity of the major stress kinases. They further investigate the mechanism by showing that PP2A-Cdc55 regulates the transcriptional activity of Gis1 and Rph1 by modulating their phosphorylation pattern.
Overall, this is a thorough study drawing robust and interesting conclusions. The clean genetic approaches and quantitative mass spectrometry is a powerful approach to look at PP2A in stress signaling. The manuscript is well written and easy to follow even for a person not working on yeast and the figures are nice and understandable. I highly recommend publication in EMBO Reports and only have minor suggestions.

We thank the referee for appreciating the quality of our work, as well as acknowledging the interesting conclusions that we draw from it.

1) It is unclear what evidence the authors have for excluding simultaneous activation of stress kinases and inhibition of PP2A-Cdc55? Maybe I missed this point. It would not in my view change the interesting concept of the paper.

We thank the referee for raising this point. Our reasoning regarding potential simultaneous activation of $PP2A^{Cdc55}$ counter kinases was as follows: For one thing, the kinase or kinases in question must become induced immediately after stress exposure and show a higher activity after 5 min stress exposure. Regardless of whether hyperosmotic stress is present or absent, our results indicate that inhibition of $PP2A^{Cdc55}$ is sufficient to induce phosphorylation of specific S/T-P sites. The putatively activated kinase(s) would therefore need to be downstream of $PP2A^{Cdc55}$ and not upstream of the phosphatase.

The proline-containing phosphorylation motifs SP and TP are indicative of a subclass of serine/threonine specific kinases, such as MAPKs and CDKs. However, MAPKs either target only a small subset of stress-induced S/T-P phosphorylation sites (Hog1 and Kss1 (Janschitz et al., 2019; Romanov et al., 2017), are not activated during the immediate stress response (Fus3 and Slt2 (García-Rodríguez et al., 2005; Hao et al., 2008)) or are not expressed in haploid cells (Smk1 (Pierce et al., 1998)). Importantly, over 90% of stress- and $PP2A^{Cdc55}$-dependent S/T-P motifs were found to be independent of the MAPK Hog1 and deletion of Kss1 has been described to not affect Hog1-independent stress-induced S/T-P sites. Activity of the CDK Cdc28 becomes repressed upon deletion or depletion of Cdc55 because of increased phosphorylation of the inhibitory Try19 site [our MS data and (Minshull et al., 1996)] and, as we now have included in the manuscript, the CDK targets a different set of S/T-P sites. Pho85, another CDK, has been described to mediate hyperosmotic stress signaling, but is located upstream of Rim15 and $PP2A^{Cdc55}$, and therefore does

not fulfill the criteria to be considered a potential counter kinase. The other CDKs have been primarily connected to transcription-related phosphorylation events (Nishizawa et al., 2010).
We could therefore assume that most of the PP2A^{Cdc55}-dependent, stress-induced S/T-P phosphorylation did not directly occur due to the activation of any MAPK or CDK. The fact that we found a significant threonine-enrichment in the these phosphorylation sites further substantiates the model that PP2A^{Cdc55} directly de-phosphorylates these sites (Agostinis et al., 1990; Godfrey et al., 2017), **Figure 3C**), and thus that inhibition of PP2A^{Cdc55} could be sufficient to induce the observed phosphorylation increase.

However, we agree with the Referee that to ultimately prove this hypothesis direct evidence would be required. This would entail the identification of kinases that contribute to the phosphorylation of this specific set of stress-induced S/T-P motifs and subsequent measurements of their activity prior to and upon hyperosmotic stress exposure. In fact, PP2A^{Cdc55} might target substrate subsets of multiple kinases that are already active under non-stress conditions, which would make the identification of involved kinases challenging.

We have updated the discussion (see below) to better convey these arguments and clarify that the most likely mechanistic scenario does not include activation of counter kinases. At the same time, further extended investigations would be needed to resolve the exact regulatory effects within the activated signaling network.

Changes in the Discussion:

"[...] The other CDKs in yeast have been primarily connected to transcription-related phosphorylation events (Nishizawa et al., 2010), and would therefore not account for the majority of targeted substrates. ~~There is also evidence that proline-directed MAP kinases cannot be involved since they are either not active or expressed at the onset of hyperosmotic stress, or target a different set of the response (García-Rodríguez et al., 2005; Hao et al., 2008; Pierce et al., 1998; Romanov et al., 2017).~~ There is also evidence that none of the five proline-directed MAP kinases in *S. cerevisiae* could act as the hypothetical stress-activated counter-kinase downstream of PP2A^{Cdc55}. The MAPK Smk1 is not expressed in haploid cells, and the MAPKs Slt2 and Fus3 do not become activated during the immediate stress response (Pierce et al., 1998, Hao et al., 2008, García-Rodríguez et al., 2005). We found over 90% of stress- and PP2A^{Cdc55}-dependent S/T-P motifs to be independent of Hog1, while deletion of the MAPK Kss1 has been described to not affect Hog1-independent, stress-induced S/T-P sites (Romanov et al., 2017). Thus, none of the proline directed kinases appear to act downstream of PP2A^{Cdc55}. Moreover, our experiments imply that the phosphatase directly targets these S/T-P phosphorylation sites, pointing towards a phosphatase-centric signaling mechanism as depicted in Figure 6. Based on our results we propose the following model: Under unstressed conditions the phosphatase and counteracting kinase activities are balanced, resulting in a steady state of low phosphorylation of shared substrates. Upon hyperosmotic stress the phosphatase becomes inhibited, tipping the balance towards increased net phosphorylation, rendering it a key event in regulating a global signaling response. In fact, our detailed analysis of

the phosphorylation behavior of the Cdc55 substrates Gis1 and Rph1 further substantiates the proposed model. In an alternative scenario, PP2A^{Cdc55} could inhibit the activity of one or several unknown kinases under non-stress conditions that share common S/T-P substrate sites with the phosphatase. Inhibition of PP2A^{Cdc55} would result in activation of these kinases, and therefore an increase in phosphorylation due to the combinatory effects of phosphatase inhibition and simultaneous kinase activation. However, as described in detail before, there is no evidence that the shift in the balance of substrate phosphorylation is associated with the activation of any known proline-directed kinase. Further studies will undoubtedly be required though to conclusively prove that no concurrent kinase activation takes place upon PP2A^{Cdc55} inhibition. There is no evidence that this shift in balance is associated with the activation of any known proline-directed kinase. In fact, our detailed analysis of the phosphorylation behavior of the Cdc55 substrates Gis1 and Rph1 further substantiates the proposed model. [...]"

2) Is there anything in their data that would show how the Rim15-Igo1/2 pathway gets activated upon stress? Is this through an increase in kinase activity activating Rim15?

Our findings do not indicate what particular stress-sensing mechanism leads to the inhibition of PP2A^{Cdc55} by Igo1 and Igo2 in the first place. We previously identified phosphorylations at Igo1 and Igo2 key residues in response to hyperosmotic stress with transient kinetics (Janschitz et al., 2019), suggesting the activation of the Greatwall protein kinase Rim15 under these conditions. In fact, we report stress-induced phosphorylation of four serines of the kinase (**Figure EV2A**), indicating that the phosphorylation pattern of Rim15 is affected by hyperosmolarity conditions. The function of the phosphorylated sites, however, is unknown and the only well characterized regulatory site of Rim15 has not been covered in our datasets (regulatory site T1075 targeted by 14-3-3, (Wanke et al., 2005)). The Rim15-Igo1/Igo2 module represents a highly conserved regulatory hub that integrates signals stemming from nutrient sensing, such as TORC1 and Ras/protein kinase A (PKA) signaling (Pedruzzi et al., 2003), and the so-called CDK complex Pho85/Pho80 (Jin et al., 2017; Wanke et al., 2005). Not only does a deletion of *PHO85* or *RIM15* cause severe defects in the transcriptional response to hyperosmotic stress (Chasman et al., 2014), the Pho85/Pho80 complex has also been described to mediate stress signaling, in parallel with the HOG pathway (Jin et al., 2017). It is therefore possible to speculate that, similarly to the MAPK pathway, the stress signal would propagate from the upstream kinase Pho85/Pho80 via Rim15 and Igo1 and Igo2 to its eventual effector protein PP2A^{Cdc55}. To sustain this argument, further experimental investigations would be needed, which we believe is beyond the scope of this manuscript.

Along these lines, a recent study monitored the immediate phosphorylation changes occurring within the first 60 seconds during hyperosmotic stress exposure (Kanshin et al., 2015). Within this dataset, we found that the key regulatory residues of Igo1 and Igo2 already display increasing phosphorylation within seconds after the stress treatment. This observation indicates that the upstream stress sensing and signaling components of the pathway must become

activated very rapidly. We have included this observation into the Results section and show it in **Figure EV2C.**

Changes in the Results:

"[...] The yeast Endosulfines Igo1 and Igo2 have been previously described as inhibitors of $PP2A^{Cdc55}$ that become activated upon phosphorylation of a specific residue by the Greatwall kinase Rim15 (Juanes et al., 2013a). Throughout the Rim15-Igo1/Igo2-Cdc55 regulatory module we observed stress-induced phosphorylation events (Figures EV2A and EV2B) and substantial phosphorylation at the respective key regulatory residues of Igo1 and Igo2 (Igo1-Ser^{64} 7.3x increase; Igo2-Ser^{63} 6x increase, Figure 2C). Phosphorylation of these residues can already be observed within seconds after hyperosmotic stress exposure, indicating a very rapid upstream sensing mechanism (Figure EV2C, Kanshin et al., 2015). Moreover, stress-induced phosphorylation of the Endosulfines appears to be transient and to return to almost basal levels after 15 minutes (Figure EV2D, Janschitz et al., 2019). Together these observations strongly point towards a typical stress-signaling response that mediates $PP2A^{Cdc55}$ inhibition in a very rapid and temporally regulated manner. ~~Both Igo1 and Igo2 showed strongly increased phosphorylation at their respective key regulatory residue upon hyperosmotic stress (Igo1-Ser^{64} 7.3x increase; Igo2-Ser^{63} 6x increase, Figure 2C). Moreover, transient phosphorylation of the yeast Endosulfines upon hyperosmotic stress treatment has recently been observed, indicating that $PP2A^{Cdc55}$ is inhibited upon stress exposure (Janschitz et al., 2019) (Figure EV2D).~~ [...]"

3) We recently showed that human PP2A-B55 (corresponding to PP2A-Cdc55) is able to act on proline directed sites while PP2A-B56 (corresponding to PP2A-Rts1) is very inefficient. This fits well with their observations described here so I would suggest to cite this since they already site work on PP2A-B55 from the human system.

We agree with the referee that their description of PP2A-B55 acting on proline directed sites coincides very well with our findings. We have added a reference to Kruse *et al.* (Kruse et al., 2020) after the following phrase in the results section: "$PP2A^{Cdc55}$ preferentially dephosphorylates phospho-threonines in threonine-proline (TP) motifs.".

POINT-BY-POINT REPLY: Referee #2

Review for "A phosphatase-centric mechanism drives stress signaling response"

Summary
In this manuscript, Hollenstein et al. investigate the role of protein phosphatase 2A bound to regulatory subunit Cdc55 (PP2ACdc55) as a main downstream effector in the Saccharomyces cerevisiae hyperosmotic stress response signaling pathway. Combining quantitative mass spectrometry (MS) experiments with molecular and biochemical approaches, the authors identify a role for PP2ACdc55 in regulating the hyperosmotic stress response via dephosphorylation of downstream substrates. Using several phosphoproteomic approaches, they demonstrate that nearly 1/3 of the stress-induced phosphorylome is under the control of PP2ACdc55. They also show that PP2ACdc55 can directly target stress-related proteins, many of which are associated with gene expression regulation. By focusing on the transcription factors Gis1 and Rph1, the authors demonstrate that PP2ACdc55 directly regulates the activity of these proteins to control the transcriptional response under hyperosmotic stress. These results guide them to a proposed model in which phosphatase inhibition, rather than increased kinase activity, induces a positive phosphorylation response to osmotic stress.

Overall, the experiments in this manuscript are elegantly executed, and the authors provide potentially interesting new insights into the roles of PP2A in regulating cellular stress responses. A few important concerns are noted. The authors need to rule out any potential indirect effects of cell cycle arrest in the PP2ACdc55-regulated phosphorylome (see major point #1 below). Also, the manuscript lacks clear experimental evidence that more definitively link their findings of the PP2ACdc55-regulated signaling events to the high osmolarity sensitivity phenotype observed by lack of Cdc55 (major point #2 below). Some experiments are also included with questionable comparability to other provided evidence due to different genotypes and should be addressed.

> We want to thank the referee for pointing out the quality of our work and the potential new insights on PP2A that we derive from it. We will address the major points raised in the following section.

Major Comments
1. Potential indirect effects of cell cycle arrest in the PP2ACdc55-regulated phosphorylome. PP2A plays key roles in mitotic progression, both during entry and exit of mitosis. It was previously reported that the cdc55Δ cells have a pronounced delay in G2/M as determined by flow cytometry (Yellman and Burke, MBOC, 2006: https://doi.org/10.1091/mbc.e05-04-0336). It was also previously shown that cells lacking PP2ACdc55 show gross morphological defects and poor growth due to Cdk inhibitory tyrosine kinase Swe1 activation (Godfrey et al., 2017, Wang and Burke, 1997). Moreover, cells lacking Cdc55 also show strong defect in mitotic exit as shown for example in Touati et al., 2019 (https://doi.org/10.1016/j.celrep.2019.10.041). Therefore, there is a major concern with the possibility that cell cycle effects are leading to confounding data interpretation. The authors find a range of S/T-P sites going up in cdc55delta. But since Cdc28 (CDK) is known to phosphorylate S/T-P sites, and mediate a wave of CDK phosphorylation in G2/M-M phases, one possibility is that the cdc55 mutant is partially

arrested at G2/M or M-phases, and that most sites going up in the mutant are actually Cdc28 substrates regulated during G2/M and/or M phases.

We appreciate the referee's careful consideration of our findings in the context of known $PP2A^{Cdc55}$ cell cycle functions and how these could be reflected in our data. We have taken the concern of a potential confounder in our data interpretation very seriously and have performed several additional analyses to exclude this possibility. On the experimental side, a measurement of the FACS profiles of *cdc55*Δ and Cdc55 depletion mutants confirmed that no significant cell cycle effects occur. Furthermore, we compared our phospho-proteomic data with a published dataset analyzing the immediate impact of Cdc28 inhibition on the phospho-proteome (Kanshin et al., 2017), and concluded that inhibition of $PP2A^{Cdc55}$ during hyperosmotic stress affects a significantly different set of phosphorylation sites than the CDK. This cumulative evidence has been incorporated in the manuscript (**Figure EV3**) and is described in more detail in the response to the editor above.

1a. The authors need to monitor cell cycle distribution in wt and in cdc55delta to make sure the changes are not due to changes in cell cycle. Even in the case of a fast 30 min depletion of Cdc55, that could be enough time to trap several cells in G2/M or M phases an up-regulate S/T-P sites. It is essential that the authors rigorously test this possibility by monitoring cell cycle distribution in the cells and conditions used for generating their phosphoproteomic analysis.

We agree with the referee and the suggested experimental setup. We have analyzed FACS profiles of wt, *cdc55*Δ and Cdc55 depletion mutants, and can exclude any potential impact on the phosphorylome due to cell cycle changes. Furthermore, we did not observe changes in the FACS profile induced by hyperosmotic stress treatment for ten minutes. The FACS profiles were included into the manuscript (**Figure EV1E),** for details please refer to the response to the editor above.

1b. The issue of potential cell cycle effects is a serious concern as it could imply that the authors are revealing the mitotic action of Cdc55, rather than its role in the osmotic stress response. Along those lines, it is concerning that the authors did not cite Touati et al, 2019. This work already performed phosphoproteomic analysis of cells lacking Cdc55 in the context of mitosis. How similar are the set of targets identified by that work compared to the present paper?

Prior to submitting this work, we already had reason to believe that no cell cycle effects occur given that several cell cycle-related marker proteins did not show an obvious trend towards increased or decreased abundance. We do, however, understand the referee's concern in the lack of documentation on this matter in the previous manuscript. This information has now been included in Figure EV1F.

According to the Referee's suggestion, we have compared Cdc55 target sets defined in our study with cell-cycle dependent phosphorylation sites as defined

by Touati *et al*., and also Godfrey *et al*. (Godfrey et al., 2017; Touati et al., 2019). Our analysis did not support any confounding effect of the cell cycle on our study**,** for details please refer to the response to the editor above.

Also, does the condition of osmotic stress used by the authors result in any cell cycle arrest on its own? This could also result in confounding data interpretation and needs to be carefully checked

It has indeed been reported that cells arrested in G1 fail to become released from the arrest upon hyperosmotic stress. This retardation in cell cycle progression is mediated via Hog1 phosphorylation of the CDK inhibitor Sic1 (Escoté et al., 2004), whereas in the absence of Hog1 G1-arrested cells are normally released even under hyperosmotic conditions. It has also been reported that asynchronous wild type cells display hardly any visible arrest in G1 after 20 minutes of exposure to hyperosmotic stress (Maayan and Engelberg, 2009). Moreover, it appears that even after 5 hours of stress treatment the cell population is still only partly arrested in G1. For the time points and conditions (5 or 10 minutes stress treatment) we chose, we could effectively demonstrate using FACS profiling that no significant cell cycle-related arrests at a specific phase occur. We included the evidence to document these findings in the manuscript as well. Please refer to the first part of this letter for a more detailed response.

Importantly, we found that the Hog1 and PP2A stress signaling responses are independent of each other, and that inhibition of Hog1 did not abrogate the stress-induced phosphorylation response caused by Cdc55 inhibition, although Hog1 is required for the cell cycle arrest during hyperosmotic stress. Thus, a potential arrest of wildtype cells immediately upon hyperosmotic stress exposure would not confound our data interpretation.

2. The manuscript lacks clear experimental evidence that more definitively link their findings of PP2ACdc55-regulated signaling events to the high osmolarity sensitivity phenotype observed by lack of Cdc55: 2a. It is surprising that there is no rescue effect in the igo1Δigo2Δcdc55Δ triple mutant compared with igo1Δigo2Δ, especially considering that Cdc55 depletion can rescue the mRNA level of two ESR genes to WT levels in Figure 4D & E. As this paper suggests that PP2ACdc55 inactivation upon stress is important for proper phosphorylation, it is somewhat confusing that cdc55Δ cells show such a strong stress sensitivity to osmotic treatment, but they grow normally under untreated condition. Does Cdc55-AID also show the same sensitivity to osmotic stress? Can a transient depletion of Cdc55 provide at least partial rescue of osmotic sensitivity in igo1Δigo2Δ cells?

In addition to the serial dilution spot assays (former **Figure 2D**, now **EV2E**) we have analyzed the effects on growth rates in response to increased osmolarity (former **Figure S2D,** now **2D**). This analysis revealed a subtle growth defect of *cdc55*Δ cells in YPD. Arguably, such a small effect might simply not be visible in the spot assay or one would have to assume that deletion of *CDC55* affects growth differently depending on whether the medium is solid or liquid. The

igo1Δigo2Δ double deletion showed an even more pronounced growth defect. Interestingly, this growth defect was rescued by additional deletion of *CDC55* (triple k.o.), under non-stress as well as stress conditions. The growth rate of the triple k.o. was almost equivalent to the *CDC55* single deletion, suggesting a full rescue of the growth rate in stress in the monitored time period.

In the serial dilution spot assay *cdc55Δ* displayed higher stress sensitivity than *igo1Δigo2Δ*, as fewer colonies were growing upon increased osmolarity. Considering the pathway architecture, deletion of Igo1/2 should not further affect the stress phenotype of *cdc55Δ*. However, since the *cdc55Δ* phenotype is already more severe than the *igo1Δigo2Δ*, the triple deletion should display the more severe phenotype of *cdc55Δ*. In addition, we observed a growth phenotype on YPD for *igo1Δigo2Δ* and the triple deletion but not for *cdc55Δ* alone - again indicated by smaller and fewer colonies. Hence the stress phenotype of the triple deletion is a combination of the slower growth of *igo1Δigo2Δ*, which in itself is unrelated to stress, and the more severe stress phenotype of *cdc55Δ*. Overall the results of the drop assay also fit well to the pathway architecture.

A drop assay experiment requires longer survival and growth of the cells in high osmotic conditions (former **Figure 2D**, now **EV2E**) compared to the shorter exposure time for growth rate calculations in liquid culture (former **Figure S2D,** now **2D**). In addition, it is likely that stress signaling via the Greatwall Kinase-ENSA-PP2A pathway needs to be dynamic for cells to adequately respond to the stress condition. Thus, either the absence of signaling induction as well as signaling termination (i.e. permanent signaling) would result in a problem, as we have also described in our Results section in the original manuscript. Hence it is not surprising to observe stress sensitivity for both *cdc55Δ* and *igo1Δigo2Δ*.

The efficiency and consistency of Cdc55 depletion cannot be monitored in a serial dilution spot assay; hence, we did not perform drop assays with transient depletion of Cdc55 as suggested by the referee. However, we agree with the referee that the drop assay and especially the observed combination of effects is not very intuitive and difficult to follow. Hence, we have exchanged the drop assay experiment (former **Figure 2D**, now **EV2E**) with the growth rate experiment (former **Figure S2D,** now **2D**), so that latter is now displayed in the main figure. We have adjusted the results accordingly and have also extended the description of both experiments in the results.

"[...] To test if the Endosulfines are required for coping with hyperosmotic stress we monitored the stress sensitivity of wild type and *igo1Δigo2Δ* cells in response to high osmolarity conditions using growth curves (Figure 2D). Cells lacking *IGO1/2* showed a moderate but clear susceptibility to increasing concentrations of NaCl, confirming that Igo1 and Igo2 are important for cellular survival under hyperosmotic stress conditions. Deleting *CDC55* also elicited a visible growth defect at concentrations above 0.3M NaCl, but less pronounced than with the *IGO1/2* double deletion. The growth defect of *igo1Δigo2Δ* cells could in turn be rescued by an additional deletion of *CDC55* (a triple knockout of *igo1Δigo2Δcdc55Δ*) under non-stressed as well as stress conditions. In fact, the growth rate of the triple knockout was almost equivalent to the growth rate of the

CDC55 single deletion, suggesting a full rescue in the monitored time period. In a similar experimental setup, we also looked at stress sensitivity to 0.8 M NaCl using a serial dilution spot assay (Figure EV2E). For both, *cdc55Δ* and *igo1Δigo2Δ*, we could confirm stress-induced growth defects. [...]"

2b. Do Gis1-5A and Rph1-5A cells show sensitivity to osmotic stress? How about gis1Δrph1Δ cells? It may be necessary to show a sensitivity assay when modulating these downstream targets. One may expect Gis1-5D and Rph1-5D to show resistance to osmotic stress. The authors should test this.

Following the suggestion of the referee we have analyzed the sensitivity of *gis1Δrph1Δ* and the mutant versions of the individual proteins (Gis1-5A, Rph1-5A, Gis1-5D, Rph1-5D) to osmotic stress. We did not observe any visible growth defects for either of these strains upon hyperosmotic stress (see **Figure below**).

We argue, however, that this result is not entirely unexpected, as the effects of the Greatwall-Igo1/2-PP2A pathway transcend beyond transcription towards various functional processes. Moreover, *the igo1Δigo2Δ* knockout-strain, which had a visible growth defect, shows a more severe transcription effect than *gis1Δrph1Δ*, indicating that even within the transcriptional landscape the PP2A pathway seems to regulate multiple effectors besides Gis1 and Rph1. The double knockout *gis1Δrph1Δ* does show, however, a reproducible and noticeably transient effect on mRNA levels after being exposed to hyperosmotic stress for 30 minutes (see **Figure 5F**). The decrease in mRNA levels is also apparent at 30 minutes for the 5A mutated versions of the transcription factors, with effect sizes ranging from 20-30%. Such transient effects on mRNA levels might only slightly affect the total protein levels of the transcribed genes. It is therefore plausible to assume that the effects we observe at the mRNA level simply do not elicit any visible growth phenotype as protein levels might be sufficiently adjusted for stress adaptation.

It has to be pointed out that Gis1 and Rph1 were selected for further analysis based primarily on their Igo1/2-dependent phosphorylation patterns in Cdc55-depleted conditions and hyperosmotic stress (see **Figure 5A**). We also validated that they represent direct substrates of the phosphatase. The transcriptional regulators thereby suited the purpose of studying the effects of phosphorylations *specifically* mediated by the Greatwall-Igo1/2-PP2A pathway in a time-resolved manner. These analyses were not only focused on a functional phenotypic readout, but also on the validation of the proposed model of how inhibition of PP2A regulates stress-induced phosphorylation.

Figure for the Referee’s attention:

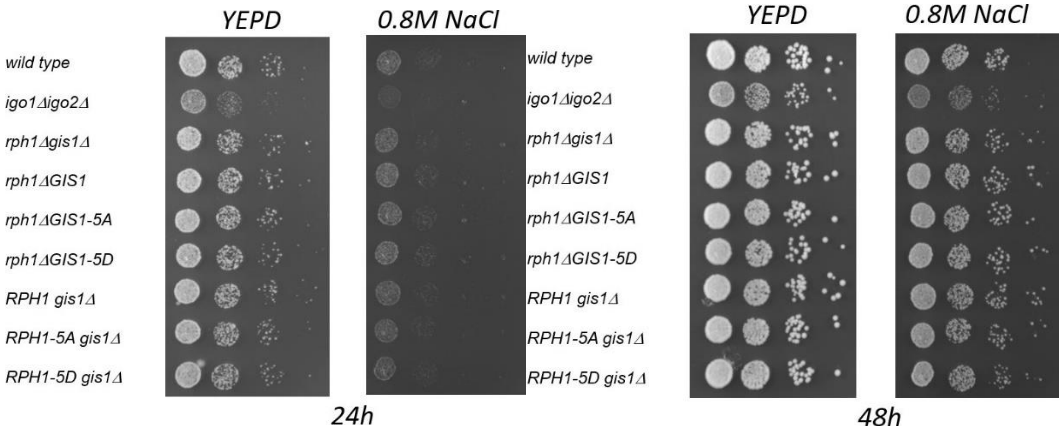

Other Comments:

1. The M-track experiment to test for in vivo interaction of PP2ACdc55 with Gis1 and Rph1 involves using a rim15Δ to prevent inhibition by Igo1/2, whereas the phosphoproteome experiment immediately preceding this simply used an igo1/2Δ. It is currently unclear why two different approaches were used to prevent inhibition by Igo1/2, and it raises concerns as to the compatibility of the phosphoproteomic data and the M-track results.

We want to highlight that interaction between PP2A^{Cdc55} and Gis1 has also previously been successfully demonstrated by cross-linking Co-IP (Bontron et al., 2013). We confirmed this direct interaction using an alternative assay that indicates very close protein proximity, and also found a similar interaction signal between PP2A^{Cdc55} and Rph1.

The purpose of the phospho-proteome experiments was to decipher phosphorylation sites directly affected by PP2A^{Cdc55} during stress. Using a *rim15*Δ deletion mutant for this experimental setting would not have been ideal as the deletion could potentially cause additional effects on the phosphoproteome compared to igo1/igo2, given that Rim15 is itself a kinase acting upstream of Igo1/Igo2. For the M-track experiment, on the other hand, we investigated whether PP2A^{Cdc55} interacts with its putative substrates Gis1 and Rph1. PP2A is expected to interact with these proteins under non-stressed conditions, when Rim15 is inactive. Since phosphatase-substrate interactions are notoriously difficult to detect, we decided to use the most promising experimental conditions by preventing any potential Cdc55 inhibition by phosphorylated Igo1/Igo2.

Essentially, however, there is no reason to believe why in this case a deletion of *RIM15* would not render the same effect as a double deletion of *IGO1/2,* which is blocking PP2A^{Cdc55} inhibition.

2. Can the authors speculate on why stress- and cdc55Δ-induced phosphorylation sites showed a trend towards higher phosphorylation in setup SR cdc55Δ (Figure 2E) since kinase activity is not changed upon stress suggested by the model?

We want to point out to the referee that in the setups "SR *cdc55∆*" and "SR *igo1∆igo2∆*" we quantified fold changes between wild type and knockout cells that were both exposed to hyperosmotic stress, and not between unstressed and stressed knock-out cells, as we suspect might have been assumed by the referee. In this case it is straightforward to explain why stress- and *cdc55∆*-induced phosphorylation sites showed a trend towards higher phosphorylation in setup SR *cdc55∆*. In stressed wild type cells $PP2A^{Cdc55}$ might not be completely inhibited, whereas in the knock-out no phosphatase activity remains. In addition, the knock-out displays constitutive low phosphatase activity, even before stress, whereas in the wild type the phosphatase was only inhibited for a short duration (at maximum ten minutes of stress exposure).

3. All targets in Sup. Fig 3 have 3 loadings. Do all of them follow a series of dilution or just 3 replicates with same loading? It's better to clarify this in figure legends.

As suggested by the referee, we have adjusted the figure legend of **Supplementary Figure 3** (now referring to **Appendix Figure S2**) accordingly. We have added the information that the loadings correspond to 3 replicates.
"[...] **Appendix Figure S2 (related to Figure 3D).** Scanned western blot films showing M-track protein-protein proximity results of Cdc55 and the individual candidates (in 3 replicates, respectively). (A) Experimental series 1. [...]"

4. The color scheme of Figure 4E makes it hard to discern exactly where the bars end. Solid coloration rather than gradient might serve this plot better.

As suggested by the referee, we have adjusted **Figure 4E** and in addition **Figure 5F** accordingly.

5. In Figure 5A, is there any reason not to choose setup SR and setup cdc55Δ?

We compare the "induction" of the response in wild type cells after stress exposure with the "inhibition" of the response by comparing stressed wild type with stressed *igo1∆/igo2∆* cells. In the first case Cdc55 becomes inhibited, whereas in the second it does not. Thereby we can specifically identify the part of the stress response that is strongly dependent on the inhibition of $PP2A^{Cdc55}$.

6. It may be advisable to also show the quantification and Q-value for Gis1 and Rph1, like in Figure 3D.

Given that we only have two replicates for the negative control in Figure 3D, it is not advisable to calculate *q*-values in this case. We only used *q*-values in the scenario where we tested many different proteins for interaction and needed to have a statistical measure to control for multiple testing, especially given that some signals were close to the background noise. In the case of Figure 3D, however, the effects are clearly visible and hence interpretable.

7. There should be a loading control in Figure 5D, as phosphorylation of Rph1-HA is not obvious. Instead, it looks like there is more protein at later time points.

The loading control (in this case, Cdc28), can be found in **Figure EV5C** (former **Supplemental Figure 5C**). We adjusted the legends of figure 5D and S5C to better indicate this. We want to thank the referee for pointing this out.

"[...] **Figure 5**. [...] (D) Mobility shift assay of Gis1 and Rph1 across several time points after Cdc55 depletion (without exposure to hyperosmotic stress). Cdc28: loading control (shown in Figure EV5C). [...]"

8. The method of phosphorylation site deconvolution used to determine which sites on Gis1 and Rph1 are affected by PP2ACdc55 is not terribly intuitive as described. The authors should clarify how the deconvoluted sites can be definitively attributed to PP2ACdc55 amongst all the regulatory changes occurring during the hyperosmotic stress response.

We agree with the referee that the selection of site mutations might not appear intuitive, but we now added a more detailed description in the Methods section. In essence, we took into account four major considerations, namely (i) keeping the overall number of mutations at a minimum, (ii) mutating enough sites to elicit a potential phenotype, (iii) ensuring that the sites had a stoichiometric phosphorylation and (iv) choosing sites that are conserved between the two paralog histone demethylases. We might not cover all $PP2A^{Cdc55}$-targeted sites, but we do make sure that we mutate as many potential sites as possible, while not running the risk of capturing too many redundant sites.

In more detail, we added this new section to the methods as an explanation for our selection of sites to be mutated in.

"[...] **Selection of Sites for Mutational Analysis.** Phosphorylation sites for mutational analysis were selected according to whether they become increasingly induced by Cdc55-depletion in a time-dependent manner and are also found to be induced in setup SR. Only two phosphorylation sites on each protein fulfilled these strict criteria, Ser^{425} and Ser^{690} of Gis1 and Ser^{412} and Ser^{430} of Rph1. Given that both proteins are paralogues, regulated phosphorylation sites that are conserved between these proteins could also be potentially interesting (Strumillio et al, 2019), i.e. Ser^{425} of Gis1 being homologous to Ser^{430} of Rph1. Ser^{405} of Gis1 (homologue to Ser^{412} of Rph1) and Ser^{584} of Rph1 (homologue to Gis1 Ser^{690}) were therefore also included in the mutational analysis. The MS data suggests Cdc55-dependency for both of these phosphorylation sites. Moreover, Ser^{405} of Gis1 and Ser^{412} of Rph1 lie in a conserved cluster of three juxtaposed phospho-accepting residues with occasional ambiguous phosphorylation site allocation in some peptide hits. An additional serine-to-alanine mutation was therefore introduced in this cluster, namely at Ser^{403} of Gis1 and at Ser^{410} of Rph1. A second conserved cluster affected by both stress and Cdc55-depletion was observed, however, with decreasing phosphorylation levels (at Ser^{696} of Gis1 and Ser^{590} of Rph1) in response to the experimental conditions. [...]"

POINT-BY-POINT REPLY: Referee #3:

To assess the roles that PP2A complexes (i.e. those built with Cdc55 vs. Rts1 B regulatory subunits) play in coordination of the response to hyperosmotic shock, Hollenstein et al. performed a series of SILAC-based quantitative phosphoproteomics MS experiments. Specifically, they assess the effects of different mutant backgrounds on the osmoshock-induced changes in the phosphoproteome. Data for these analyses comes not only from this present work but also previously published works including an online BioRiv preprint from the group.

The group found a high degree of overlap between sites affected by osmotic shock and CDC55 or RTS1 deletion and conclude both that PP2A serves to oppose or contribute to the osmotic shock response, and that Cdc55 and Rts1 complexes have largely distinct effectors/substrates. Interestingly, the authors conclude that Rts1 counteracts a portion of Hog1-dependent signaling while Cdc55 affects largely Hog1-independent osmoshock signaling.

The authors then focus on the endosulfines Igo1/2 which are transiently hyperphosphorylated upon osmoshock suggesting a mechanism of osmo-shock induced inhibition of PP2A. Indeed, they discovered that the majority of Cdc55- and stress-dependent phosphosites are misregulated in igo1/2Δ cells whereas loss of igos has no impact on Hog1-dependent phosphosites. From these observations the authors conclude that inhibition of PP2A-Cdc55 is mechanistically responsible for approximately 1/3 of the phosphoproteomic response to osmotic shock which is comparable in magnitude to the regulation independently played by the classical Hog1 MAPK pathway.

Subsequent analyses demonstrate that PP2A-Cdc55 affects about 1/2 of all osmoshock regulated S/T-P motifs, whereas only 12% are regulated by Hog1-dependent signals. An M-Track protein-protein proximity assay was then used to identify/confirm putative direct PP2A-Cdc55 substrates. Next, the authors engineered a CDC55-depletion system to assess the role of this PP2A-Cdc55 in the transcriptional response to osmoshock. This line of investigation demonstrated that Igo-dependent inhibition of PP2A-Cdc55 is required for full induction of ESR genes. The relevant signaling was then shown to be mediated through Gis1 and Rph1 transcription factors, which, according to M-Track experiments, are likely direct substrates of PP2A-Cdc55.

Globally, this manuscript presents a robust dataset that reveals a complete and interesting narrative. Although the concept that PP2A-Cdc55 represents a key regulatory node in the cellular response to osmotic shock signaling is appreciated, the novelty of a phosphatase (as opposed to a kinase) being regulated as a mediator of signal transduction is rather oversold.

> We agree with the referee’s accurate summary of our work. We also understand the concern that we discussed the model too enthusiastically in some sections. We have changed several text sections and added a new paragraph to the Discussion as indicated in the updated manuscript (see details below), but do persist on the notion that the exact mechanism of the described phosphatase-centric pathway is novel.

Major comments 1.

The authors should dial back their enthusiasm of discovering that PPase regulation is a novel modus operandi in signal transduction. Indeed, analogous PPase regulation is well

documented for PP1 (downstream of glucose) as well as PP2B/calcineurin (downstream of Ca2+). The authors should acknowledge this prior literature and put their results into proper context.

We fully agree with the Referee that regulation of phosphatase activity as part of signal transduction cascade has been described previously and is not a novel discovery *per se,* and that we need to put our results into proper context of prior literature.

In our study we did not want to propose that phosphatase regulation is a novel signaling mechanism, but rather that the inhibition of basal activity of $PP2A^{Cdc55}$ (upon stress) is sufficient to cause a broad *induction* of phosphorylation sites, which to our knowledge has not been described in such detail before. It was not our intention to omit any information or literature, and we value the suggestion to include additional literature on PP1 and PP2B signaling in our work. We believe that there are some relevant and interesting differences between the phosphatase-centric model that we suggest and the mechanisms underlying PP2B and PP1 signaling.

PP2B has been mainly described to become activated by increased intracellular Ca2+ levels, which results in *dephosphorylation* of PP2B substrates (Creamer, 2020; Park et al., 2019). Hence, we believe that the mechanism behind PP2B signaling does not relate to the signaling model. Nevertheless, we have incorporated this point into our discussion.

PP1, on the other hand, displays several similarities to the GWL-ENSA-PP2A signaling response described in our study and we want to thank the Referee for highlighting that. More specifically, the inhibition of PP1 by PKA via DARPP32 has been described to *induce* phosphorylation in neurons (Leslie and Nairn, 2019). This primarily affects PKA substrates; it has not been systematically described to what extent the inhibition of basal PP1 activity transcends towards non-PKA substrates. In this scenario the phosphorylation response induced by PKA signaling appears to require *both* activation of PKA and inhibition of PP1, since the kinase and phosphatase act on shared substrates. This appears to be similar to the scenario described for CDK activation and PP2A inhibition during cell cycle progression. Whether the exclusive inhibition of PP1 in the absence of PKA activation would also be sufficient to induce a positive phosphorylation response on a broad set of substrates, and thus correspond to the exact same mechanism we describe for $PP2A^{Cdc55}$, has not been addressed to our knowledge. This would be particularly exciting as we speculated in our discussion on the presence of the suggested model in other systems.

To acknowledge the prior literature and to better put our findings into proper context we have made the following changes throughout the manuscript:

We have added the following section to the discussion:
"[...] Our description of such a phosphatase-driven signaling response leads to the question as to whether this mode of action applies to other phosphatases. There are two

other prominent phosphatase-centric pathways where the phosphatase represents a regulated effector element that directly targets a broad set of substrates, PP2B and PP1. PP2B has been mainly described to become activated by increased intracellular Ca2+ levels, which results in dephosphorylation of PP2B substrates (Hee-Soo Park et al. 2019, Creamer 2020). This is distinctly different from our proposed model, where the basal activity of the phosphatase becomes inhibited causing the exact opposite impact on the phosphorylome. PKA signaling, on the other hand, seems to follow a similar architecture as we describe here with the inhibition of PP1 leading to increased phosphorylation for example in neurons (Leslie and Nairn 2019). This seems to primarily affect PKA substrates, which appear to require both activation of PKA and inhibition of PP1. It is likely that inhibition of PP1 could be sufficient to induce phosphorylations of non-PKA substrates as well, and thus could represent a similar modus operandi as we describe for PP2A^{Cdc55} in the context of stress signaling. Such mechanistic details and their systematic dissection still remain to be explored for many phosphatases. One of the reasons that could complicate such studies might lie in the difficulty of tracking phosphatase activity and understanding the underlying complex regulatory mechanisms (Fahs et al., 2016; Shi, 2009). [...]"

In addition, we have made changes to the abstract,

"[...] Our results ~~reveal that phosphatases~~ demonstrate how a phosphatase can assume the role of active downstream effectors during signaling and allow re-evaluating the impact of phosphatases on shaping the phosphorylome. [...]"

… the introduction,

"[...] The contribution of protein phosphatases in defining the state of the phosphorylome, on the other hand, has largely not been investigated in depth. This is due to the fact that phosphatases have been less studied than kinases overall, and are ~~generally~~ typically not considered to be effector proteins of signaling networks (Brautigan, 2013; Smoly et al., 2017). [...]"

"[...] Our findings therefore point towards ~~a novel and~~ an unexpected signaling mechanism, based on the inhibition of a phosphatase instead of activation of a kinase as the central effector component of a stress-induced signal transduction pathway. [...]"

… and to the discussion.

"[...] Here we propose a ~~novel~~ signaling principle, in which the inhibition of a phosphatase is the key event resulting in increased phosphorylation, while induction of kinases affecting the same substrates appears not to be required. [...]"

"[...] The ~~existence of such a~~ discovery of this phosphatase-centric signaling mechanism is surprising since it represents the exact opposite to the traditional view of the rather passive role of phosphatases as antagonists of kinase-mediated signaling. [...]"

Minor comments

1. I do not understand the logic of this sentence: "We next focused on the role of PP2ACdc55 in the transcriptional regulation in response to hyperosmotic stress. Previously we observed a reduced transcriptional response to hyperosmotic stress in cdc55Δ cells (Reiter et al., 2013), which is in contrast to the finding that inhibition of

PP2ACdc55 is part of the stress response." If a component of the relevant signaling pathway is missing, would one not expect the response to be muted?

We want to note here that the effect of a missing component in a signaling pathway depends on its role in the pathway. In the context of this particular pathway model, we can expect Rim15 and Igo1/Igo2 to become activated upon hyperosmotic stress, and $PP2A^{Cdc55}$ to subsequently get inhibited. $PP2A^{Cdc55}$ activity becomes lower, and transcriptional activity increases. By deleting Cdc55 one therefore elicits the same effect as in the wild type - the phosphatase activity becomes low, and the transcriptional response is supposed to increase. Hence, the previous observation that deletion of Cdc55 results in the exact reverse, a decrease in transcription, does not fit the model, and is probably due to adaptation effects of the knockout strain. This was a major motivation to design a Cdc55 depletion strain to avoid any such effect and to observe the transcriptional response in an unbiased manner. We hope that this explanation resolves the referee's question.

2. When introducing Igo1/Igo2 as specific inhibitors of PP2A, I would explicitly mention that they act by binding to PP2A. This is of course logical but may not be obvious to every reader.

We agree with the referee that this fact might escape the reader's attention. We have therefore added a citation of the work by Williams *et al*. (Williams et al., 2014) and Thai *et al*. (Thai et al., 2017), where direct interaction between $PP2A^{Cdc55}$ and Igo1/2 was demonstrated by Co-IP. We have added the following information:

"[...] The inhibition of $PP2A^{Cdc55}$ works through a conserved module composed of the Greatwall Kinase Rim15 and the Endosulfines Igo1 and Igo2, which represent specific inhibitors of $PP2A^{Cdc55}$ (Gharbi-Ayachi et al., 2010; Mochida et al., 2010). Phosphorylation of Igo1 and Igo2 by Rim15 promotes their binding to $PP2A^{Cdc55}$, which results in inhibition of the phosphatase by a mechanism termed unfair competition (Williams et al., 2014, Thai et al., 2017). [...]"

3. Fig. 5E: Please indicate what samples were analyzed in the last two lanes of these blots. Also, normal SDS-PAGE gels could be useful here to assess if the 5A-mutant proteins are really more abundant or rather just transfer more efficiently out of the Phos-tag gels.

We thank the referee for pointing out this figure flaw. The last two lanes of the indicated blots in **Figure 5E** refer to the non-mutated protein version, and are used as a reference point. We agree though that this could cause confusion, and removed these lanes from the figure.

4. Fig. 5F, Suppl. Fig. 5D: The authors claim that they see decreased levels of CTT1 and PGM2 mRNA expression upon expressing the 5A mutants of Gis1 and Rph1. These results could be better described explaining that this difference is only apparent at later

time points and that induction at earlier time points appears to be independent of these transcription factors.

We agree with the referee that the outcome of the experiment was not thoroughly described and have therefore expanded the following section to include the referee's comment.

"[...] We next tested the effect of the alanine substitutions on the transcriptional response, using a *gis1Δrph1Δ* strain expressing the respective wildtype or point-mutated version of one of the proteins from plasmid. We recorded the transcriptional profiles of *CTT1* and *PGM2* during a period of 45 minutes exposure to hyperosmotic stress (Figure EV5D). While the initial increase of mRNA levels was not affected by the absence of both transcription factors, we observed a premature decline after 30 minutes post stress exposure. These results suggest that Gis1 and Rph1 do not function during the early induction of the transcriptional stress-response but rather support a high transcriptional output over an extended period of time. Since we observed the strongest effect after 30 minutes exposure to hyperosmotic stress, we re-measured this time point in a separate experiment and observed the mRNA levels of *CTT1* and *PGM2* decrease by 50% to 100% for the 5A mutant alleles relative to the respective knock out (Figure 5F). ~~the protein from plasmid. We observed decreased levels of *CTT1* and *PGM2* expression upon exposure to hyperosmotic stress when comparing wildtype with the mutated proteins (Figure 5F and Supplementary Figure 5D).~~We therefore propose that direct dephosphorylation by PP2A^{Cdc55} suppresses the transcriptional activity of Gis1 and Rph1 and that hyperosmotic stress-mediated inhibition of the phosphatase is necessary to allow phosphorylation-induced activation of these transcription factors. [...]"

REFERENCES

Agostinis, P., Goris, J., Pinna, L.A., Marchiori, F., Perich, J.W., Meyer, H.E., and Merlevede, W. (1990). Synthetic peptides as model substrates for the study of the specificity of the polycation-stimulated protein phosphatases. Eur. J. Biochem.

Bontron, S., Jaquenoud, M., Vaga, S., Talarek, N., Bodenmiller, B., Aebersold, R., and De Virgilio, C. (2013). Yeast Endosulfines Control Entry into Quiescence and Chronological Life Span by Inhibiting Protein Phosphatase 2A. Cell Rep.

Chasman, D., Ho, Y., Berry, D.B., Nemec, C.M., MacGilvray, M.E., Hose, J., Merrill, A.E., Lee, M.V., Will, J.L., Coon, J.J., et al. (2014). Pathway connectivity and signaling coordination in the yeast stress-activated signaling network. Mol. Syst. Biol.

Creamer, T.P. (2020). Calcineurin. Cell Commun. Signal.

Escoté, X., Zapater, M., Clotet, J., and Posas, F. (2004). Hog1 mediates cell-cycle arrest in G1 phase by the dual targeting of Sic1. Nat. Cell Biol.

García-Rodríguez, L.J., Valle, R., Durán, Á., and Roncero, C. (2005). Cell integrity signaling activation in response to hyperosmotic shock in yeast. FEBS Lett.

Godfrey, M., Touati, S.A., Kataria, M., Jones, A., Snijders, A.P., and Uhlmann, F. (2017). PP2ACdc55 Phosphatase Imposes Ordered Cell-Cycle Phosphorylation by Opposing Threonine Phosphorylation. Mol. Cell *65*, 393-402.e3.

Hao, N., Zeng, Y., Elston, T.C., and Dohlman, H.G. (2008). Control of MAPK specificity by feedback phosphorylation of shared adaptor protein Ste50. J. Biol. Chem.

Janschitz, M., Romanov, N., Varnavides, G., Hollenstein, D.M., Gérecová, G., Ammerer, G., Hartl, M., and Reiter, W. (2019). Novel interconnections of HOG signaling revealed by combined use of two proteomic software packages. Cell Commun. Signal. *17*.

Jin, N., Jin, Y., and Weisman, L.S. (2017). Early protection to stress mediated by CDK-dependent PI3,5P 2 signaling from the vacuole/lysosome. J. Cell Biol.

Kanshin, E., Bergeron-Sandoval, L.P., Isik, S.S., Thibault, P., and Michnick, S.W. (2015). A Cell-Signaling Network Temporally Resolves Specific versus Promiscuous Phosphorylation. Cell Rep. *10*, 1202–1214.

Kanshin, E., Giguère, S., Jing, C., Tyers, M., and Thibault, P. (2017). Machine learning of global phosphoproteomic profiles enables discrimination of direct versus indirect kinase substrates. Mol. Cell. Proteomics.

Kelliher, C.M., Foster, M.W., Motta, F.C., Deckard, A., Soderblom, E.J., Arthur Moseley, M., and Haase, S.B. (2018). Layers of regulation of cell-cycle gene expression in the budding yeast Saccharomyces cerevisiae. Mol. Biol. Cell.

Kruse, T., Gnosa, S.P., Nasa, I., Garvanska, D.H., Hein, J.B., Nguyen, H., Samsøe-Petersen, J., Lopez-Mendez, B., Hertz, E.P.T., Schwarz, J., et al. (2020). Mechanisms of site-specific dephosphorylation and kinase opposition imposed by PP2A regulatory subunits. EMBO J.

Leslie, S.N., and Nairn, A.C. (2019). cAMP regulation of protein phosphatases PP1 and PP2A in brain. Biochim. Biophys. Acta - Mol. Cell Res.

Maayan, I., and Engelberg, D. (2009). The yeast MAPK Hog1 is not essential for immediate survival under osmostress. FEBS Lett.

Minshull, J., Straight, A., Rudner, A.D., Dernburg, A.F., Belmont, A., and Murray, A.W. (1996). Protein phosphatase 2A regulates MPF activity and sister chromatid cohesion in budding yeast.

Curr. Biol.

Moyano-Rodriguez, Y., and Queralt, E. (2020). PP2A functions during mitosis and cytokinesis in yeasts. Int. J. Mol. Sci.

Nishizawa, M., Tanigawa, M., Hayashi, M., Maeda, T., Yazaki, Y., Saeki, Y., and Toh-e, A. (2010). Pho85 kinase, a cyclin-dependent kinase, regulates nuclear accumulation of the Rim101 transcription factor in the stress response of Saccharomyces cerevisiae. Eukaryot. Cell.

Park, H.S., Lee, S.C., Cardenas, M.E., and Heitman, J. (2019). Calcium-Calmodulin-Calcineurin Signaling: A Globally Conserved Virulence Cascade in Eukaryotic Microbial Pathogens. Cell Host Microbe.

Pedruzzi, I., Dubouloz, F., Cameroni, E., Wanke, V., Roosen, J., Winderickx, J., and De Virgilio, C. (2003). TOR and PKA Signaling Pathways Converge on the Protein Kinase Rim15 to Control Entry into G0. Mol. Cell.

Pierce, M., Wagner, M., Xie, J., Gailus-Durner, V., Six, J., Vershon, A.K., and Winter, E. (1998). Transcriptional Regulation of the SMK1Mitogen-Activated Protein Kinase Gene during Meiotic Development inSaccharomyces cerevisiae. Mol. Cell. Biol.

Romanov, N., Hollenstein, D.M., Janschitz, M., Ammerer, G., Anrather, D., and Reiter, W. (2017). Identifying protein kinase–specific effectors of the osmostress response in yeast. Sci. Signal. *10*, eaag2435.

Thai, V., Dephoure, N., Weiss, A., Ferguson, J., Leitao, R., Gygi, S.P., and Kellogg, D.R. (2017). Protein kinase C controls binding of Igo/ENSA proteins to protein phosphatase 2A in budding yeast. J. Biol. Chem. *292*, 4925–4941.

Touati, S.A., Hofbauer, L., Jones, A.W., Snijders, A.P., Kelly, G., and Uhlmann, F. (2019). Cdc14 and PP2A Phosphatases Cooperate to Shape Phosphoproteome Dynamics during Mitotic Exit. Cell Rep.

Wanke, V., Pedruzzi, I., Cameroni, E., Dubouloz, F., and De Virgilio, C. (2005). Regulation of G0 entry by the Pho80-Pho85 cyclin-CDK complex. EMBO J.

Williams, B.C., Filter, J.J., Blake-Hodek, K.A., Wadzinski, B.E., Fuda, N.J., Shalloway, D., and Goldberg, M.L. (2014). Greatwall-phosphorylated Endosulfine is both an inhibitor and a substrate of PP2A-B55 heterotrimers. Elife.

1st Revision - Editorial Decision 19th Aug 2021

Dear Dr. Reiter

Thank you for the submission of your revised manuscript to EMBO reports. We have now received the reports from the referees that were asked to evaluate it (copied below).

As you will see, both referees are very positive about the study and recommend publication. Before I can accept the manuscript, I need you to address some editorial points below:

- Please reduce the number of keywords to 5.

- We noticed that many references appear incomplete in the reference list, with issue and page numbers missing. Please carefully check the list and add the missing information.

- Please add callouts to the panels of Appendix Figure S3.

- Tables EV2, EV3, EV6 and EV7 are complex tables and should be changed to the file format "Dataset". The nomenclature needs to be changed to Dataset EVx for these.
- The remaining tables can remain as EV tables but will need renaming to Table EV1-EV3. Please also use Table EVx as name in the respective files itself instead of "Expanded View table X"
- Please remove the legends for EV tables from the main manuscript file and add them to the files themselves (if not present already). Each EV table or Dataset must contain the file name and a legend, either in the first column or in a separate tab. This is currently missing for Table EV7.

- The Appendix needs a title page with a table of content and page numbers.

- The Figure Legends should follow-on from the References.

- When you refer to datasets you have re-analysed (Romanov et al 2017, Hollenstein et al 2020, Kanshin et al 2017) you could also add a Data reference that directly refers to the dataset in addition to citing the manuscript. E.g. (Romanov et al, 2017, Data ref: Romanov et al, 2017). The Data ref refers to the dataset in a public database and lists the authors, the dataset identifier and the link that resolves to the dataset. Please check whether this is applicable. Further information can be found in our Guide to Authors under "Data citation" (https://www.embopress.org/page/journal/14693178/authorguide#referencesformat)

- Finally, EMBO reports papers are accompanied online by A) a short (1-2 sentences) summary of the findings and their significance, and B) 2-3 bullet points highlighting key results. Please send us this information along with the revised manuscript.

We look forward to seeing a final version of your manuscript as soon as possible.

Kind regards,

Martina Rembold

Martina Rembold, PhD
Senior Editor
EMBO reports

****

Referee #2:

The authors have addressed all my concerns.

Referee #3:

The authors have suitably addressed my previous comments.

The authors have addressed all minor editorial requests.

2nd Revision - Editorial Decision 3rd Sep 2021

Dr. Wolfgang Reiter
Max Perutz Labs, University of Vienna
Mass Spectrometry Facility
Vienna BioCenter
Dr. Bohrgasse 9
Vienna 1030
Austria

Dear Wolfgang,

Thank you for sorting out the issue with the data reference. I am now pleased to accept your manuscript for publication in the next available issue of EMBO reports. Thank you for your contribution to our journal.

At the end of this email I include important information about how to proceed. Please ensure that you take the time to read the information and complete and return the necessary forms to allow us to publish your manuscript as quickly as possible.

As part of the EMBO publication's Transparent Editorial Process, EMBO reports publishes online a Review Process File to accompany accepted manuscripts. As you are aware, this File will be published in conjunction with your paper and will include the referee reports, your point-by-point response and all pertinent correspondence relating to the manuscript.

If you do NOT want this File to be published, please inform the editorial office within 2 days, if you have not done so already, otherwise the File will be published by default [contact: emboreports@embo.org]. If you do opt out, the Review Process File link will point to the following statement: "No Review Process File is available with this article, as the authors have chosen not to make the review process public in this case."

Should you be planning a Press Release on your article, please get in contact with emboreports@wiley.com as early as possible, in order to coordinate publication and release dates.

Thank you again for your contribution to EMBO reports and congratulations on a successful publication. Please consider us again in the future for your most exciting work.

Kind regards,

Martina

Martina Rembold, PhD
Senior Editor
EMBO reports

****

THINGS TO DO NOW:

You will receive proofs by e-mail approximately 2-3 weeks after all relevant files have been sent to our Production Office; you should return your corrections within 2 days of receiving the proofs.

Please inform us if there is likely to be any difficulty in reaching you at the above address at that time. Failure to meet our deadlines may result in a delay of publication, or publication without your corrections.

All further communications concerning your paper should quote reference number EMBOR-2021-52476V3 and be addressed to emboreports@wiley.com.

Should you be planning a Press Release on your article, please get in contact with emboreports@wiley.com as early as possible, in order to coordinate publication and release dates.

EMBO PRESS

YOU MUST COMPLETE ALL CELLS WITH A PINK BACKGROUND ↓

PLEASE NOTE THAT THIS CHECKLIST WILL BE PUBLISHED ALONGSIDE YOUR PAPER

Corresponding Author Name: Wolfgang Reiter

Journal Submitted to: EMBO Reports

Manuscript Number: EMBOR-2021-52476V2

USEFUL LINKS FOR COMPLETING THIS FORM

http://www.antibodypedia.com
http://1degreebio.org
http://www.equator-network.org/reporting-guidelines/improving-bioscience-research-repor
http://grants.nih.gov/grants/olaw/olaw.htm
http://www.mrc.ac.uk/Ourresearch/Ethicsresearchguidance/Useofanimals/index.htm
http://ClinicalTrials.gov
http://www.consort-statement.org
http://www.consort-statement.org/checklists/view/32-consort/66-title
http://www.equator-network.org/reporting-guidelines/reporting-recommendations-for-tumo
http://datadryad.org
http://figshare.com
http://www.ncbi.nlm.nih.gov/gap
http://www.ebi.ac.uk/ega
http://biomodels.net/
http://biomodels.net/miriam/
http://jjj.biochem.sun.ac.za
https://osp.od.nih.gov/biosafety-biosecurity-and-emerging-biotechnology/
http://www.selectagents.gov/

Reporting Checklist For Life Sciences Articles (Rev. June 2017)

This checklist is used to ensure good reporting standards and to improve the reproducibility of published results. These guidelines are consistent with the Principles and Guidelines for Reporting Preclinical Research issued by the NIH in 2014. Please follow the journal's authorship guidelines in preparing your manuscript.

A- Figures
1. Data
The data shown in figures should satisfy the following conditions:

- the data were obtained and processed according to the field's best practice and are presented to reflect the results of the experiments in an accurate and unbiased manner.
- figure panels include only data points, measurements or observations that can be compared to each other in a scientifically meaningful way.
- graphs include clearly labeled error bars for independent experiments and sample sizes. Unless justified, error bars should not be shown for technical replicates.
- if n< 5, the individual data points from each experiment should be plotted and any statistical test employed should be justified
- Source Data should be included to report the data underlying graphs. Please follow the guidelines set out in the author ship guidelines on Data Presentation.

2. Captions

Each figure caption should contain the following information, for each panel where they are relevant:

- a specification of the experimental system investigated (eg cell line, species name).
- the assay(s) and method(s) used to carry out the reported observations and measurements
- an explicit mention of the biological and chemical entity(ies) that are being measured.
- an explicit mention of the biological and chemical entity(ies) that are altered/varied/perturbed in a controlled manner.

- the exact sample size (n) for each experimental group/condition, given as a number, not a range;
- a description of the sample collection allowing the reader to understand whether the samples represent technical or biological replicates (including how many animals, litters, cultures, etc.).
- a statement of how many times the experiment shown was independently replicated in the laboratory.
- definitions of statistical methods and measures:
  - common tests, such as t-test (please specify whether paired vs. unpaired), simple χ2 tests, Wilcoxon and Mann-Whitney tests, can be unambiguously identified by name only, but more complex techniques should be described in the methods section;
  - are tests one-sided or two-sided?
  - are there adjustments for multiple comparisons?
  - exact statistical test results, e.g., P values = x but not P values < x;
  - definition of 'center values' as median or average;
  - definition of error bars as s.d. or s.e.m.

Any descriptions too long for the figure legend should be included in the methods section and/or with the source data.

In the pink boxes below, please ensure that the answers to the following questions are reported in the manuscript itself. Every question should be answered. If the question is not relevant to your research, please write NA (non applicable). We encourage you to include a specific subsection in the methods section for statistics, reagents, animal models and human subjects.

B- Statistics and general methods

Please fill out these boxes ↓ (Do not worry if you cannot see all your text once you press return)

1.a. How was the sample size chosen to ensure adequate power to detect a pre-specified effect size?	No statistical methods were used to predetermine the sample size. Phosphoproteomic MS screens in each condition had ≥2 replicates. The screens are used for exploratory purposes, any selected target is further validated and looked at using a complementary method. Given the expected variation in M-tracks, we made ≥3 replicates per protein-combination (this has also been used for Romanov et al., 2017). Transcriptional analysis of CTT1 and PGM2 mRNA levels have been performed in ≥3 replicates, which is also standard practice (same is true for growth curve assays).
1.b. For animal studies, include a statement about sample size estimate even if no statistical methods were used.	No animal studies have been conducted in this work.
2. Describe inclusion/exclusion criteria if samples or animals were excluded from the analysis. Were the criteria pre-established?	No samples were specifically excluded from the study. Criteria were pre-established in terms of necessary quality.
3. Were any steps taken to minimize the effects of subjective bias when allocating animals/samples to treatment (e.g. randomization procedure)? If yes, please describe.	No such steps were required.
For animal studies, include a statement about randomization even if no randomization was used.	No animal studies were performed in this study.
4.a. Were any steps taken to minimize the effects of subjective bias during group allocation or/and when assessing results (e.g. blinding of the investigator)? If yes please describe.	No such steps were required as the methods used to collect data were not subjective in nature.
4.b. For animal studies, include a statement about blinding even if no blinding was done	No animal studies have been conducted in this work.
5. For every figure, are statistical tests justified as appropriate?	When a statistical test was performed, it was appropriately discussed and justified. In each figure, where a statistical analysis has been performed, the statistical metrics are highlighted. The tests are described in the figure legends and/or Methods section.
Do the data meet the assumptions of the tests (e.g., normal distribution)? Describe any methods used to assess it.	For Westernblot quantification and SILAC ratios normality can be assumed, which is standard practice in the field. Therefore standard tests were applied. Otherwise non-parametric tests were used.
Is there an estimate of variation within each group of data?	We do not give an estimate of variation for each group of data, but do show full distributions in respective figures.

Is the variance similar between the groups that are being statistically compared?	No similarity of variance was calculated as the used statistical methods do not require equal variance.

C- Reagents

6. To show that antibodies were profiled for use in the system under study (assay and species), provide a citation, catalog number and/or clone number, supplementary information or reference to an antibody validation profile. e.g., Antibodypedia (see link list at top right), 1DegreeBio (see link list at top right).	The following antibodies were used in this study: mouse monoclonal anti-Actin antibody (Max Perutz Labs Vienna, Monoclonal Antibody Facility, 2A3-6A5-G2), rabbit monoclonal anti-Cdc28 antibody (Sigma-Aldrich, HPA030762), mouse monoclonal anti-Cdc55 antibody (Max Perutz Labs Vienna, Monoclonal Antibody Facility, 9D3-H6), mouse monoclonal anti-HA antibody (12CA5, Max Perutz Labs Vienna, Monoclonal Antibody Facility), mouse monoclonal anti-me3K9H3 antibody (Novus, #NBP1–30141), mouse monoclonal anti-Pgk1 antibody (Invitrogen, 22C5D8), rabbit polyclonal anti-me3K9H3 antibody (Abcam, ab8898), rabbit monoclonal anti-phospho-p38 antibody (Cell Signaling, #9211). All antibodies are widely used antibodies with relevant validation data provided at manufacturer websites. All antibodies created in the in house monoclonal antibody facility have been tested for selectivity and sensitivity and have been used in previous publications: the anti-Actin antibody and anti-Cdc55 antibody have been validated previously (Frohner et al., 2020, Cell Reports, https://doi.org/10.1016/j.celrep.2020.02.035), the anti-HA antibody has been referenced in Green et al., 1982, Cell,https://doi.org/10.1016/0092-8674(82)90202-1. The anti-me3K9H3 antibody has been used in various publications from our lab and has been validated for application to M-track assays previously (Zuzuarregui et al., 2012, Nature Methods, https://doi.org/10.1038/nmeth.2017; Romanov et al., 2017, Science Signaling, https://doi.org/10.1126/scisignal.aag2435).
7. Identify the source of cell lines and report if they were recently authenticated (e.g., by STR profiling) and tested for mycoplasma contamination.	Cell lines were not used in this study.

* for all hyperlinks, please see the table at the top right of the document

D- Animal Models

8. Report species, strain, gender, age of animals and genetic modification status where applicable. Please detail housing and husbandry conditions and the source of animals.	No animal studies have been conducted in this work.
9. For experiments involving live vertebrates, include a statement of compliance with ethical regulations and identify the committee(s) approving the experiments.	No experiments involving live vertebrates have been conducted in this study.
10. We recommend consulting the ARRIVE guidelines (see link list at top right) (PLoS Biol. 8(6), e1000412, 2010) to ensure that other relevant aspects of animal studies are adequately reported. See author guidelines, under 'Reporting Guidelines'. See also: NIH (see link list at top right) and MRC (see link list at top right) recommendations. Please confirm compliance.	No animal studies have been conducted in this work.

E- Human Subjects

11. Identify the committee(s) approving the study protocol.	No human material was used in this study.
12. Include a statement confirming that informed consent was obtained from all subjects and that the experiments conformed to the principles set out in the WMA Declaration of Helsinki and the Department of Health and Human Services Belmont Report.	No human material was used in this study.
13. For publication of patient photos, include a statement confirming that consent to publish was obtained.	No photos or data on human patients were used in this study.
14. Report any restrictions on the availability (and/or on the use) of human data or samples.	No samples of human origin or data on human patients were used in this study.
15. Report the clinical trial registration number (at ClinicalTrials.gov or equivalent), where applicable.	No human material was used in this study.
16. For phase II and III randomized controlled trials, please refer to the CONSORT flow diagram (see link list at top right) and submit the CONSORT checklist (see link list at top right) with your submission. See author guidelines, under 'Reporting Guidelines'. Please confirm you have submitted this list.	No human material was used in this study.
17. For tumor marker prognostic studies, we recommend that you follow the REMARK reporting guidelines (see link list at top right). See author guidelines, under 'Reporting Guidelines'. Please confirm you have followed these guidelines.	No human material was used in this study.

F- Data Accessibility

18: Provide a "Data Availability" section at the end of the Materials & Methods, listing the accession codes for data generated in this study and deposited in a public database (e.g. RNA-Seq data: Gene Expression Omnibus GSE39462, Proteomics data: PRIDE PXD000208 etc.) Please refer to our author guidelines for 'Data Deposition'.<br><br>Data deposition in a public repository is mandatory for:<br>a. Protein, DNA and RNA sequences<br>b. Macromolecular structures<br>c. Crystallographic data for small molecules<br>d. Functional genomics data<br>e. Proteomics and molecular interactions	We have provided the Data Availability section and followed the respective guidelines. Datasets produced in this study and the scripts used for data analysis are available in the following databases:<br>•Phospho-proteomic MS data: PRIDE PXD019646 (http://www.ebi.ac.uk/pride/archive/projects/PXD019646)<br>•Python scripts used for data analysis: GitHub (https://github.com/hollenstein/sourcecode_cdc55)
19. Deposition is strongly recommended for any datasets that are central and integral to the study; please consider the journal's data policy. If no structured public repository exists for a given data type, we encourage the provision of datasets in the manuscript as a Supplementary Document (see author guidelines under 'Expanded View' or in unstructured repositories such as Dryad (see link list at top right) or Figshare (see link list at top right).	We uploaded the mass spectrometry data (along with specifications on the searches) to the PRIDE repository. All Supplementary Material is included in the upload of the manuscript to the journal.
20. Access to human clinical and genomic datasets should be provided with as few restrictions as possible while respecting ethical obligations to the patients and relevant medical and legal issues. If practically possible and compatible with the individual consent agreement used in the study, such data should be deposited in one of the major public access-controlled repositories such as dbGAP (see link list at top right) or EGA (see link list at top right).	No human material was used in this study.
21. Computational models that are central and integral to a study should be shared without restrictions and provided in a machine-readable form. The relevant accession numbers or links should be provided. When possible, standardized format (SBML, CellML) should be used instead of scripts (e.g. MATLAB). Authors are strongly encouraged to follow the MIRIAM guidelines (see link list at top right) and deposit their model in a public database such as Biomodels (see link list at top right) or JWS Online (see link list at top right). If computer source code is provided with the paper, it should be deposited in a public repository or included in supplementary information.	No models have been created in this study.

G- Dual use research of concern

22. Could your study fall under dual use research restrictions? Please check biosecurity documents (see link list at top right) and list of select agents and toxins (APHIS/CDC) (see link list at top right). According to our biosecurity guidelines, provide a statement only if it could.	Our study does not fall under dual use research restrictions.
